# Phosphorylation of PHF2 by AMPK releases the repressive H3K9me2 and inhibits cancer metastasis

**DOI:** 10.1038/s41392-022-01302-6

**Published:** 2023-03-06

**Authors:** Ying Dong, Hao Hu, Xuan Zhang, Yunkai Zhang, Xin Sun, Hanlin Wang, Weijuan Kan, Min-jia Tan, Hong Shi, Yi Zang, Jia Li

**Affiliations:** 1 State Key Laboratory of Drug Research, Shanghai Institute of Materia Medica, Chinese Academy of Sciences, Shanghai, 201203 China; 2 University of Chinese Academy of Sciences, Beijing, 100049 China; 3 School of Pharmacy, Henan University, Kaifeng, 475004 China; 4 Department of Pharmacology, Fudan University, Shanghai, 201203 China; 5 Department of Anesthesiology, Shanghai Pulmonary Hospital, School of Medicine, Tongji University, Shanghai, 200433 China; 6 Lingang laboratory, Shanghai, 201203 China; 7 School of Pharmaceutical Science and Technology, Hangzhou Institute for Advanced Study, University of Chinese Academy of Sciences, Hangzhou, 310024 China; 8 Shandong Laboratory of Yantai Drug Discovery, Bohai Rim Advanced Research Institute for Drug Discovery, Yantai, Shandong 264117 China; 9 Open Studio for Druggability Research of Marine Natural Products, Pilot National Laboratory for Marine Science and Technology (Qingdao), 1 Wenhai Road, Aoshanwei, Jimo, Qingdao, 266237 China

**Keywords:** Metastasis, Epigenetics, Target identification

## Abstract

Epithelial to mesenchymal transition (EMT) plays a crucial role in cancer metastasis, accompanied with vast epigenetic changes. AMP-activated protein kinase (AMPK), a cellular energy sensor, plays regulatory roles in multiple biological processes. Although a few studies have shed light on AMPK regulating cancer metastasis, the inside epigenetic mechanisms remain unknown. Herein we show that AMPK activation by metformin relieves the repressive H3K9me2-mediated silencing of epithelial genes (e.g., CDH1) during EMT processes and inhibits lung cancer metastasis. PHF2, a H3K9me2 demethylase, was identified to interact with AMPKα2. Genetic deletion of PHF2 aggravates lung cancer metastasis and abolishes the H3K9me2 downregulation and anti-metastasis effect of metformin. Mechanistically, AMPK phosphorylates PHF2 at S655 site, enhancing PHF2 demethylation activity and triggering the transcription of CDH1. Furthermore, the PHF2-S655E mutant that mimics AMPK-mediated phosphorylation status further reduces H3K9me2 and suppresses lung cancer metastasis, while PHF2-S655A mutant presents opposite phenotype and reverses the anti-metastasis effect of metformin. PHF2-S655 phosphorylation strikingly reduces in lung cancer patients and the higher phosphorylation level predicts better survival. Altogether, we reveal the mechanism of AMPK inhibiting lung cancer metastasis via PHF2 mediated H3K9me2 demethylation, thereby promoting the clinical application of metformin and highlighting PHF2 as the potential epigenetic target in cancer metastasis.

## Introduction

Lung cancer is the leading cause of cancer-related death worldwide, and most of these deaths are attributed to distant metastasis.^1^ At a certain stage of lung cancer, cancer cells infiltrate into the blood vessels and metastasize to multiple parts of the body. The most common distant metastasis is transfer to different lung lobes. In addition, organs with rich blood supply such as liver, bone and brain are also potential targets of lung cancer metastasis.^2^ Therefore, understanding the pathological mechanisms and process of lung cancer metastasis is essential to identify the weaknesses of tumor cells that can be exploited for clinical therapy.

Epithelial to mesenchymal transition (EMT) has been implicated in sufficient studies as a driver in the dissemination of cancer cells.^3,4^ EMT is closely associated with the control of chromatin configuration resulting from histone modifications during the development and progression of various cancers.^5–7^ During EMT, the chromatin landscape is highly changed. Epithelial genes were stably inhibited (marked by H3K27me2/3, H3K9me2/3 and DNA methylation) from an active state (marked by histone acetylation and H3K4me3), and vice versa to activate mesenchymal gene.^8^ Therefore, numerous histone modifying enzymes have been applied into cancer metastasis and developed as drug targets.^8^

AMP-activated protein kinase (AMPK) is a heterotrimeric serine/threonine protein kinase made up of a catalytic α subunit in complex with two regulatory subunits, β and γ, acting as an important energy sensor and regulator. The activation of AMPK plays multiple roles in various cellular processes via phosphorylating downstream substrates, including metabolism, autophagy, aging, cell motility and cellular stress resistance.^9–12^ In addition to its canonical function to govern energy homeostasis, accumulating evidences indicate that AMPK plays crucial part in tumorigenesis and a few studies shed light on the regulatory role of AMPK in cancer metastasis.^13^ AMPK was reported to phosphorylate EZH2, a H3K27me3 methyl transferase, to suppress tumorigenesis and PDHA, the subunit of pyruvate dehydrogenase, was also found as a direct substrate of AMPK to regulate breast cancer metastasis.^14,15^ Even though AMPK has been preliminary implicated in cancer metastasis, its mechanistic role in epigenetic regulation which plays critical part in cellular signal transduction remains unclear. Hence, we were interested in exploring whether AMPK could phosphorylate a histone post-translational modifier to regulate lung cancer metastasis.

Herein, we found that AMPK activation epigenetically relieves the H3K9me2 mediated silencing of epithelial genes and inhibits lung cancer metastasis. Mechanistically, we identified PHF2 (PHD finger protein 2), a Jumonji C domain-containing histone-lysine demethylase (KDM) that removes H3K9me2,^16–18^ as a direct phosphorylation substrate of AMPK. AMPK phosphorylates PHF2 at S655 to enhance its histone demethylase activity and trigger epigenetic reprogramming of epithelial genes, thus suppressing lung cancer metastasis. Consistent with these results, the AMPK activator metformin suppresses lung cancer metastasis via the AMPK-PHF2 axis. Importantly, higher PHF2-S655 phosphorylation level correlates with better survival in lung cancer patients. Together, these findings demonstrate a mechanism of AMPK downregulating H3K9me2 modification and provide new insights of PHF2 as the potential epigenetic target in lung cancer metastasis, thereby promoting the clinical application of metformin into suppressing tumor development.

## Results

### AMPK activation inhibits lung cancer metastasis and releases the repressive H3K9me2 mark on epithelial genes

**Fig. 1:**
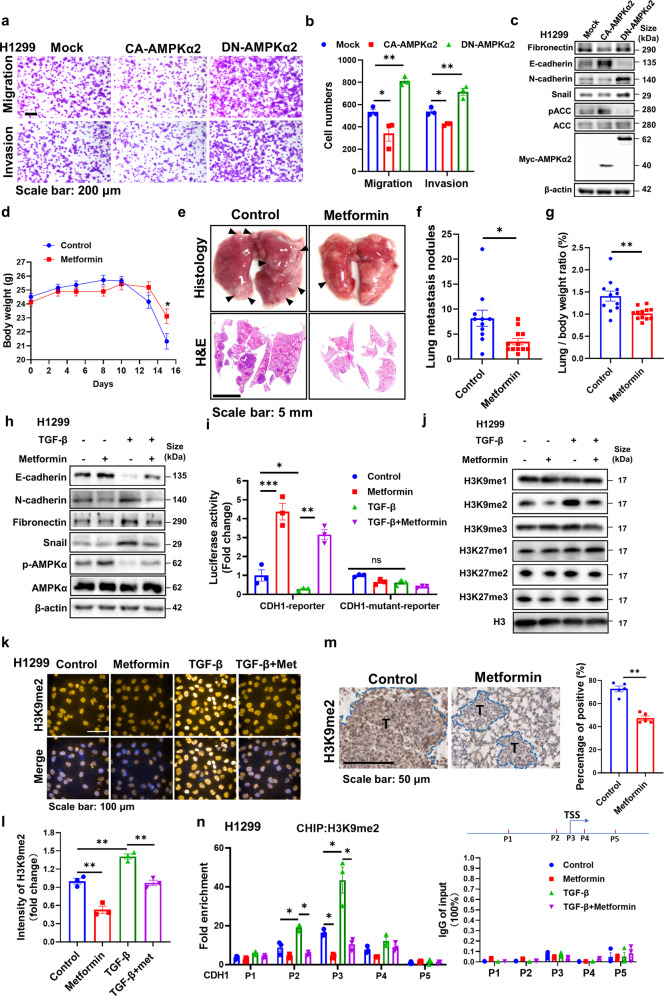
AMPK activation inhibits lung cancer metastasis and releases the repressive H3K9me2 on the promoters of epithelial genes. **a**, **b** Representative images and quantitative results of the migration and invasion in DN-AMPKα2 and CA-AMPKα2 overexpressed H1299 cells. **c** Western blot analysis of WCLs derived from DN-AMPKα2 and CA-AMPK α2 transfected H1299 cells. β-actin served as loading control. **d** Mean body weight analysis acquired at the indicated time points after orthotopic Lewis lung carcinoma (LLC) transplantation in C57BL/6 followed with metformin (250 mg/kg) treatment (*n* = 11, 12 for each group). **e** Representative images of lung nodules and H&E stained histological sections of lungs from of C57BL/6 mice acquired 15 days after orthotopic transplantation of LLC. **f**, **g** Quantitative results of lung nodules and lung/body ratio from corresponding mice. **h** Western blot analysis of whole-cell lysates (WCLs) derived from H1299 stimulated by TGF-β (5 ng/mL), combining with metformin treatment (1 mM) for 24 h. β-actin served as loading control. **i** The luciferase reporter assay containing a section of the CDH1 promoter was applied in H1299 with TGF-β (5 ng/mL) or metformin treatment (1 mM) for 24 h. CDH1 promoter luciferase values are normalized to CellTiter Glo values. **j** Western blot analysis of WCLs derived from H1299 stimulated by TGF-β (5 ng/mL), combining with metformin treatment (1 mM) for 72 h. H3 served as loading control. **k-l** Representative immunostaining images and quantitative results of the H3K9me2 intensity in TGF-β (5 ng/mL) or metformin (1 mM) treated H1299 cells for 72 h. **m** Representative immunohistochemical images and quantitative results of H3K9me2 in metformin treated lung cancer tissues. **n** CHIP-assay evaluating the recruitment of H3K9me2 to the CDH1 promoter in TGF-β (5 ng/mL) or metformin (1 mM) treated H1299 cells for 24 h with several primers around transcriptional start site (TSS). The expression levels of each IgG group of input (%) was less than 0.1%. A pattern diagram of the position of P1-P5 on the CDH1 gene promoter was shown. All error bars represent mean ± SEM. Statistical analyses were made using unpaired t-test (**f, g, m**) or one-way ANOVA (**l**) or two-way ANOVA (**b, i, n**) followed by multiple comparisons of fisher’s LSD tests with two tailed distribution. Statistical significance was determined at *p < 0.05, **p < 0.01, ***p < 0.001

To better define the epigenetic regulatory role of AMPK in cancer metastasis, we intended to focus on investigating AMPKα2 subunit which prefers nuclear localization than AMPKα1.^19^ We found that AMPKα2 deficiency enhanced the migration and invasion of the lung cancer cell H1299 in the transwell assay (Supplementary Fig. 1a, b). The expression of the epithelial marker E-cadherin (gene name, CDH1) was reduced while mesenchymal markers, N-cadherin, Fibronectin, and snail were increased caused by AMPKα2 loss (Supplementary Fig. 1c). Similarly, a dominant negative form of AMPKα2 (DN-AMPKα2) enhanced cell migration, invasion, and EMT associated markers, while constitutively active form of AMPKα2 (CA-AMPKα2) plasmid reversed these phenotypes (Fig. 1a–c). These results implied that AMPKα2 activation might suppress lung cancer metastasis.

Metformin, an AMPK agonist, is gradually emerging as a repurposed anticancer agent.^20,21^ A few clinical trials have also identified that metformin improves the progression-free survival and distant metastasis-free survival in lung cancer patients, but the underlying mechanisms remain elusive.^22–24^ We hypothesized that metformin treatment would inhibit lung cancer metastasis via AMPK activation. Firstly, to define its effect on lung cancer metastasis in vivo, we utilized an orthotopic Lewis lung carcinoma (LLC) transplantation model. Generally, the body weight loss changes can reflect the degree of tumor metastasis. We found the weight loss in the metformin treated group was significantly lower than that in the control group at day 15, suggesting that metformin might slow down tumor metastasis (Fig. 1d). Consistently, mice orally treated with metformin developed significantly fewer tumor nodules compared with the control group after sacrificed (Fig. 1e, f) and the quantitation of lung/body weight ratio also reduced accordingly (Fig. 1g), indicating metformin inhibited lung cancer metastasis in vivo. In vitro, the transwell and wound healing assay showing the cell migratory and invasive capabilities of H1299 were significantly reduced by metformin in the dose-dependent manner (Supplementary Fig. 1d–g). Then the key EMT-related markers examined by immunoblot showed that metformin increased E-cadherin, but reduced the expression of N-cadherin, Fibronectin, and snail under TGF-β stimulated EMT conditions (Fig. 1h). Consistent results were obtained by Quantitative real-time PCR assay (Supplementary Fig. 1h). Similarly, metformin inhibited EMT processes, migration, and invasion in another lung cancer cell line A549 (Supplementary Fig. 1i, j). And the anti-migration, anti-invasion and anti-EMT effects of metformin were almost abolished in AMPKα2 knockdown cells in vitro (Supplementary Fig. 2a, b). With this encouraging result, we applied orthotopic LLC transplantation assay in vivo and found that AMPKα2 deficiency remarkably enhanced lung metastasis and abolished the anti-metastasis effect of metformin (Supplementary Fig. 2c–f). Taken together, these results indicated that AMPK activation by metformin inhibits EMT processes and pulmonary metastasis.

CDH1, as a key and paradigmatic epithelial gene rather than mesenchymal genes, is often epigenetically reprogrammed during EMT, providing a more stable and long-term regulation. Also CDH1 was reported as an important global regulator, rather than just a marker of EMT.^25^ Loss of CDH1 promotes cancer metastasis via multiple downstream cascades signaling pathways.^26^ As shown in the results above, CDH1 was upregulated by metformin in transcriptional and protein levels, thus we intended to explore whether AMPK activation promotes the transcriptional activation of CDH1 promoters. A reporter plasmid expressing a portion of the CDH1 promoter fused to luciferase was applied, with the CDH1-mutant reporter including mutant E-boxes as negative control, which showed that metformin significantly induced the CDH1-driven luciferase expression (Fig. 1i). As epithelial genes are often epigenetically repressed by the chromatin landscape of H3K9me2/3 and H3K27me2/3 during EMT processes, thus we determined the effect of metformin on these histone modifications. As shown in Fig. 1j, H3K9me2 level was most strikingly upregulated during EMT processes, while metformin treatment significantly lowered that. Supporting this observation, the H3K9me2 immunostaining assay further demonstrated this phenomenon (Fig. 1k-l). The consistent result was obtained in another lung cancer cell line A549 (Supplementary Fig. 1k–m). And the lung tissues from Fig. 1e also showed reduced H3K9me2 level in the metformin treated group (Fig. 1m). Furthermore, we applied CHIP assay to determine the presence of H3K9me2 mark on the epithelial genes. As for CDH1, we designed several primers aside transcriptional start site (TSS) region and found metformin treatment most predominantly decreased H3K9me2 mark at the TSS of CDH1-bound promoters (P3 primers) (Fig. 1n). As for other epithelial genes, metformin also notably reduced the intensity of H3K9me2 level at the TSS region (Supplementary Fig. 1n). Furthermore, the reduction of H3K9me2 by metformin was also almost reversed by AMPKα2 knockdown (Supplementary Fig. 2g). H3K9 methylations, especially H3K9me2 and H3K9me3, are generally associated with heterochromatinization and transcriptional gene silencing. Thus, our results suggest that AMPK activation epigenetically downregulates H3K9me2 modification to promote epithelial genes transcription during EMT processes and inhibits lung cancer metastasis.

### PHF2 is identified to interact with AMPKα2 and PHF2 deficiency abolishes metformin downregulating H3K9me2

**Fig. 2:**
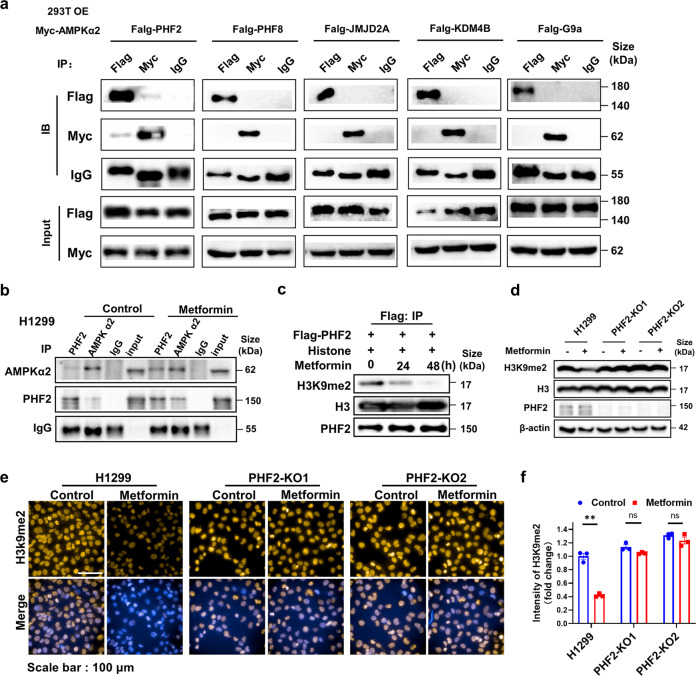
PHF2 is identified to interact with AMPKα2 and its deficiency abolishes the H3K9me2 downregulation by metformin. **a** Immunoblot analysis of whole-cell lysates (input) and anti-Flag, anti-Myc or anti-IgG immunoprecipitates derived from 293 T cells co-transfected with Myc-AMPKα2 and Flag-PHF2 or Flag-PHF8, Flag-G9a, Flag-JMJD2A, Flag-KDM4B separately. **b** Immunoblot analysis of WCLs and anti-AMPKα2, anti-PHF2 or anti-IgG endogenous immunoprecipitates derived from H1299 cells or treated with metformin. **c** In vitro demethylation assay of immunoprecipitated Flag-PHF2 treated by metformin incubated with histone for 6 h at 37 °C in the histone demethylation buffer. **d** Western blot analysis of H3K9me2 in the control and PHF2 knock-out H1299 cells with metformin treatment. β-actin served as loading control. **e**, **f** Representative immunostaining images and quantitative results of H3K9me2 in the control and PHF2 knock-out H1299 with metformin treatment. All error bars represent mean ± SEM. Statistical analyses were made using two-way ANOVA (**f**) followed by multiple comparisons of fisher’s LSD tests with two tailed distribution. Statistical significance was determined at *p < 0.05, **p < 0.01, ***p < 0.001

To explore the potential mechanism of AMPK activation downregulating H3K9me2, the H3K9me2 histone modifiers (G9a, PHF2, PHF8, JMJD2A, KDM4B)^27–31^ that reported to play a regulatory role in cancer pathogenesis were respectively performed co-immunoprecipitation with AMPKα2. Among these histone modifiers, only PHF2 was identified to interact with AMPKα2 tightly under ectopic expression conditions (Fig. 2a). Furthermore, the endogenous interaction of PHF2 and AMPKα2 was detected in H1299 cells and metformin significantly enhanced this interaction (Fig. 2b), suggesting that the AMPK activation mediated H3K9me2 downregulation might associated with PHF2.

PHF2, a member of the Jumonji C family, demethylates H3K9me2 and subsequently participates in epigenetic regulation of transcription.^17^ We next explore whether metformin mediated H3K9me2 downregulation via PHF2. We transfected Flag-tagged PHF2 plasmid into H1299 and treated with metformin at indicated time followed by the immunoprecipitation with Flag antibody for in-vitro demethylation assay, showing that metformin remarkably upregulated the H3K9me2 demethylation activity of PHF2 (Fig. 2c). And in PHF2 knock-out H1299, metformin almost abolished the ability of reducing H3K9me2 levels (Fig. 2d–f), implying that metformin mediated AMPK activation downregulates H3K9me2 modification via PHF2.

### Genetic deletion of PHF2 aggravates lung cancer metastasis and abolishes the anti-metastasis effect of metformin

PHF2 has been reported as a tumor suppressor associated with p53 in colon cancer,^28,32,33^ whereas whether it is related with lung cancer metastasis remains unknown. Thus, before further demonstrating AMPK activation inhibits lung cancer metastasis via PHF2, we were required to confirm whether PHF2 presents the ability to inhibit lung cancer metastasis via demethylation activity.

**Fig. 3:**
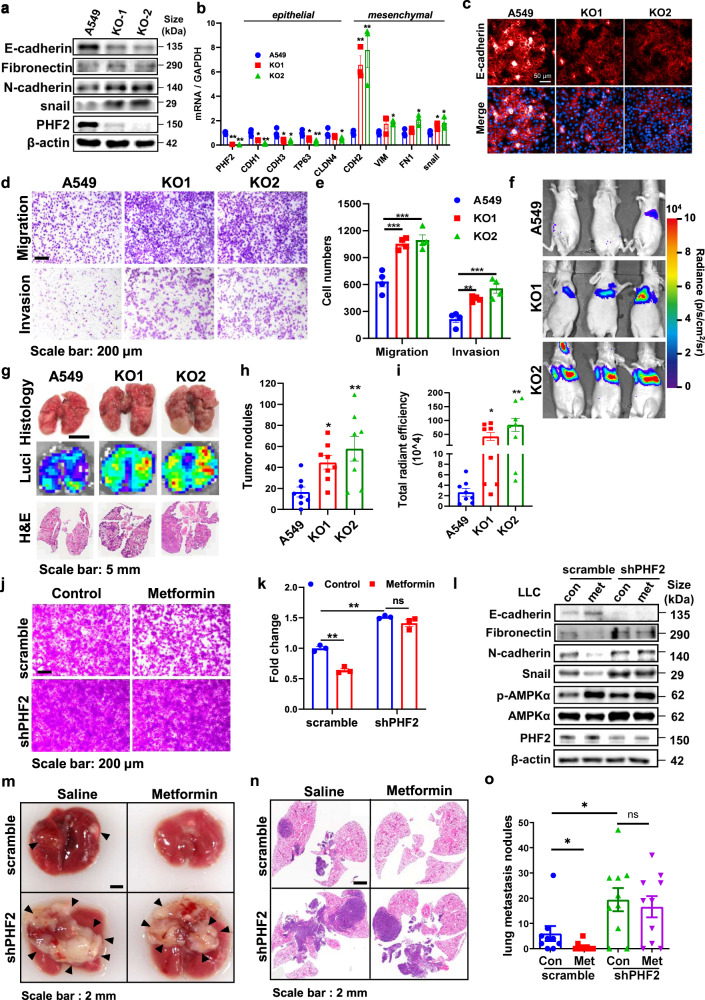
PHF2 deficiency of PHF2 aggravates lung cancer metastasis and abolishes the anti-metastasis effect of metformin. **a** Western blot analysis of whole-cell lysates (WCLs) derived from A549 and PHF2 knockout cells. β-actin served as loading control. **b** Quantitative real-time PCR analysis of PHF2 mRNA levels in A549 and PHF2 knockout cells. **c** Immunostaining results of E-cadherin expression levels of A549 and PHF2 knockout cells. **d**, **e** Representative images and quantitative results of the migration and invasion of A549 and PHF2 knockout cells. **f** Representative bioluminescent images of lung tumor in Balb/c nude mice intravenously injected with A549-control, KO-1, and KO-2 cells. **g** Representative images of lung nodules, bioluminescent lung tumor and H&E staining of indicated group of mice. **h**, **i** Quantitative results of total radiant efficiency and lung metastasis nodules from corresponding mice. **j**–**k** Representative images and quantitative results of the migration in the scramble and shPHF2 stable cell lines LLC followed with metformin treatment. **l** Western blot analysis of WCLs derived from the scramble and shPHF2 stable cell lines LLC followed with metformin treatment. β-actin served as loading control. **m** Representative images of lung nodules of C57BL/6 mice acquired 21 days after orthotopic transplantation of the scramble and shPHF2 stable cell lines LLC followed with metformin treatment via oral every day. **n** Representative images of H&E stained histological sections of lungs from C57BL/6. **o** Quantitative results of lung nodules from corresponding mice (*n* = 9, 10 each group). All error bars represent mean ± SEM. Statistical analyses were made using one-way ANOVA (**h**, **i**) or two-way ANOVA (**b**, **e**, **k**, **o**) followed by multiple comparisons of fisher’s LSD tests with two tailed distribution. Statistical significance was determined at *p < 0.05, **p < 0.01, ***p < 0.001

We generated PHF2 knockout A549 and H1299 cell lines (KO1 and KO2 cells) with CRISPR-Cas9 to evaluate its effect on lung cancer metastasis. Firstly, EMT associated markers and transcription factors were studied. As shown in Fig. 3a, b, PHF2 knockout led to the enhanced expression of mesenchymal markers and transcription factors, but reduced the expression of epithelial markers. Consistently, immunofluorescence analysis confirmed our observation that PHF2 knockout cells displayed reduced E-cadherin (Fig. 3c). Then in the transwell and wound healing assay, PHF2 deficiency showed much more migration and invasion than control cells (Fig. 3d, e and Supplementary Fig. 3a, b). Further, luciferase-labeled control or PHF2 knockout A549 cells were injected into balb/c nude mice via tail vein to explore whether PHF2 could inhibit lung metastasis in vivo. Mice injected with PHF2-KO cells developed much faster and more pulmonary metastasis than mock transfectants according to the bioluminescent (BLI) imaging (Fig. 3f). Consistently, the lung metastasis nodules displayed increased number in the PHF2 knockout group after sacrificed, indicating that PHF2 deficiency enhanced lung cancer metastasis in vivo (Fig. 3g–i). Besides, we also confirmed this phenomenon in H1299 lung cancer cells in vitro and similar results were obtained (Supplementary Fig. 3c–g). And PHF2 knockout A549 and H1299 grew at a similar rate, suggesting the increased migrative and invasive capacities were not affected by cell proliferation difference (Supplementary Fig. 3h, i). Accordingly, PHF2 inhibits lung cancer cell migration, invasion, and metastasis in vitro and in vivo.

Histidine 249 predicted to be part of the Fe (II) binding site of PHF2 jmjC domain, thus H249A mutation impaired the enzyme activity.^16,34^ H249A mutant was applied to identify the importance of demethylation activity for PHF2 inhibiting lung cancer metastasis. We found that H249A mutant dampened the ability of PHF2 to inhibit lung cancer cell migration, invasion and EMT, indicating the demethylase activity of PHF2 is essential for its anti-metastasis function (Supplementary Fig. 3j–l).

As shown in Fig. 3a–c, the epithelial gene CDH1 was much more downregulated by PHF2 deficiency compared with others, we thus speculate CDH1 gene might be a crucial downstream target of PHF2. We applied functional rescuing experiments in PHF2 knock-out cells and found that CDH1 almost rescued the enhanced lung cancer cell migration and invasion caused by PHF2 deficiency (Supplementary Fig. 3m–o). These functional phenotypes of PHF2 further supports the hypothesis that metformin mediated H3K9me2 downregulation on epithelial genes and the anti-metastasis effect might associated with PHF2.

Next, we intended to reveal whether PHF2 played an essential role in the anti-metastasis function of metformin. We treated the scramble and shPHF2 stable cell lines LLC cells with metformin, and found metformin significantly suppressed the migration of scramble cells instead of shPHF2 (Fig. 3j, k). Consistently, the western bot assay reviewed similar changes of EMT related markers (Fig. 3l). To better verify this result in vivo, scramble and shPHF2 stable cell lines LLC were orthotopic implanted into mice, followed by metformin treatment. As shown in Fig. 3m–o, metformin treatment significantly attenuated lung cancer metastasis of scramble group, while PHF2 deficiency abolished the anti-metastasis effect of metformin. Therefore, PHF2 is essential for metformin inhibiting lung cancer metastasis.

### AMPK directly phosphorylates PHF2 at S655 site

**Fig. 4:**
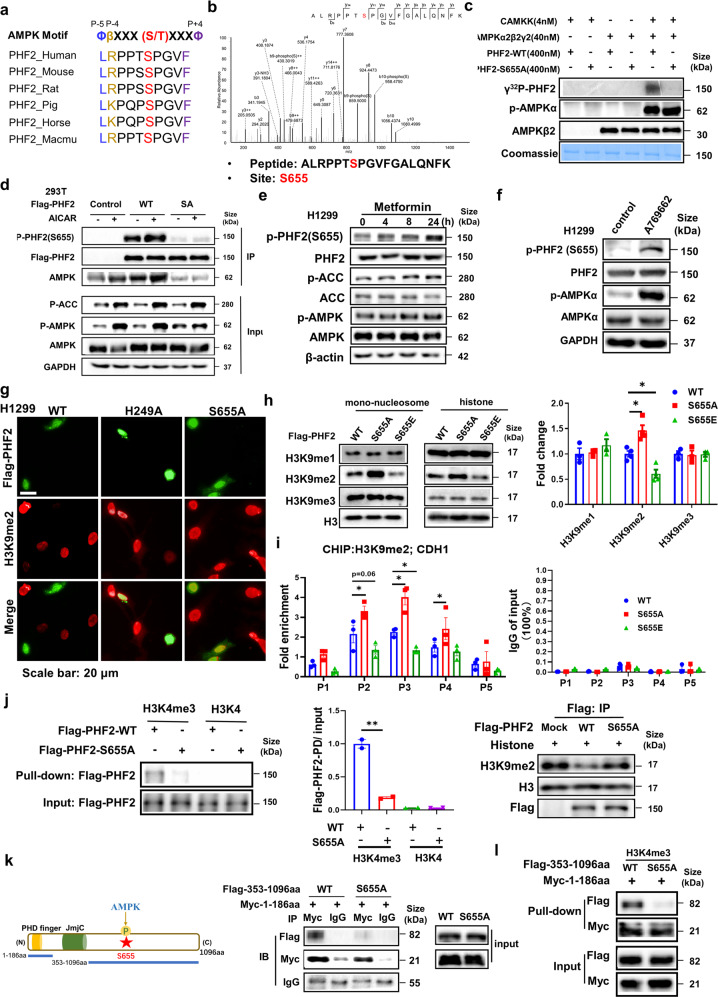
AMPK mediated PHF2-S655 phosphorylation enhances the H3K9me2 demethylation activity of PHF2 and promotes PHD domain to bind with H3K4me3. **a** The conserved amino acid sequence surrounding S655 matches the consensus AMPK phosphorylation motif. **b** Phosphorylation mass spectrometry of the bacterial expressed recombinant PHF2 incubated with pre-phosphorylated AMPKα2β2γ2 complex in the kinase buffer. **c** In vitro kinase assays with bacterially purified recombinant PHF2-WT and S655A mutant incubated with activated or inactivated AMPKα2β2γ2 complex in the kinase buffer incorporated of γ-^32^P-ATP. **d** Immunoblot analysis of WCLs and anti-Flag immunoprecipitates derived from 293 T cells transfected with flag-PHF2-WT or S655A for 48 hours followed with 1 mM metformin treatment. GAPDH served as loading control. **e** H1299 cell treated with 1 mM metformin at indicated time were subjected for immunoblot analysis. β-actin served as loading control. **f** H1299 cell treated with A769662 (200 μM for 4 h) were subjected for immunoblot analysis. GAPDH served as loading control. **g** Immunostaining results of H3K9me2 and Flag-PHF2 expression levels in PHF2-WT, H249A and S655A overexpressed H1299 cells. **h** Immunoblot analysis and quantitative results of eukaryotic exogenous expressed Flag-PHF2-WT/S655A/S655E incubated with mono-nucleosome or histone for 6 h in 37 °C in the histone demethylation buffer. H3 served as loading control. **i** CHIP-qPCR analysis of H3K9me2 marks in the promoter of CDH1 in PHF2 - WT/S655A/S655E stable cell lines of H1299. The expression level of each IgG group of input (%) was less than 0.1%. **j** The histone peptide pull-down assay and quantitative results of the nuclear extracts from Flag-PHF2-WT or S655A overexpressed 293 T cells with H3K4me3 or H3K4 peptides (left and middle). And the in-vitro demethylation assay of immunoprecipitated Flag-PHF2-WT or S655A mutant incubated with histone for 6 h at 37 °C in the histone demethylation buffer was applied meanwhile (right). **k** The schematic of PHF2 function domains and the truncation of fragments (upper). And immunoblot analysis of whole-cell lysates (input) and anti-Myc or anti-IgG immunoprecipitates derived from 293 T cells co-transfected with Flag-353-1096aa (WT or S655A) and Myc-1-186aa. **l** The histone peptide pull-down assay of the nuclear extracts from Flag-353-1096aa (WT or S655A) and Myc-1-186aa co-overexpressed 293 T cells with H3K4me3 peptides. All error bars represent mean ± SEM. Statistical analyses were made using one-way (**j**) or two-way ANOVA (**h**, **i**) followed by multiple comparisons of fisher’s LSD tests with two-tailed distribution. Statistical significance was determined at *p < 0.05, **p < 0.01, ***p < 0.001

The above results interested us to further explore the molecular mechanism by which metformin activating AMPK regulates the demethylation activity of PHF2. We hypothesized PHF2 was the phosphorylation substrate of AMPK kinase. Firstly, we applied “PhosphoMotif Finder” of Human Protein Reference Database (HPRD) to find a potential AMPK substrate consensus motif, where the evolutionally conserved PHF2-S655 site was identified. We then examined the protein sequence of PHF2 and identified a potential AMPK substrate consensus motif aside the evolutionally conserved S655 site, suggesting that AMPK might phosphorylate it (Fig. 4a). To exactly determine this phosphor site, the phosphorylation mass spectrometry was applied and the S655 site of PHF2 was identified again when incubated with phosphorylated AMPKα2β2γ2 complex (Fig. 4b). Further, in-vitro kinase assay was performed with the purified bacterial expressed wildtype PHF2 or the mutant of S655A incubated with the pre-phosphorylated AMPKα2β2γ2 complex in the kinase buffer (Fig. 4c and Supplementary Fig. 4a). As shown in Fig. 4c, wildtype PHF2 instead of the S655A mutant, could be efficiently phosphorylated by the pre-phosphorylated AMPKα2β2γ2 complex. To further examine the PHF2 phosphorylation, we developed a phosphor-specific antibody of PHF2 phospho-S655, which showed non-reaction with S655A mutant, thus validating the antibody specificity (Fig. 4d). Furthermore, we expressed the Flag-tagged wildtype PHF2 and S655A mutant plasmids separately into cells and treated with metformin followed by immunoprecipitation. Metformin treatment significantly increased the S655 phosphorylation of wildtype PHF2 and enhanced the interaction with AMPK, while S655A mutant largely impaired the phosphorylation and interaction (Fig. 4d). Similarly, AICAR (AMPK activator) treatment increased S655 phosphorylation of exogenous expressed PHF2-WT instead of S655A (Supplementary Fig. 4b). To better monitor the S655 phosphorylation at endogenous level, metformin, AICAR or the specific AMPK agonist A769662 were applied and those treatment separately increased the S655 phosphorylation of PHF2. (Fig. 4e, f and Supplementary Fig. 4c). Furthermore, under glucose deprivation pathophysiological conditions, AMPK was activated, which induced PHF2-pS655 phosphorylation (Supplementary Fig. 4d). To classify the specific AMPKα subunit responsible for PHF2-pS655 phosphorylation, we applied siAMPKα1 and siAMPKα2 into A769662 stimulated H1299 cells, showing that PHF2-S655 phosphorylation was almost abolished by AMPKα2 deficiency rather than AMPKα1 (Supplementary Fig. 4e). Similar results were obtained in metformin induced PHF2-pS655 phosphorylation (Supplementary Fig. 4f), implying that AMPKα2 was mainly responsible for phosphorylating PHF2.

In Fig. 2a, PHF2 was found to interact with AMPKα2 while AMPKα1 didn’t shown any interaction (Supplementary Fig. 4g). Next, we sought to identify the binding domains of PHF2 and AMPKα2. Among the four fragments of PHF2, the 608–820aa truncation containing S655 site bound to AMPKα2 reciprocally (Supplementary Fig. 4e). And S655A mutation disturbed the interaction of AMPKα2 and PHF2 exogenously (Supplementary Fig. 4f). Altogether, these results provide a strong support for our hypothesis that AMPK directly phosphorylates PHF2 at S655 site, which also enhanced their interaction.

### S655 phosphorylation enhances PHF2 mediated H3K9me2 demethylation activity and promotes PHD domain to bind with H3K4me3

Given the previously reported role of PHF2 as an H3K9me2 demethylase, whether AMPK mediated PHF2-S655 phosphorylation regulates its demethylase activity is worth exploring. Flag-tagged PHF2-WT, S655A and H249A (as negative control) plasmids were introduced into 293 T cells separately followed with co-immunostaining of H3K9me2 histone marks and Flag-PHF2. As shown in Fig. 4g, PHF2-WT demethylated H3K9me2 marks where Flag-PHF2 located, while S655A similar with H249A mutant had only marginal effect on that. Then in-vitro lysine demethylation assay was applied and the purified Flag-tagged recombinant PHF2 protein with S655E mutant showed much better demethylation activity of H3K9me2 than WT, while S655A mutant presented less activity (Fig. 4h). The similar results of S655A were obtained via incubation with endogenous immunoprecipitated PHF2 with histone (Supplementary Fig. 5a, b). Further, ChIP assay was performed to evaluate the effect of S655 phosphorylation on the H3K9me2-mediated silencing of epithelial genes in lung cancer cell. As for CDH1, S655A mutant H1299 cells remarkably upregulated the H3K9me2 level, while S655E further decreased this modification compared with PHF2-WT especially at the TSS region (P3 primers) (Fig. 4i). As for other epithelial genes, S655E mutant that mimic phosphorylation also notably reduced the intensity of H3K9me2 level at the TSS region (Supplementary Fig. 5c). The similar results were obtained in A549 cells (Supplementary Fig. 5d).

Having known that the PHD finger domain binding H3K4me3 is essential for PHF2 mediated H3K9me2,^18^ we speculated that whether the S655 phosphorylation affects the PHD binding with H3K4me3. In the histone peptide pull-down assay, PHF2-WT presented strong binding with H3K4me3, while S655A mutant largely reduced this interaction, suggesting the S655 phosphorylation promotes the correlation of the PHD domain with H3K4me3 (Fig. 4j). And the H3K9me2 level was performed side-by-side (Fig. 4j). To further elucidate this, we constructed two plasmid fragments of PHF2, the 1–186aa truncation containing PHD domain and the 353–1096aa including S655 site (Fig. 4k). We hypothesized the two fragments maybe adjacent in the three-dimension structure, thus affecting the interaction with H3K4me3. The two fragments were separately labelled with Myc or Flag tag and co-expressed into 293T, presenting the result that wildtype 353–1096aa truncation interacted tightly with PHD domain while S655A mutant weakened the interaction (Fig. 4k). Accordingly, the two fragments were applied into the histone-peptide pull-down assay, which also showed less interaction with S655A mutant (Fig. 4l). These results further suggest that the S655A mutant disturbs the interaction of the PHD domain with H3K4me3, but the detailed mechanism requires further structure analysis.

Altogether, our results suggest that AMPK mediated PHF2-S655 phosphorylation enhances the H3K9me2 demethylation activity of PHF2 and promotes PHD domain to bind with H3K4me3.

### α-KG metabolites partly contribute to metformin enhanced PHF2 demethylation activity

Given the fact that TCA cycle intermediate α-ketoglutarate (α-KG) is a required co-factor for JmjC-domain-containing histone demethylases, including PHF2 (Supplementary Fig. 5e).^17^ Moreover, AMPK is a critical energy regulator in cell metabolism and was reported to increase the cellular α-KG levels partly via isocitrate dehydrogenases-2 (IDH2),^35,36^ one of rate-limiting enzymes catalyzing α-KG generation, interested us to further explore whether AMPK activation promotes the demethylation activity of PHF2 associated with the increasement of α-KG. In this way, we firstly confirmed that metformin treatment remarkably enhanced the α-KG levels in H1299 and the deletion of IDH2 partly abolished this increasement (Supplementary Fig. 5f–h). Then GFP-tagged PHF2 was overexpressed in IDH2 knock-down H1299 cells followed by metformin treatment, showing the result that IDH2 deficiency partly weakened the metformin regulated H3K9me2 demethylation activity of PHF2 (Supplementary Fig. 5i). Collectively, these data suggest that AMPK activation enhanced the demethylation activity of PHF2 partly associated with the regulation of α-KG metabolites, but also requires further study.

### PHF2-S655 phosphorylation suppresses lung cancer metastasis

**Fig. 5:**
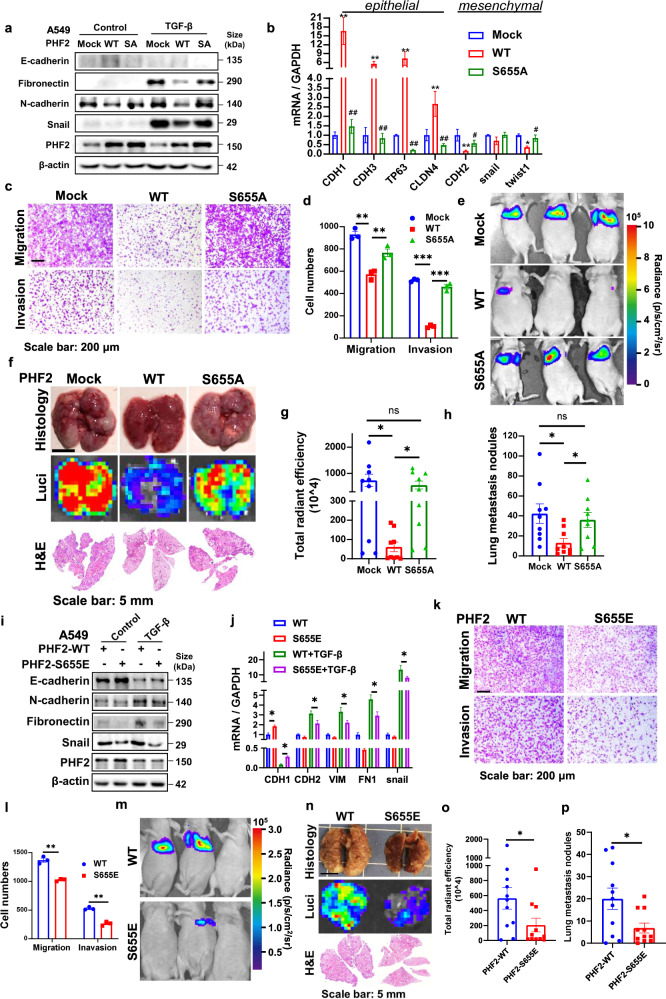
AMPK mediated PHF2-S655 phosphorylation suppresses lung cancer metastasis. **a** Western blot analysis of WCLs derived from A549 stable cell lines mock, PHF2-WT and PHF2-S655A stimulated with or without TGF-β1 for 24 h. β-actin served as loading control. **b** Quantitative real-time PCR analysis of WCLs derived from A549 stable cell lines (mock, PHF2-WT and PHF2-S655A). “*” compared with Mock. “#” compared with WT. **c**, **d** Representative images and quantitative results of the migration and invasion in A549 stable cell lines (mock, PHF2-WT and PHF2-S655A). **e** Representative bioluminescent images of lung cancer in Balb/c nude mice intravenously injected with A549-mock, PHF2-WT and PHF2-S655A cells. **f** Representative images of lung nodules, bioluminescent lung tumor and H&E staining of indicated group of mice. **g**, **h** Quantitative results of total radiant efficiency and lung metastasis nodules from corresponding mice. **i** Western blot analysis of WCLs derived from A549 stable cell lines PHF2-WT and S655E stimulated with or without TGF-β1 for 24 h. β-actin served as loading control. **j** Quantitative real-time PCR analysis of WCLs derived from A549 stable cell lines (PHF2-WT and S655E). **k**, **l** Representative images and quantitative results of the migration (24 h) and invasion (36 h) in A549 stable cell lines (PHF2-WT and S655E). **m** Representative bioluminescent images of lung cancer in Balb/c nude mice intravenously injected with PHF2-WT and PHF2-S655E cells. **n** Representative images of lung nodules, bioluminescent lung tumor and H&E staining of indicated group of mice. **o**, **p** Quantitative results of total radiant efficiency and lung metastasis nodules from corresponding mice. All error bars represent mean ± SEM. Statistical analyses were made using unpaired *t*-test (**o**, **p**) or one-way ANOVA (**g**, **h**) or two-way ANOVA (**b**, **d**, **j**, **l**) followed by fisher’s LSD tests with two tailed distribution. Statistical significance was determined at *p < 0.05, **p < 0.01, ***p < 0.001

Next, we tended to further investigate the biological function of PHF2-S655 phosphorylation in lung cancer metastasis. PHF2-WT and the mutant S655A overexpression stable cell lines were generated using lentiviral vectors in A549. Quantitative real-time PCR results and immunoblot assay showed that PHF2-WT, rather than S655A mutant, significantly inhibited the EMT processes induced by TGF-β or under basic condition (Fig. 5a, b). Correspondingly, reintroduction of PHF2-WT, instead of S655A mutant, significantly suppressed lung cancer cell migration and invasion (Fig. 5c, d). And in the wound healing assay, PHF2-WT group showed more reduced migration than S655A mutant (Supplementary Fig. 6a, b), and this was also not affected by the difference in growth rate (Supplementary Fig. 6c). To further demonstrate this in vivo, luciferase-labeled mock, PHF2-WT or S655A mutant A549 cells were injected into balb/c nude mice via tail vein. We found mice injected with PHF2-WT cells developed slower and fewer pulmonary metastasis than mock group, while S655A mutant abolished the function of suppressing lung cancer metastasis according to the bioluminescent (BLI) imaging (Fig. 5e–g). Consistently, the lung nodules after sacrificed displayed reduced number in the PHF2-WT group instead of S655A mutant (Fig. 5h). To avoid the growth rate difference in vivo, we subcutaneously injected mock, PHF2-WT or S655A mutant A549 cells into balb/c nude mice and no obvious difference of tumor volume or tumor weight was observed (Supplementary Fig. 6d–f). The similar effect of S655A mutant was also obtained in H1299 according to the EMT, transwell and wound healing assay (Supplementary Fig. 6g–i).

In addition, to better mimic the S655 phosphorylation of PHF2 mediated by AMPK, we further applied the S655E mutant and compared its effect with PHF2-WT. Firstly, EMT associated markers and transcription factors were studied. As shown in Fig. 5i, j and Supplementary Fig. 6m, the S655E mutant further suppressed EMT processes than PHF2-WT as expected. And in the transwell assay, we prolonged the experimental time to allow more cell-movement and found S655E inhibited much more migration and invasion than PHF2-WT in A549 and H1299 cells (Fig. 5k, l and Supplementary Fig. 6n, o). Then the PHF2-WT and S655E mutant were applied into the in-vivo metastasis, and the experimental condition was optimized with extended period and more cell number injected. As shown in Fig. 5m–p, S655E mutant showed much reduced lung metastasis nodules than PHF2-WT, indicating the S655E phosphorylation further enhanced the anti-metastasis effect of PHF2. To conclude, these results support a crucial role of AMPK mediated PHF2 phosphorylation at S655 in suppressing lung cancer metastasis.

### PHF2-S655A mutation abolishes the H3K9me2 downregulation and anti-metastasis effect of metformin

**Fig. 6:**
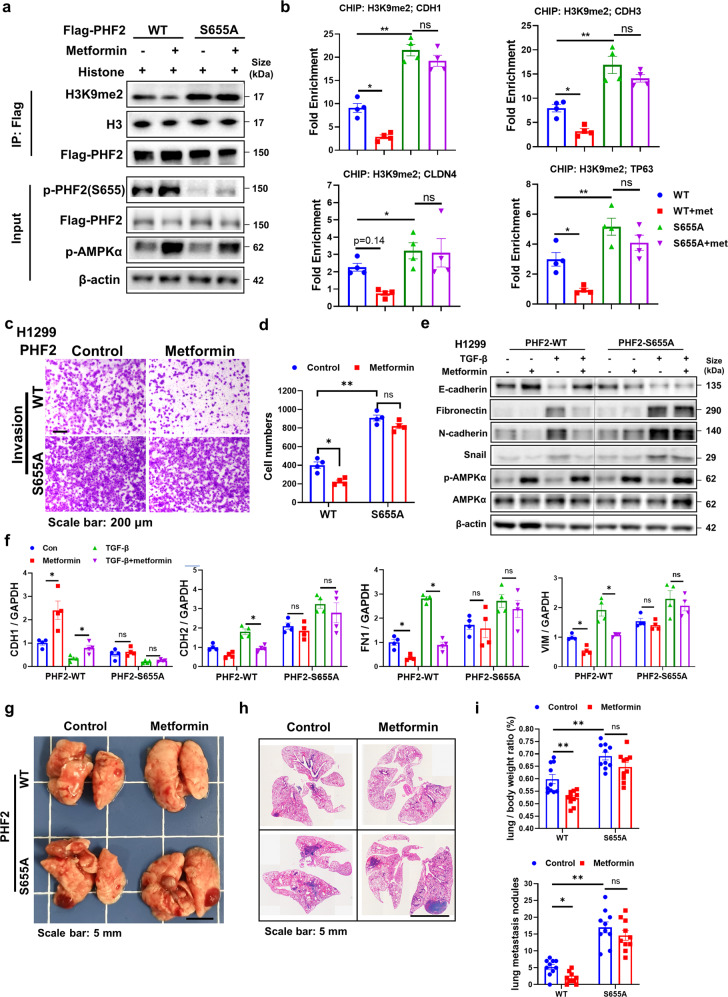
PHF2-S655A mutation abolishes the H3K9me2 downregulation and anti-metastasis effect of metformin. **a** In vitro demethylation assay of immunoprecipitated Flag-PHF2-WT or S655A treated by metformin incubated with histone for 6 h at 37 °C in the histone demethylation buffer. **b** CHIP-qPCR analysis of H3K9me2 marks in the promoter of epithelial genes including CDH1, CDH3, CLDN4, and TP63 in PHF2-WT and S655A stable cell lines in H1299 with metformin treatment. The expression level of each IgG group of input (%) was less than 0.1%. **c**, **d** Representative images and quantitative results of the invasion in PHF2-WT and S655A stable cell lines in H1299 followed with metformin treatment. **e** Western blot analysis of WCLs derived from PHF2-WT and S655A stable cell lines in H1299 treated with TGF-β1 or metformin for 24 h. β-actin served as loading control. **f** Quantitative real-time PCR analysis of WCLs derived from H1299 stable cell lines PHF2-WT and S655A treated with TGF-β1 or metformin for 24 h. **g**, **h** Representative images of lung nodules and H&E stained histological sections from C57BL/6 mice acquired 15 days after orthotopic transplantation of PHF2-WT or S655A stable cell line in LLC followed with metformin treatment. **i** Quantitative results of lung/body ratio and lung metastasis nodules from corresponding mice. All error bars represent mean ± SEM. Statistical analyses were made using one-way ANOVA(**b**) or two-way ANOVA (**d**, **f**, **i**) followed by fisher’s LSD tests with two tailed distribution. Statistical significance was determined at *p < 0.05, **p < 0.01, ***p < 0.001

Having explored the biological function of AMPK mediated PHF2-S655 phosphorylation, we next reasoned whether metformin, the AMPK activator, possesses the H3K9me2 downregulation and anti-metastasis function depending on this phosphorylation. To address this, 293T cells were overexpressed with Flag-PHF2-WT or S655A mutant separately and treated with metformin, followed by immunoprecipitation, showing that the PHF2-S655A mutation mostly abolished the H3K9me2 downregulation of metformin (Fig. 6a). The CHIP assay was also applied to demonstrate that metformin epigenetically regulated the H3K9me2 level of epithelial genes promoters mostly via the S655 phosphorylation of PHF2 (Fig. 6b).

We then treated the PHF2-WT and S655A mutant stable cell lines with metformin for transwell assay and found that metformin almost abolished the effect of anti-invasion in S655A mutant cells (Fig. 6c, d). And in the TGF-β stimulated EMT assay, metformin lost the function of upregulating E-cadherin and downregulating N-cadherin, Fibronectin, or Vimentin in S655A mutant cells (Fig. 6e, f). Inspired by the in-vitro results, we further applied the orthotopic LLC transplantation model in vivo and found in the S655A group, metformin hardly suppressed lung cancer metastasis compared with PHF2-WT group (Fig. 6g–i). Taken together, PHF2-S655 phosphorylation is essential for metformin to downregulate H3K9me2 and suppress lung cancer metastasis.

### The Validation of AMPK-PHF2-EMT axis in EGFR-mutant cell line HCC827

In order to investigate the broad application of AMPK-PHF2 axis in NSCLC, we applied another EGFR-driven NSCLC, HCC827 cell line, into study. As HCC827 is a highly epithelial cell line, which hardly migrate in vitro, we applied the following experiments in TGF-β and FGF-2 co-stimulated assay to enhance the basal migration levels.^37^ Metformin treatment significantly reduced migration and invasion induced by TGF-β and FGF-2 (Supplementary Fig. 7a, b). Also, the EMT stimulated by TGF-β and FGF-2 was also remarkably suppressed by metformin treatment (Supplementary Fig. 7c). In addition, PHF2-WT overexpression significantly reduced the migration and invasion of HCC827 compared with mock group, which was totally reversed by S655A mutant (Supplementary Fig. 7d, e). Moreover, S655E, the mimic of AMPK mediated PHF2-S655 phosphorylation, further enhanced the anti-metastasis effect of PHF2 (Supplementary Fig. 7d, e). The similar results were obtained in EMT indicators, especially E-cadherin (Supplementary Fig. 7f). These results imply that AMPK-PHF2-EMT axis is similarly active in HCC827. More wide and in-depth research on this axis worth further study in the future.

### PHF2-S655 phosphorylation strikingly reduces in lung cancer patients and the higher phosphorylation level predicts better survival

To explore the biological importance of S655 phosphorylation in lung cancer metastasis, we compared PHF2 expression and the phosphor S655 level between A549 of high mesenchymal characters and PC-9 cells with high epithelial properties. As shown in Supplementary Fig. 8a, b, the PHF2 protein expression and the S655 phosphorylation largely decreased in A549, interested us to wander whether the results are reproducible in clinical settings.

Given PHF2 was reported as tumor suppressor in previous studies, we applied The Cancer Genome Atlas (TCGA) database to explore the correlation of PHF2 expression in lung cancer. Analyses of data in TCGA revealed that PHF2 was significantly downregulated in lung adenocarcinoma (LUAD) and Lung squamous cell carcinoma (LUSC) (Supplementary Fig. 8c, d). In addition to lung cancer, TCGA database also revealed significantly reduced PHF2 levels in various primary tumors, such as breast cancer (BRCA) and kidney renal papillary cell carcinoma (KIRP) (Supplementary Fig. 8e, f). To further investigate the correlation of PHF2 and lung cancer metastasis, we found the proportion of PHF2 low-expression in patients with lymph node metastasis (N1-3) was significantly higher than that without metastasis (N0) and its expression also gradually declined with the lung cancer patients entering advanced stage (Supplementary Fig. 8g, h). More importantly, high PHF2 expression correlated well with better overall survival and relapse-free survival of lung cancer patients, suggesting PHF2 is of high clinical significance in lung cancer (Supplementary Fig. 8i, j).

**Fig. 7:**
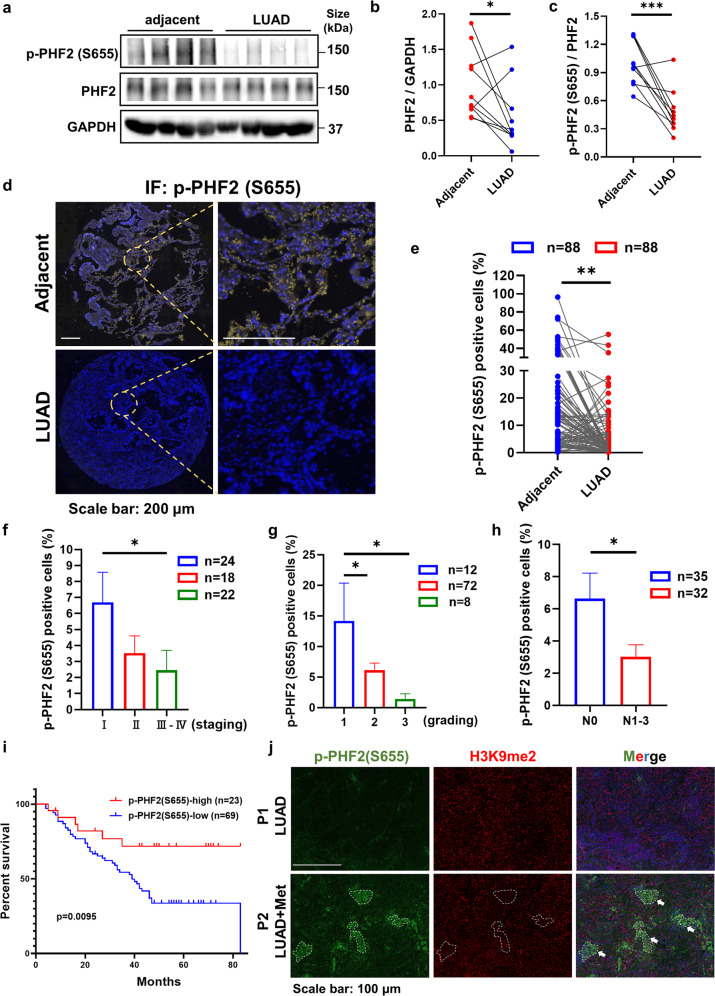
PHF2-S655 phosphorylation level strikingly reduces in lung cancer patients and the higher phosphorylation level predicts better survival. **a**–**c** Western blot analysis of WCLs derived from the lung tissues of LUAD patients with the adjacent tissues as control. And the quantitative results of PHF2 expression and S655 phosphorylation levels with Image J. GAPDH served as loading control. **d** Immunostaining results of p-PHF2(S655) in tissue microarrays generated from human LUAD primary tumor samples with the paired adjacent tissues as control. **e** Quantitative results of the proportion of p-PHF2(S655) positive cells in LUAD tissue microarrays. **f**, **g** Analyses of p-PHF2(S655) positive cells in various staging or grading of LUAD patients from the tissue microarrays. **h** Quantitative results of p-PHF2(S655) positive cells in the LUAD tissue microarrays with or without lymph node metastasis. **i** Kaplan-Meier plots showing the overall survival of patients with high or low p-PHF2(S655) level in the LUAD tissue microarrays. **j** Immunostaining results of p-PHF2(S655) and H3K9me2 level in LUAD patients with or without metformin medication history. All error bars represent mean ± SEM. Statistical analyses were made using paired *t*-test (**e**) or unpaired *t*-test (**b**, **c**, **h**) or a log-rank test (**i**) or one-way ANOVA (**f, g**) followed by multiple comparisons of fisher’s LSD tests with two-tailed distribution. Statistical significance was determined at *p < 0.05, **p < 0.01, ***p < 0.001

Next, to further investigate the clinical significance of PHF2-S655 phosphorylation in patients with lung cancer, we firstly confirmed the specificity of p-PHF2 (S655) antibody on FFPE tissue slides with blocking peptide (Supplementary Fig. 8k). We collected and applied the clinical samples into immunoblot assay to compare the S655 phosphorylation level between the adjacent and LUAD sections. As shown in Fig. 7a–c, the S655 phosphorylation relative to the protein expression was largely reduced in LUAD tissue samples. Consistently, the downregulation of PHF2-S655 phosphorylation was further demonstrated by immunohistochemical staining (IHC) (Supplementary Fig. 8l). In order to statistically analyze this result in more clinical samples, we assessed PHF2-S655 phosphorylation in tissue microarrays generated from human LUAD primary tumor samples. As shown in Fig. 7d, e, S655 phosphorylation was dramatically reduced in LUAD samples consistent with previous results. Then according to the patients’ information, we divided those into different staging or grading and found S655 phosphorylation was gradually declined with the lung cancer patients entering advanced periods (Fig. 7f, g). And the proportion of p-PHF2(S655) positive cells was significantly downregulated in patients with lymph node metastasis (N1-3) than those without metastasis (N0) (Fig. 7h). Importantly, high proportion of p-PHF2(S655) positive cells predicted better overall survival (Fig. 7i), highlighting the significance of AMPK-PHF2 axis in controlling lung cancer progression and metastasis.

We fortunately collected one LUAD case who long-term medicated with metformin and compared the p-PHF2(S655) and H3K9me2 level with another LUAD patient without metformin medical history. As shown in Fig. 7j, i the sample of LUAD patient taken with metformin, where p-PHF2(S655) positive showed much lower H3K9me2 level (marked with the white dotted line) compared with the normal LUAD case, suggesting that metformin treatment may promote the S655 phosphorylation and its demethylation activity in this clinical case study. Collectively, these data imply that PHF2-S655 phosphorylation mediated by AMPK was closely correlated with lung cancer progression clinically.

## Discussion

**Fig. 8:**
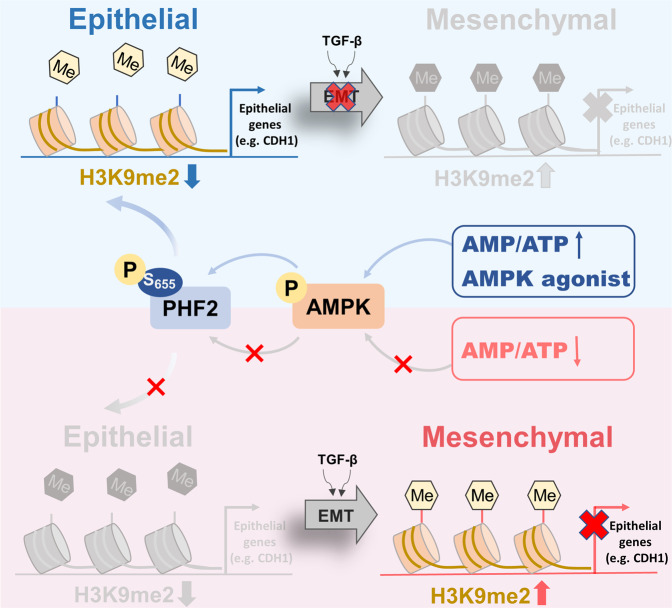
Schematic depicting the molecular mechanism through which AMPK epigenetically inhibits lung cancer metastasis. AMPK activation promotes PHF2-S655 phosphorylation, which enhances the H3K9me2 demethylation activity of PHF2 and triggers the transcription of epithelial genes, thus inhibiting EMT processes. The figure was drawn by using Figdraw

AMPK, as a conservative energy sensor, plays roles in diverse biologic processes via direct phosphorylation on various substrates.^38^ However, few epigenetic substrates of AMPK were found to control cancer metastasis where epigenetic alterations play critical roles. Herein we applied the AMPK agonist metformin and demonstrated that AMPK activation epigenetically downregulates the H3K9me2 modification of crucial epithelial gene promoters during EMT processes. PHF2, a H3K9me2 histone demethylase, was found to be the potential downstream of AMPK. And PHF2 deficiency abolished the downregulation of H3K9me2 and inhibition of lung cancer metastasis of metformin. As expected, we identified AMPK as a direct upstream kinase of PHF2 and AMPK mediated PHF2-S655 phosphorylation was critical for its demethylase activity and epigenetic reprogramming of epithelial genes during EMT processes (Schematic see Fig. 8). Therefore, AMPK-PHF2 axis serve as a signal-sensing epigenetic determinant through removal of a repressive histone methylation mark on the promoters of epithelial genes, which also provides a new mechanism for metformin suppressing lung cancer metastasis. Finally, our research highlighted the clinical significance of PHF2-S655 phosphorylation in LUAD, correlating well with the overall survival of lung cancer patients. Thus, our study not only identifies PHF2-S655 phosphorylation as a biomarker for the prediction of tumor metastasis but also provides the evidence that PHF2 activation maybe a promising strategy for tackling lung cancer metastasis.

During EMT process, the chromatin landscape is highly altered.^8^ Multiple H3K9me2/3 demethylases and methyltransferases have been gradually studied during EMT, but still some contradictory points of view inside, which may be resulted from the heterogeneity of multiple tumor cells.^39–41^ Suv39h1, a histone methyltransferase of H3K9 (H3K9MT), is highly expressed and functions as an oncogene in hepatocellular and colorectal cancer metastasis.^40,41^ G9a, a H3K9MT, promotes lung cancer metastasis via the repression the cell adhesion molecule Ep-CAM.^39^ And multiple histone-lysine demethylases (KDMs) that remove H3K9 methylation, such as JMJD1A, JMJD2B and PHF8, which have been linked with promoting tumorigenesis and EMT activity.^42–45^ However, among these studies, few histone modifiers have been found to inhibit cancer metastasis instead of promoting that. PHF2 belongs to the KDM7 histone demethylase family, locating in human chromosome 9q22.31 where multiple tumor suppressor genes accumulate.^46,47^ Accordingly, PHF2 is susceptible to mutate in various tumor cells, which has been demonstrated as a tumor suppressor and enhances p53-mediated apoptosis in chemotherapy.^28,32,33^ Besides, it was reported that PHF2 plays a crucial role in mesenchymal to epithelial transition (MET) of breast cancer, with the loss of tumor-initiating ability.^17,48^ In our study, we further expanded its function in EMT regulation and lung cancer metastasis. But one of the limitations of our study is that there is no systematic screening of all H3K9 histone modifiers regulated by AMPK, which will be further studied. As epigenetic modification is reversible, emerging therapies have been developed to regulate epigenetic abnormalities.^49^ Future study aiming to develop PHF2 activators are expected to achieve a promising efficacy for lung cancer metastasis.

How PHF2 demethylation activity is regulated by S655 phosphorylation remains not clear enough. Even though we provided the evidence that the S655A mutant disturbed the binding of PHD domain with H3K4me3, whether the S655 phosphorylation influenced the crystal structure remains to be addressed. Besides, we found that phospho-deficient mutant form of PHF2 binds less to AMPK, suggesting the S655 phosphorylation was at least necessary for the strong interaction between PHF2 and AMPK. We speculated that the S655-phosphorylation may affect its 3D spatial structure to get more available for binding AMPK. However, due to the lack of the crystal structure of PHF2, further investigation of this phenomenon is limited and worth studied in the future.

In addition, we found metformin activation enhanced the cellular α-KG levels, which works as an important co-factor for JmjC-domain-containing demethylase. And the deletion of IDH2 partly weakened the PHF2 demethylation activity enhanced by metformin, but the detailed mechanism remains comprehensive study. And whether α-KG metabolites regulating lung cancer metastasis associated with PHF2 also worth subsequent research.

In our study, we found AMPK mediated PHF2-S655 phosphorylation promoting E-cadherin transcription via enhancing its H3K9me2 demethylation. Meanwhile, the EMT transcription factors were also impacted, which drew our attention. We think it’s mainly due to the following two reasons. On the one hand, E-cadherin was also reported as an important global regulator, rather than just a marker of EMT.^25^ Loss of E-Cadherin promotes metastasis via multiple downstream transcriptional pathways.^26^ Thus, the impact of PHF2-S655 on these EMT transcription factors maybe attributed to the cascade signaling of E-cadherin. On the other hand, multiple H3K9 erasers or writers, such as PHF8, G9a and KDM4B, were reported to promote EMT transcription factors.^8^ We speculated that PHF2 may compete with these modified enzymes in spatial structure, thus preventing EMT-TFs transcriptional activation, which warrants intensive study.

AMPKα1 has been reported to phosphorylate PDHA, driving PDHc activation and TCA cycle, enabling breast cancer cells to adapt to the metastasis microenvironment.^15^ Another study reported that α-KG is involved in AMPK activation in anoikis resistance that provides pro-metastatic potential in lung cancer.^50^ While our study implied that AMPKα2 epigenetically downregulates H3K9me2 and inhibits lung cancer metastasis via phosphorylating PHF2. One the one hand, the controversial effect may due to different cellular genetic background and metabolic environment. On the other hand, AMPKα1 and AMPKα2 seem to display contradictory effect on cancer metastasis, interesting us to consider that AMPK may play different roles in diverse tumor environments and an inside balance maybe presents between the two catalytic subunits.

Intra-venous transplantation of cancer is one of the commonly used experimental methods for cancer metastasis in vivo.^51–53^ A drawback of this model system is that it fails to recapitulate the earlier stages of the metastatic cascade, such as local invasion and intravasation, while merely reflects the homing of tumor cells circulating in the bloodstream to a limited set of organs.^54^ Despite this, intra-venous transplantation has been instrumental in elucidation of tumor-host interactions required for the initial arrest and colonization at metastatic sites. The other orthotopic lewis lung carcinoma (LLC) transplantation model partially mimicked the whole metastatic process, which compensates for these defects of intra-venous injection. However, both tumor transplantation models hardly develop distal metastasis and spontaneous or genetically modified (GM) metastasis model are considered in the future.

This study identifies PHF2 as an important downstream effector of AMPK that inhibits lung cancer metastasis, highlighting PHF2-S655 phosphorylation as the potential biomarker in lung cancer metastasis and promoting the clinical application of metformin into suppressing tumorigenesis and development.

## Materials and methods

### Clinical lung cancer samples and tissue microarrays

All the patient tumor samples included in this study were retrieved from Shanghai Pulmonary Hospital and were obtained from patients undergoing surgical resection. As a rule, informed consent was obtained from all subjects and all the samples are anonymized. This study was approved by the Chinese Clinical Trial Registry (ChiCTR1900028235).

The tissue microarrays (HLugA190Su04) generated from human LUAD primary tumor samples were bought from SHANGHAI OUTDO BIOTECH company. And the subsequent p-PHF2(S655) immunostaining was performed and analyzed by WiSee Bio.

### Mice and housing conditions

Animal experiments were approved by the Shanghai Institutional Animal Care & Use Committee (IACUC) and the National Research Council Guide for the care and use of laboratory animals. Animals were housed and cared according to standard guidelines with free access to food and water. Mice experiments were respectively performed on male C57BL/6 mice (8 weeks of age) and female Balb/c nude mice (6 weeks of age, housed in specific pathogen-free environments) purchased from SHANGHAI SLAC and maintained in a 12 h light/dark cycle.

### Cell lines and culture conditions

The H1299 and HEK293T were purchased from American Type Culture Collection (ATCC). A549-luc cells were purchased from JCRB Cell Bank. The Lewis lung carcinoma (LLC) and PC-9 cells were kindly provided by Dr. Jian Ding. HEK293T and PC-9 cell lines, were cultured in Dulbecco’s Modified Eagle Medium (DMEM) supplemented with 10% fetal bovine serum (FBS), 100 units/mL penicillin and 100 mg/mL streptomycin. H1299, LLC and HCC827 were cultured in 1640 medium supplemented with 10% fetal bovine serum (FBS), 100 units/mL penicillin and 100 mg/mL streptomycin. A549-luc cell line was cultured in EMEM medium supplemented with 10% fetal bovine serum (FBS), 1% non-essential amino acids, and 100 units/mL penicillin and 100 mg/mL streptomycin. H1299 or A549 were stimulated with TGF-β (5 ng/mL) for 24 h in the EMT assay. HCC827 was stimulated with TGF-β (5 ng/mL) and FGF-2 (5 ng/mL) for 24 h in the EMT assay. All cell lines were validated by STR profiling and tested negative for mycoplasma. Cells were all cultured in a humidified incubator at 37 °C and 5% CO_2_.

### Reagents and materials

The primary antibodies were obtained as follows. The antibodies Phospho-AMPKα (Thr172) (40H9) (2535), AMPKα (2532), AMPKα2 (2757), AMPKβ2 (4148), Phospho-Acetyl-CoA Carboxylase (Ser79) (3661), Acetyl-CoA Carboxylase (3662), E-cadherin (3195), N-cadherin (13116), PHF2 (34872), snail (3879), H3 (4499), GAPDH (2118), DYKDDDDK Tag (9A3) (8146) for immunofluorescence, Myc-Tag (9B11) (2276) were purchased from Cell Signalling Technology. The H3K9me2 (A2359), H3K9me3 (A2360), Fibronectin (A12977) for immunoblot or immunofluorescence were purchased from Abclonal Biotechnology. The PHF2 (HPA010831) antibody for immunohistochemistry were bought from Sigma-Aldrich. The H3K9me1 (PTM-614), H3K27me1 (PTM-620), H3K27me2 (PTM-621), H3K27me3 (PTM-622) for immunoblot were bought from PTM BioLab, Inc. The DYKDDDDK Tag (1E6) (018-22783) for immunoprecipitation and immunoblot was purchased from Wako. The antibody β-actin (AM1021B) was bought from Abgent.

The secondary antibodies were obtained as follows. For immunofluorescence assay, Alexa Fluor 555 labelled goat anti-mouse IgG (A21422), Alexa Fluor 555 labelled donkey anti-rabbit IgG (A31572) were purchased from Invitrogen. For immunoblot, peroxidase AffiniPure Goat Anti-Mouse IgG (H + L) (115-035-003) and Peroxidase AffiniPure Rabbit Anti-Goat IgG (H + L) (305-035-003) were purchased from Jackson ImmunoResearch Laboratories. Chemiluminescent detection was completed with chemiluminescent (ECL) western blotting reagents (GE healthcare, RPN2236). The p-PHF2 (Ser655) antibody was produced by Youke Biological Technology Co. Ltd (Shanghai, China).

The AMPK activator AICAR (HY-13417) and Metformin hydrochloride (HY-17471A) were purchased from MedChemExpress. A-769662 (CSN12424) was bought from CNSpharm. TGF-β (10804-HNAC) were purchased from SinoBiological. FGF-2 (MB2458-1) was bought from meilunbio. The siRNA was synthesised in Genepharma (Shanghai) and listed in Table S3.

### Construction of plasmids and generation of stable cell lines

To overexpress PHF2, the CDS sequence of human PHF2 was separately cloned into pcDNA3.1 plasmid, PEGFP-C2 plasmid and pCDH-CMV-MCS-EF1-GFP-puro plasmid. Consistently, human PHF2 plasmids were mutated into S655A or S655E. Similarly, the CDS sequence of human AMPKα1 and AMPKα2 were cloned into pcDNA3.1 plasmid and CDH1 was cloned into pCDH-CMV-MCS-EF1-GFP-puro vector. To obtain viral particles, 2 μg of lentiviral construct, 1.2 μg of pSPAX2, and 0.8 μg of VSV-G were co-transfected into HEK293T cells in 6 cm dish using Lipofectamine 3000 (Thermo). To knockdown PHF2, shRNA constructs were cloned into pLKO.1 plasmid. To obtain the shRNA viral particles, 2 μg of lentiviral construct, 1.5 μg of pSPAX2, and 0.5 μg of pMD2.G were co-transfected into HEK293T cells in 6 cm dish. About 48 h and 72 h after transfection, the virus supernatants were separately collected and filtered through 0.45 μm membrane (Millipore). The infected cells were selected with 2 μg/mL puromycin (meilunbio, MA0318). The 7TFP CDH1 reporter plasmid (91704) or the mutant (91703) was bought from addgene.

To knockout PHF2, sgRNA targeting PHF2 was inserted into pX330-U6-Chimeric_BB-CBh-hSpCas9 vectors. Then this plasmid and another GFP encoding plasmid were co-transfected into HEK293T cells for 24 h. GFP-high cells were sorted by FACS and seeded into 96-well plates to obtain single clone. The sequences of shRNA or sgRNA used in this study were listed in Table S3.

### Immunofluorescence

Cells used in the experiment were seeded on glass slides in 24-well plates in advance and fixed with 4% paraformaldehyde for 15 min followed with the permeabilization by 0.5% Triton X-100 for another 15 min. Next, cells were blocked with 5% BSA solution at room temperature for one additional hour, and then incubated with the primary antibody at 4 °C overnight. The next day, cells were washed with PBS for 3 times and incubated with diluted Alexa Fluor IgG secondary antibody at room temperature for 1 hour. Then, the nucleuses were stained with Hochest33422 (Invitrogen) at room temperature for 10 min. The final samples were imaged using the Opera High Content Imaging System (Perkinelmer, US) or Olympus FV1000 confocal microscopy.

#### Immunohistochemistry (IHC)

The tissues were paraffin embedded after overnight fixation with 4% paraformaldehyde solution. After dewaxing, sections were heated at 95 °C in pH 6.0 citric acid solution to extract epitopes. Then the endogenous peroxidase was quenched with 3% H2O2, followed by blocked with 5% BSA (Sigma-Aldrich, B2064-10G) in PBS for 1 h. Then the sections were incubated with indicated primary antibodies at 4 °C overnight. The next day, the sections were washed by PBS and incubated with the second antibodies for 1 h at room temperature and then stained with 3,3′-diaminobenzi- dine tetrahydrochloride for 10 min at room temperature. PerkinElmer Vectra®3 and Image J were applied for imaging and quantification, respectively.

### Immunoblot

Protein samples were harvested by SDS loading and denatured by boiling for 10 min. Then the samples were separated based on size using 8%~15% SDS-PAGE (SDS-polyacrylamide gel electrophoresis). The transfer time was determined according to the size of the target protein, and then the interested proteins were transferred to the PVDF membrane. The PVDF membrane was blocked with 5% skim milk or BSA at room temperature for 1 h. Wash the PVDF membrane three times with TBST and the membrane was incubated with corresponding primary antibody at 4 °C overnight. The next day, wash the primary antibody with TBST three times and the membrane was incubated with secondary antibody for 1 h. Finally, the membranes were photographed by the ChemiDoc system after being stained with ECL detection buffer (Bio-Rad, 1705061).

### Recombinant protein purification

In brief, the indicated plasmids were introduced into Escherichia coli BL21 cells and cultured in LB medium. The medium was rotated at a 37 °C, 250 rpm table concentrator, until the OD600 of the medium reached 0.4 to 0.6. Then 100 μM IPTG was added to the medium to induce protein expression at 16 °C for 16 h. Then the bacterial medium was harvested by centrifuging and resuspended in buffer A (150 mM NaCl, 50 mM Tris-HCl (pH7.4), 20 mM imidazole, 1 mM β-merhydryl ethanol, 5% glycerol, and 0.01%Triton X-100) followed by ultrasound for indicate time and power. The supernatant was then collected by centrifugation for multiple times to remove pellets and added to Ni Sepharose 6 Fast Flow (Yelibio.Company, 100012) for incubation at 4 °C for 2 h with gentle rotation. The globin mixture was harvested in the separation tube and washed with buffer B (150 mM NaCl, 50 mM Tris-HCl (pH7.4), 50 mM imidazole, 1 mM β-mercaptoethanol and 5% glycerol) to wash out the impurities. The protein was then eluted in buffer C (150 mm NaCl, 50 mM Tris-HCl (pH7.4), 200 mM imidazole, 1 mM β-mercaptoethanol, and 5% glycerol). Afterwards, the collected proteins were dialyzed overnight in buffer solution D (150 mM NaCl, 50 mM Tris-HCl (pH7.4), 1 mM EDTA (pH8.0), 1 mL β-mertoethanol and 10% glycerol) at 4 °C. Keep proteins at −80 °C for storage before use.

### Quantitative real-time PCR analyses

Total RNA of the samples was extracted according to the instructions of Trizol reagent (Invitrogen, 15596018). The RNA quality of each sample was detected by 1% agarose gel electrophoresis, and then the concentration and purity of RNA were detected by NanoDrop 2000. The prime script RT Master Mix (Abclonal, RK20402) was applied to reverse 1 μg total RNA into cDNA. According to the manufacturer’s instructions, real-time qPCR was carried out on the QuantStudio5 (Applied biosystems) using the SYBR GREEN QPCR KIT (Abclonal, RK21203). The normalization control for relative quantification of the data was either GAPDH or β-actin. All reactions were performed in triplicates. Primers were listed in Table S4.

### Co-immunoprecipitation

Western and IP cell lysis buffer (Beyotime, P0013) supplemented with a protease inhibitor cocktail (MCE, HY-K0010) was employed to lyse the cells. Whole cell lysates were harvested by centrifugation at 10,000 rpm at 4 °C for 10 min. After centrifugation, the precipitate was discarded and the supernatant was collected and IgG controls and indicated antibodies were added to the supernatant. Slowly rotate the mixture at 4 °C overnight. The next day, protein A agarose (Sigma Aldrich, P3476) was added to the mixture and incubated for another 2 h at 4 °C. Washed the mixture at 1200 rpm for 10 min. The supernatant was discarded and the precipitates (bead-antibody-protein complex) were washed at 1200 rpm, 4 °C five times with ice-cold PBS buffer. Precipitation was harvested by SDS-loading and detected by Western blot All operations were performed at 4 °C.

### Statistical analysis

Data were analyzed with two tailed unpaired Student’s *t*-test or one-way ANOVA or two-way ANOVA followed by fisher’s LSD tests with two tailed distribution using GraphPad Prism software. All results in this study are presented as mean ± SEM. Statistical significance was determined at *p < 0.05, **p < 0.01, ***p < 0.001.

## Supplementary information

Sigtrans_Supplementary_Materials

## Data Availability

The data that support the findings of this study are available from the corresponding author upon reasonable request.
