# Supplementary material for: Phosphorylation of PHF2 by AMPK releases the repressive H3K9me2 and inhibits cancer metastasis
Source: Signal Transduct Target Ther. 2023 Mar 6;8:95. doi: 10.1038/s41392-022-01302-6 (PMC9986243; doi:10.1038/s41392-022-01302-6)
Supplement: Supplementary file 1 — Sigtrans_Supplementary_Materials [file 41392_2022_1302_MOESM1_ESM.docx]

Supplementary Materials for

**Phosphorylation of PHF2 by AMPK releases the repressive H3K9me2 and inhibits cancer metastasis**

Ying Dong^1,2^, Hao Hu^1^, Xuan Zhang^1,2^, Yunkai Zhang^1,2^, Xin Sun^1,8^, Hanlin Wang^1,5^, Weijuan Kan^1^, Min-jia Tan^1,2^, Hong Shi^*6^, Yi Zang^*1,3,7^, Jia Li^*1,2,4,7,9^

Correspondence to:

Hong Shi (ada-shi@139.com)

Yi Zang (yzang@lglab.ac.cn)

Jia Li (jli@simm.ac.cn)

**This PDF file includes:**

Materials and Methods

Figures. S1 to S8

Original Data of Western Blots

Materials and Methods

**In vivo tumor growth and metastasis assay**

For tumor metastasis assay, A549 cells with PHF2 knockout (1×10^6^ cells/mice, 35 days) or PHF2-Mock/WT/S655A over expression (1×10^6^ cells/mice, 45 days) or PHF2-WT/S655E group (2×10^6^ cells/mice, 60 days) stable cell lines were injected into 6-week female balb/c nude mice via tail vein. Mice were sacrificed at indicated time and lung tissues were analyzed for the metastasis.

Another orthotopic transplantation mice model was performed in C57BL/6 mice. Briefly, Lewis lung carcinoma (LLC) cells of the control/metformin group (1×10^6^ cells/mice) or the scramble/shphf2 (5×10^5^ cells/mice) group were resuspended in 50 μL medium containing 25 μL matrigel and injected into the left lung lobe between the 4th and 5th ribs of C57BL/6 mice, followed with metformin (250 mg/kg) treatment orally for 15 days.

For assaying tumor growth in the xenograft model, 6-week-old female balb/c nude mice s were injected subcutaneously with 5×10^6^ A549 derivatives mixed with PBS and Matrigel (1:1, vol/vol). Tumor size was measured twice a week with a caliper, and the tumor volume was quatified by the formula: V = Length × Width × Width)/2. 35-40 days later, mice were sacrificed and xenografted solid tumors were dissected. All experimental procedures strictly complied with the IACUC guidelines.

**In vivo bioluminescence imaging.**

For visualizing the cancer metastasis, indicated A549-luc cells were injected intravenously. For imaging, mice were intraperitoneal injected with 100 mg/kg of sterile firefly D-luciferin (yeasen, 40901ES03). After 15 min, mice were placed in a gas anesthesia system (caliper life sciences, XGI-8) equipped with inhalation anesthesia. IVIS (in vivo imaging system) was used for imaging and analysis.

**In vitro kinase assay**

In brief, recombinant AMPKα2β2γ2 complex (400 nM) was fully activated by pre-incubation with CAMKKβ (40 nM) in kinase buffer at 37℃ water bath for 2 h. His-PHF2-WT or S655A mutant (400 nM) purified from bacteria were then incubated with activated AMPK (40 nM) in kinase buffer at 37℃ water bath for 3 h, where 5μCi[32P] -labeled ATP (Perkin Elmer, BLU002250UC) was included. The reaction was stopped by SDS loading buffer, followed by gel electrophoresis and subsequent autoradiography.

**Chromatin immunoprecipitation.**

Chromatin immunoprecipitation (ChIP)-qPCR was performed according to the protocol of SimpleChIP Enzymatic Chromatin IP Kit (CST, 9003s). In brief, cells were cross-linked with 1% formaldehyde (CST, 12606S), and chromatin DNA was cut to an average size of 300-500 bp by ultrasonic treatment. IgG or specific antibody were applied for immunoprecipitation overnight at 4 ℃, and then incubated with CHIP grade protein G magnetic beads for 2 h the next day. After washing and elution, the protein-DNA complex was reversed by heating overnight at 65 ℃. The immunoprecipitated DNA was purified with DNA purification column, and qPCR analysis was performed with the biosystem QuantStudio5. The primers used are specific to the tested region, and their sequences are listed in Table S5. All enrichment changes are normalized to input. The methylation of H3K9 is also normalized to total histone H3. GAPDH is used for non-specific binding sites.

**In vitro lysine demethylation assay**

The in vitro histone demethylation assays were carried out as previously described^33, 55^. For preparation of demethylases from mammalian cells, 293T cells were transfected with Flag-PHF2-WT/S655A/S655E plasmids using Lipofectamine 3000 (Invitrogen). After 48 h, the cells were lysed with Cell lysis buffer for Western and IP (Beyotime, P0013) supplemented with protease inhibitor cocktail (MCE, HY-K0010). For phosphorylation of PHF2 in vivo, cells were treated with metformin (1 mM) 24 or 48 h before harvest. Cell lysates were subjected to immunoprecipitation using anti-DYKDDDDK affinity beads (Smart-lifesciences, SA042001, China) and the target protein was eluted with 3*Flag peptides (Smart-lifesciences, SLR01001, China). The prepared PHF2 protein was used immediately for the demethylation reaction or stored in the -80℃.

For demethylase reaction, 2 µg of PHF2 protein was mixed with substrates: calf thymus histone (10 µg) (Sigma, H9250) or recombinant mono-nucleosomes (Active Motif, 81272) in the reaction buffer (20 mM Tris-HCl at pH 7.5, 150 mM KCl, 50 µM Fe(NH4)2(SO4)2–6H2O, 1 mM α-ketoglutarate, 1 mM ascorbate, 20 µM ZnCl2). The mixtures were incubated at 37 °C for 12 h, terminated in SDS-loading. The intensity of western blots was quantified using Image J.

**Preparation of nuclear fractions**

Cells were collected in PBS and pelleted at 4 ° C at 1000 rpm. The nuclear part was prepared according to the procedures of the Nuclear and Cytoplasmic Protein Extraction Kit (P0027, Beyotime, China). In brief, 200 µL of cytoplasmic protein extraction reagent A was added to every 20 µL of cell precipitation, whirled at maximum speed for 5 s, and ice bath for 10-15 min. Add 10 µL cytoplasmic protein extraction reagent B, swirl for 5 s at maximum speed, and take ice bath for 1 min. The supernatant of the extracted cytoplasmic protein was obtained by centrifugation. Then add 50 µL nuclear protein extraction reagent, and whirl for 15 s at the maximum speed every 1-2 min for a total of 30 min. Finally, centrifuge and harvest the extracted nucleoprotein supernatant.

**Histone peptide pull-down assay**

Biotinylated histone peptides (Millipore and Abgent), either modified or unmodified, were pre-incubated with Dynabeads streptavidin (Smart-lifesciences, SM01705, China) blocked with BSA at room temperature for 1 h in advance and then washed with washing buffer (0.01% Tween-20 in PBS) twice before mixing with cell nuclear exacts expressed protein. After overnight incubation at 4°C in the buffer contained 20 mM HEPES pH8.0, 1.5 mM MgCl2, 0.15 M NaCl, 25% glycerol, 1 mM DTT, 0.2 Mm EDTA, 0.01% Tween-20, pull-downs were washed with washing buffer (same as incubation buffer) five times followed by immunoblotting.

**α-KG detection assay**

The cellular α-KG detection was performed according to the protocol of a-Ketoglutarate Assay Kit (Sigma-Aldrich, MAK054). Briefly, cells (2 ×10^6^) can be homogenized in 100 µL ice cold a-KG Buffer. Centrifuge the samples at 13,000 rpm for 10 min to remove insoluble material. Take the final volume of samples to 50 µL with the a-KG assay buffer. Add 50 µL of reaction mix to each of the blank, standard, and test wells appropriately. Mix well using a horizontal shaker and incubate the reaction for 30 min at 37 °C. For colorimetric assays, measure the absorbance at 570 nm (A570).

**Transwell assay**

For migration and invasion assay, 24-well cell culture insert (Corning, 3422) and 24-well Matrigel invasion chamber (Corning, 354480) were applied. Cells suspended in serum-free medium (50,000 cells) were inoculated into the top chamber and the serum-containing medium (10% FBS) was added to bottom chamber. For migration assay, H1299, H441 and PC-9 were cultured for 24 h, A549 and LLC for 16 h unless otherwise specified. For invasion assay, H1299 and A549 were cultured for 24 h unless otherwise specified. The cells transferred to the bottom of the membrane were fixed with 4% formaldehyde, stained with 0.05% crystal violet at the specified time, and counted by Image J.

**Wound healing assay**

The A549 or H1299 was seeded in fusion state, and the cell monolayer was scratched with 10 µL pipette tip followed with twice wash using fresh medium. After replacing the serum free medium for 48 h, the migration area in the gap was measured under the light microscope.

Figure. S1.


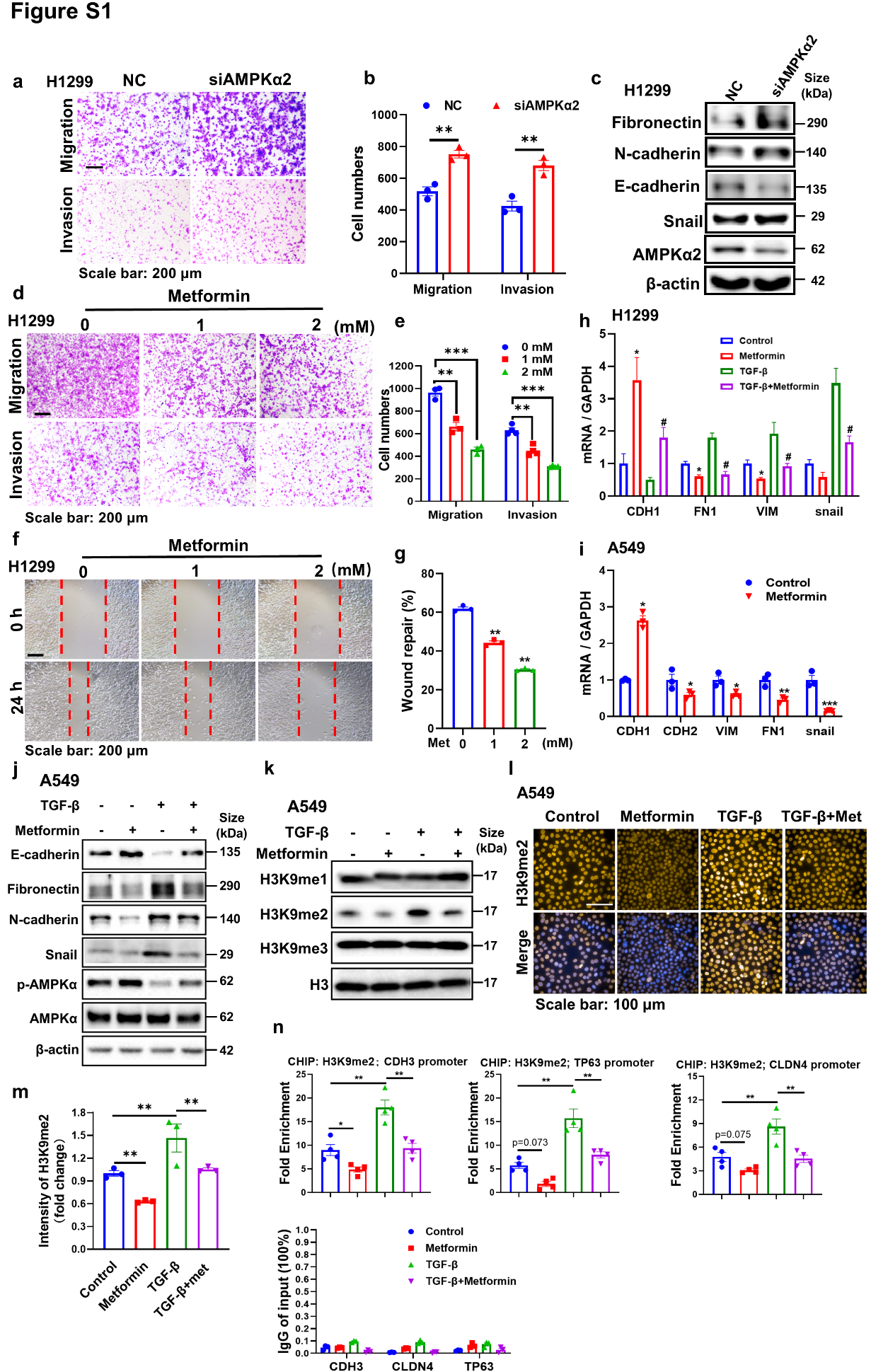


[**Supplement**](javascript:;)**ary Fig. 1** AMPK activation releases the repressive H3K9me2 on the promoters of epithelial genes and inhibits lung cancer metastasis

**a**-**b** Representative images and quantitative results of the migration and invasion in AMPK α2 knock-down H1299.

**c** Western blot analysis of WCLs derived from H1299 with siAMPKα2 treatment. β-actin served as loading control.

**d-e** Representative images and quantitative results of the migration and invasion in metformin treated H1299 cells with transwell assay.

**f**-**g** Representative images and quantitative results of the wound healing assay in metformin treated H1299.

**h-i** Quantitative real-time PCR analysis of various EMT markers and transcription factors in H1299 and A549 cells normalized to GAPDH.

**j** Western blot analysis of whole-cell lysates (WCLs) derived from A549 stimulated by TGF-β, combining with metformin treatment for 24 h. β-actin served as loading control.

**k** Western blot analysis of WCLs derived from A549 stimulated by TGF-β, combining with metformin treatment for 72 h. H3 served as loading control.

**l-m** Representative immunostaining images and quantitative results of the H3K9me2 intensity in metformin treated A549 cells for 72 h.

**n** CHIP-assay evaluating the recruitment of H3K9me2 to the CDH3, CLDN4, TP63 promoters after metformin or TGF-β treatment in H1299 for 24 h with several primers around transcriptional start site (TSS). The expression level of each IgG group of input (%) was less than 0.1%.

All error bars represent mean ± SEM. Statistical analyses were made using one-way ANOVA (g, m, n) or two-way ANOVA (b, e, h, i) followed by multiple comparisons of fisher’s LSD tests with two tailed distribution. Statistical significance was determined at p < 0.05(*); p < 0.01(**); p < 0.001(***).

Figure. S2.


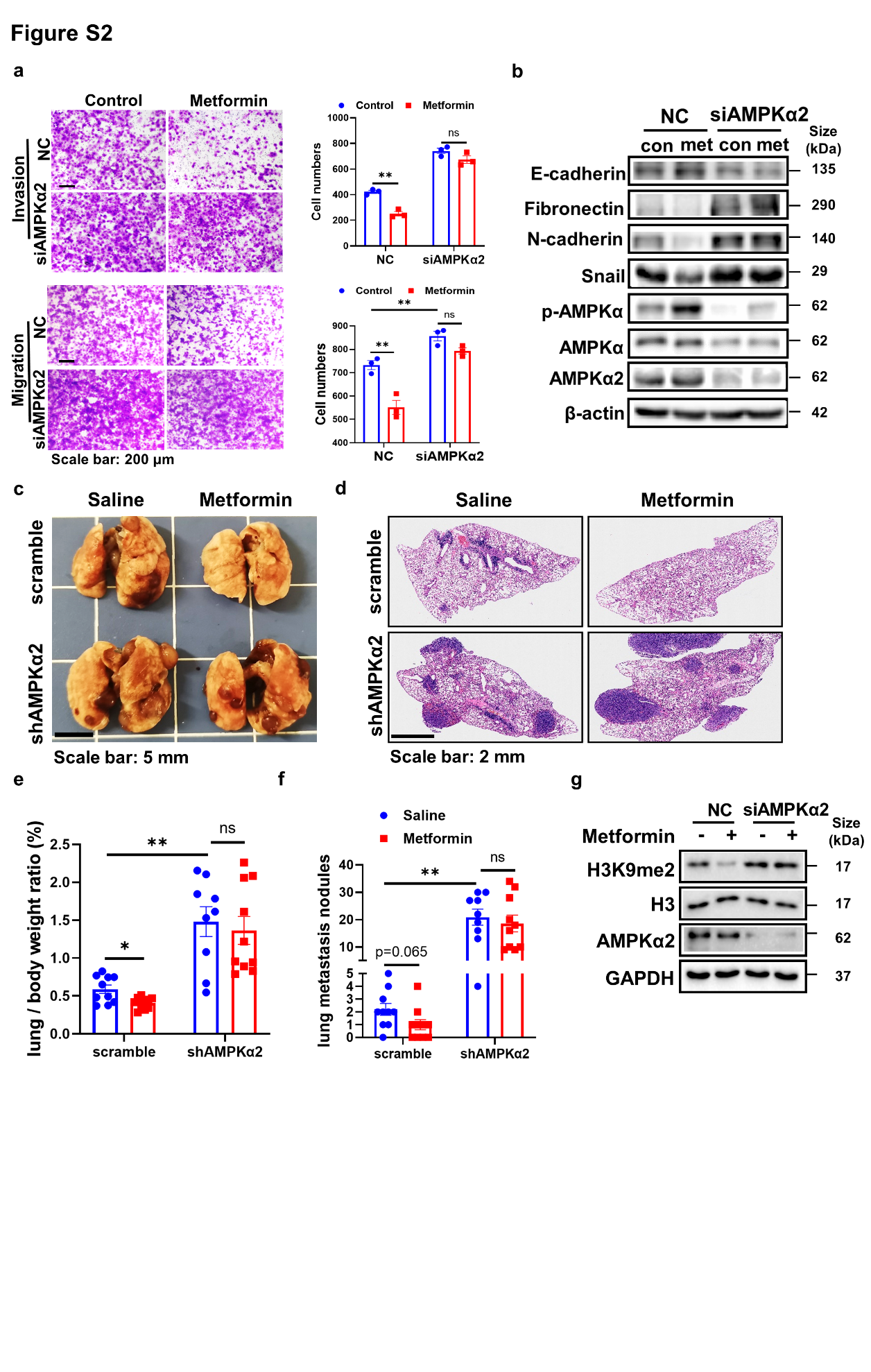


[**Supplement**](javascript:;)**ary Fig. 2** AMPKα2 deficiency abolishes the anti-metastasis and H3K9me2 downregulation effect of metformin

**a** Representative images and quantitative results of the invasive and migrative phenotype of H1299 transfected with siAMPKα2 followed by metformin treatment.

**b** Western blot analysis of WCLs derived from NC or siAMPKα2 transfected H1299 cells followed with metformin treatment for 24 h. β-actin served as loading control.

**c** Representative images of lung nodules of C57BL/6 mice acquired 21 days after orthotopic transplantation of the scramble and shAMPKα2 stable cell lines LLC followed with metformin treatment via oral every day.

**d** Representative images of H&E stained histological sections of lungs from C57BL/6.

**e**-**f** Quantitative results of lung/body weight ratio and lung nodules from corresponding mice (n=9-10 each group).

**g** Western blot analysis of WCLs derived from NC or siAMPKα2 transfected H1299 cells followed with metformin treatment for 72 h. H3 served as loading control.

All error bars represent mean ± SEM. Statistical analyses were made using two-way ANOVA (**a**, **e**, **f**) followed by multiple comparisons of fisher’s LSD tests with two tailed distribution. Statistical significance was determined at p < 0.05(*); p < 0.01(**); p < 0.001(***).

Figure. S3.


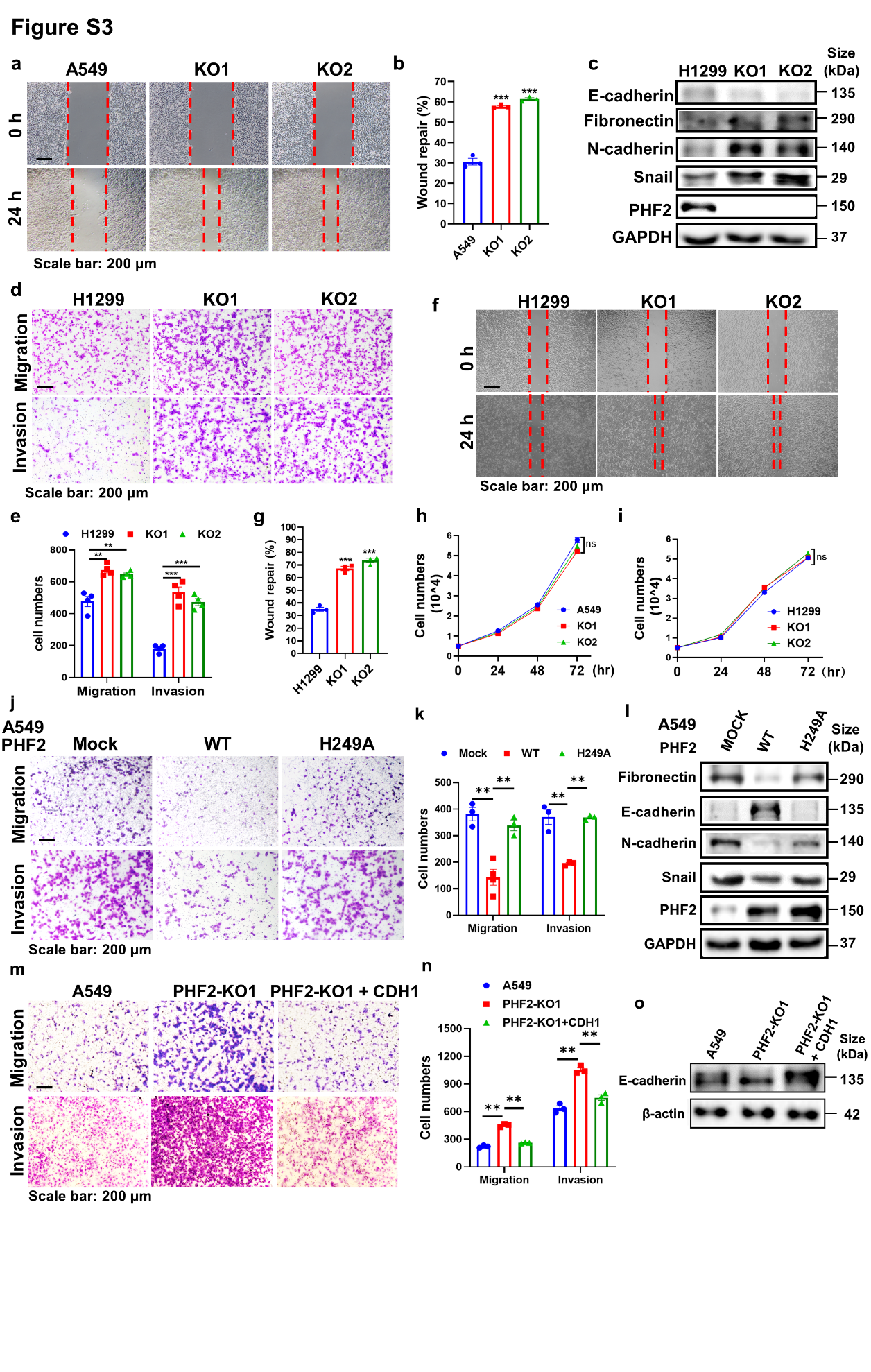


[**Supplement**](javascript:;)**ary Fig. 3** PHF2 deficiency enhances lung cancer metastasis in vitro mostly due to E-cadherin repression

**a**-**b** Representative images and quantitative results of the wound healing assay of A549 and PHF2 knockout cells.

**c** Western blot analysis of whole-cell lysates (WCLs) derived from H1299 and PHF2 knockout cells. GAPDH served as loading control.

**d**-**e** Representative images and quantitative results of the migration and invasion of H1299 and PHF2 knockout cells.

**f**-**g** Representative images and quantitative results of the wound healing assay of H1299 and PHF2 knockout cells.

**h** Cell proliferation curve of A549 and PHF2 knockout cells, KO1 and KO2.

**i** Cell proliferation curve of H1299 and PHF2 knockout cells, KO1 and KO2.

**j**-**k** Representative images and quantitative results of the migration and invasion of stable cell lines of mock, PHF2-WT and H249A in H1299.

**l** Western blot analysis of WCLs derived from mock, PHF2-WT and H249A in H1299. GAPDH served as loading control.

**m**-**n** Representative images and quantitative results of the migration and invasion of A549, PHF2-KO and CDH1 overexpression cells.

**o** Validation of CDH1 overexpression in PHF2-KO A549 cells by immunoblot analysis.

All error bars represent mean ± SEM. Statistical analyses were made using one-way ANOVA (**b**, **g**) or two-way ANOVA (**e**, **k**, **n**) followed by multiple comparisons of fisher’s LSD tests with two tailed distribution. Statistical significance was determined at p < 0.05(*); p < 0.01(**); p < 0.001(***).

Figure. S4.


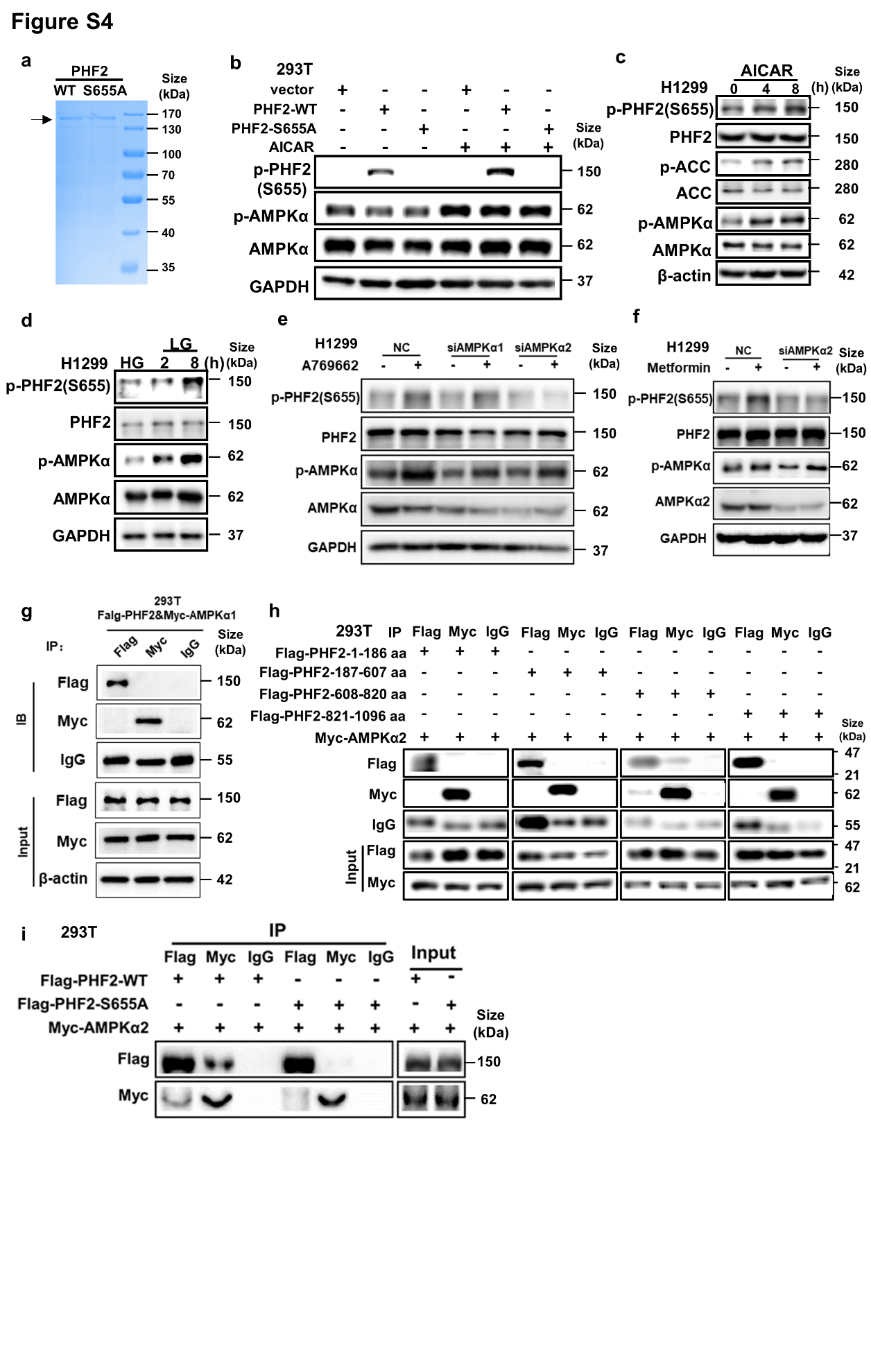


[**Supplement**](javascript:;)**ary Fig. 4** AMPK directly phosphorylates PHF2 at S655

**a** Bacterial expressed PHF2 and S655A recombinant proteins were subjected Coomassie blue staining.

**b** Immunoblot analysis of WCLs derived from 293T cells transfected with Flag-PHF2-WT or S655A for 48 h followed with 1 mM AICAR treatment. GAPDH served as loading control.

**c** H1299 cell treated with AICAR (1 mM at indicated time) were subjected for immunoblot analysis. β-actin served as loading control.

**d** Immunoblot analysis of WCLs derived from H1299 treated with cultured in different concentrations of glucose (HG: 25 mM, LG: 5 mM) for indicated time. GAPDH served as loading control.

**e** Immunoblot analysis of WCLs derived from NC, siAMPKα1 or siAMPKα2 treated H1299 followed by A769662 treatment for 4 h. GAPDH served as loading control.

**f** Immunoblot analysis of WCLs derived from NC or siAMPKα2 treated H1299 followed by metformin treatment for 24 h. GAPDH served as loading control.

**g** Immunoblot analysis of whole-cell lysates (input) and anti-Flag, anti-Myc or anti-IgG immunoprecipitates derived from 293T cells co-transfected with Flag-PHF2 and Myc-AMPKα1.

**h** Immunoblot analysis of WCLs and immunoprecipitates derived from 293T cells co-transfected with the indicated Myc-AMPKα2 and four truncations of Flag-PHF2 plasmids.

**i** Immunoblot analysis of whole-cell lysates (input) and anti-Flag, anti-Myc or anti-IgG immunoprecipitates derived from 293T cells co-transfected with Myc-AMPKα2 and Flag-PHF2-WT or S655A.

Figure. S5.


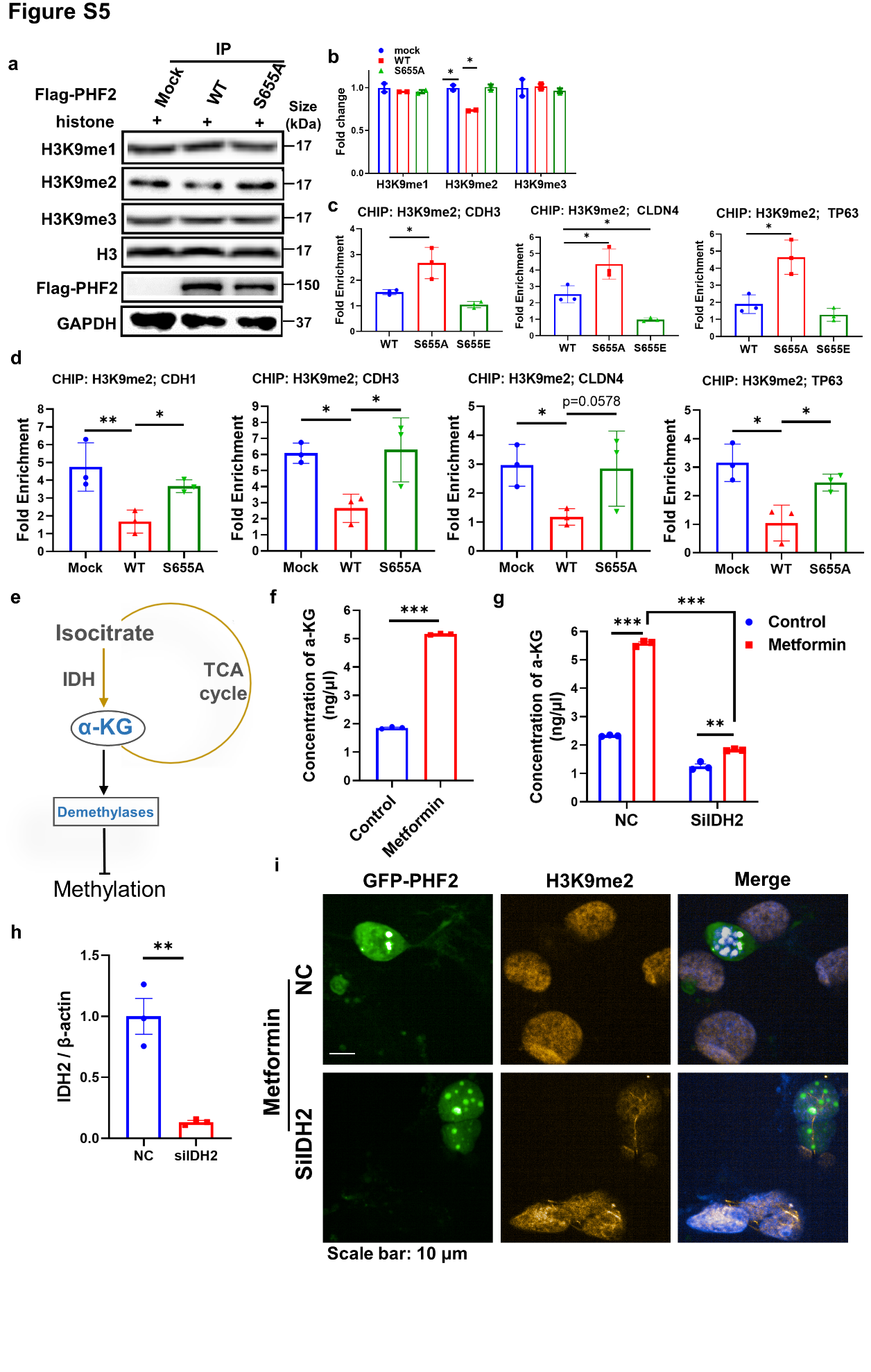


**[Supplement](javascript:;)ary Fig. 5** PHF2-S655 phosphorylation is critical for its demethylase activity and α-KG metabolites play a minor role

**a**-**b** Immunoblot analysis and quantitative results of the PHF2-WT or S655A immune-precipitate from H1299 incubated with histone for 6 h in 37℃ in the histone demethylation buffer. H3 served as loading control.

**c** CHIP-qPCR analysis of H3K9me2 marks in the promoter of CDH3, CLDN4, and TP63 in PHF2 - WT/S655A/S655E stable cell lines of H1299.

**d** CHIP-qPCR analysis of H3K9me2 marks in the promoter of epithelial genes including CDH1, CDH3, CLDN4, and TP63 in A549 stable cell lines (mock, PHF2-WT and PHF2-S655A).

**e** The schematic of α-KG metabolites function as substrates of demethylases.

**f** The α-KG concentration of control or metformin treated H1299 cells.

**g** The α-KG concentration of control or metformin treated H1299 cells with IDH2 deficiency.

**h** The knock-down efficiency of IDH2 in H1299 validated by the quantitative real-time PCR analysis.

**i** Immunostaining results of H3K9me2 and GFP-PHF2 expression levels in H1299 cells with IDH2 knock-down and metformin treatment.

All error bars represent mean ± SEM. Statistical analyses were made using unpaired t-test (**f, h**) or one-way ANOVA (**c**, **d**) or two-way ANOVA (**b**, **g**) followed by multiple comparisons of fisher’s LSD tests with two tailed distribution. Statistical significance was determined at p < 0.05(*); p < 0.01(**); p < 0.001(***).

Figure. S6.


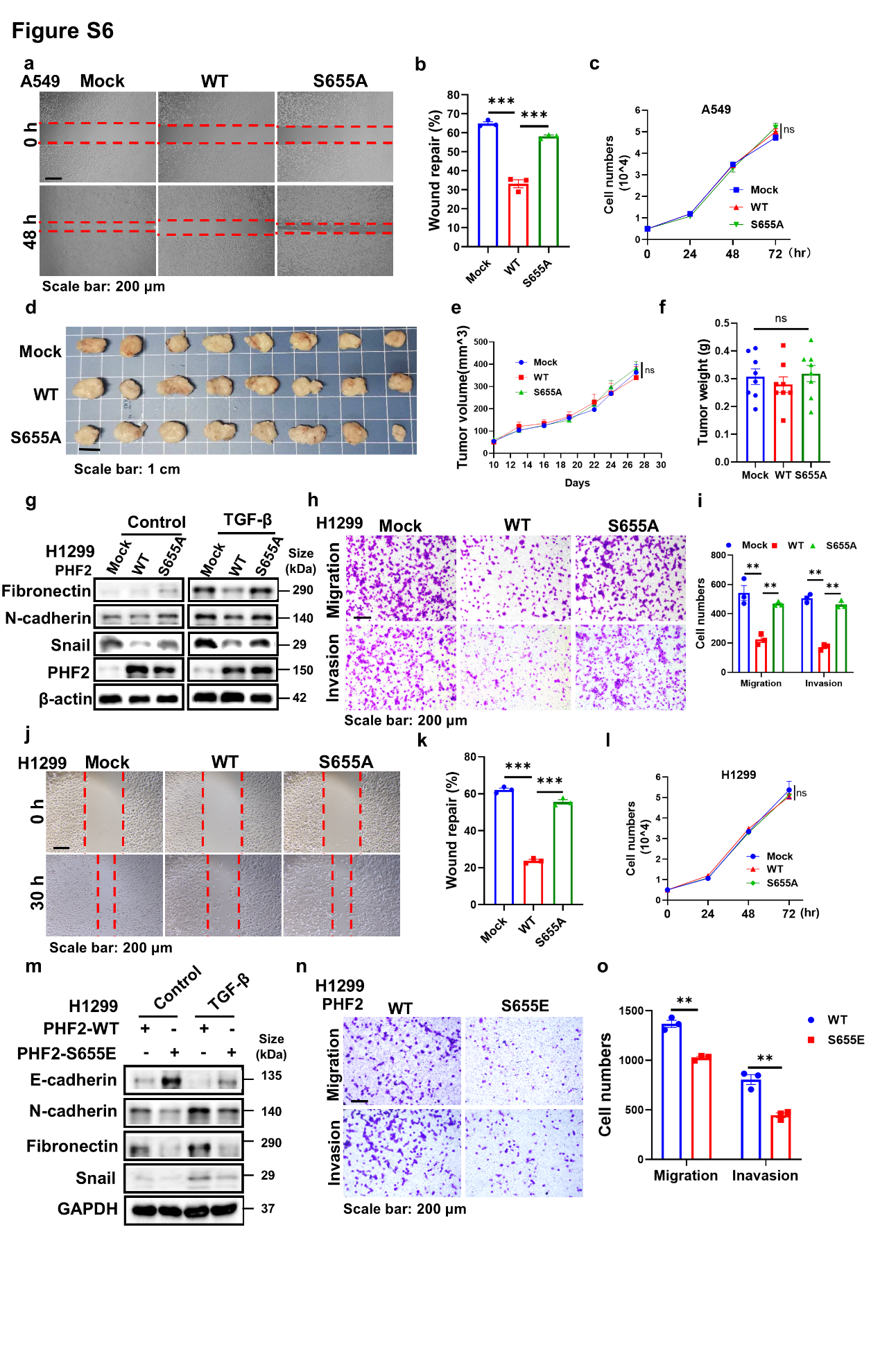


[**Supplement**](javascript:;)**ary Fig. 6** AMPK mediated PHF2-S655 phosphorylation suppresses lung cancer metastasis.

**a**-**b** Representative images and quantitative results of the wound healing assay in A549 stable cell lines (Mock, PHF2-WT and PHF2-S655A).

**c** Cell proliferation curve of Mock, PHF2-WT and PHF2-S655A stable cell lines in A549.

**d** Representative images of the xenograft lung tumor in Balb/c nude mice subcutaneously injected with Mock, PHF2-WT and PHF2-S655A stable cell lines in A549.

**e**-**f** Quantitative results of the tumor volume and tumor weight from corresponding mice in [Supplement](javascript:;)ary Fig. 6d.

**g** Western blot analysis of WCLs derived from mock, PHF2-WT and PHF2-S655A stable cell lines in H1299. β-actin served as loading control.

**h**-**i** Representative images and quantitative results of the migration and invasion of mock, PHF2-WT and PHF2-S655A stable cell lines in H1299.

**j**-**k** Representative images and quantitative results of the wound healing assay in H1299 stable cell lines (Mock, PHF2-WT and PHF2-S655A).

**l** Cell proliferation curve of H1299 stable cell lines (Mock, PHF2-WT and PHF2-S655A).

**m** Western blot analysis of WCLs derived from PHF2-WT and S655E stable cell lines in H1299 stimulated with or without TGF-β1 for 24 h.

**n**-**o** Representative images and quantitative results of the migration (24 h) and invasion (36 h) of PHF2-WT and S655E stable cell lines in H1299.

All error bars represent mean ± SEM. Statistical analyses were made using one-way ANOVA (**b**, **c**, **e**, **f**, **k**) and two-way ANOVA (**i** and **o**) followed by fisher’s LSD tests with two tailed distribution. Statistical significance was determined at p < 0.05(*); p < 0.01(**); p < 0.001(***).

Figure. S7.


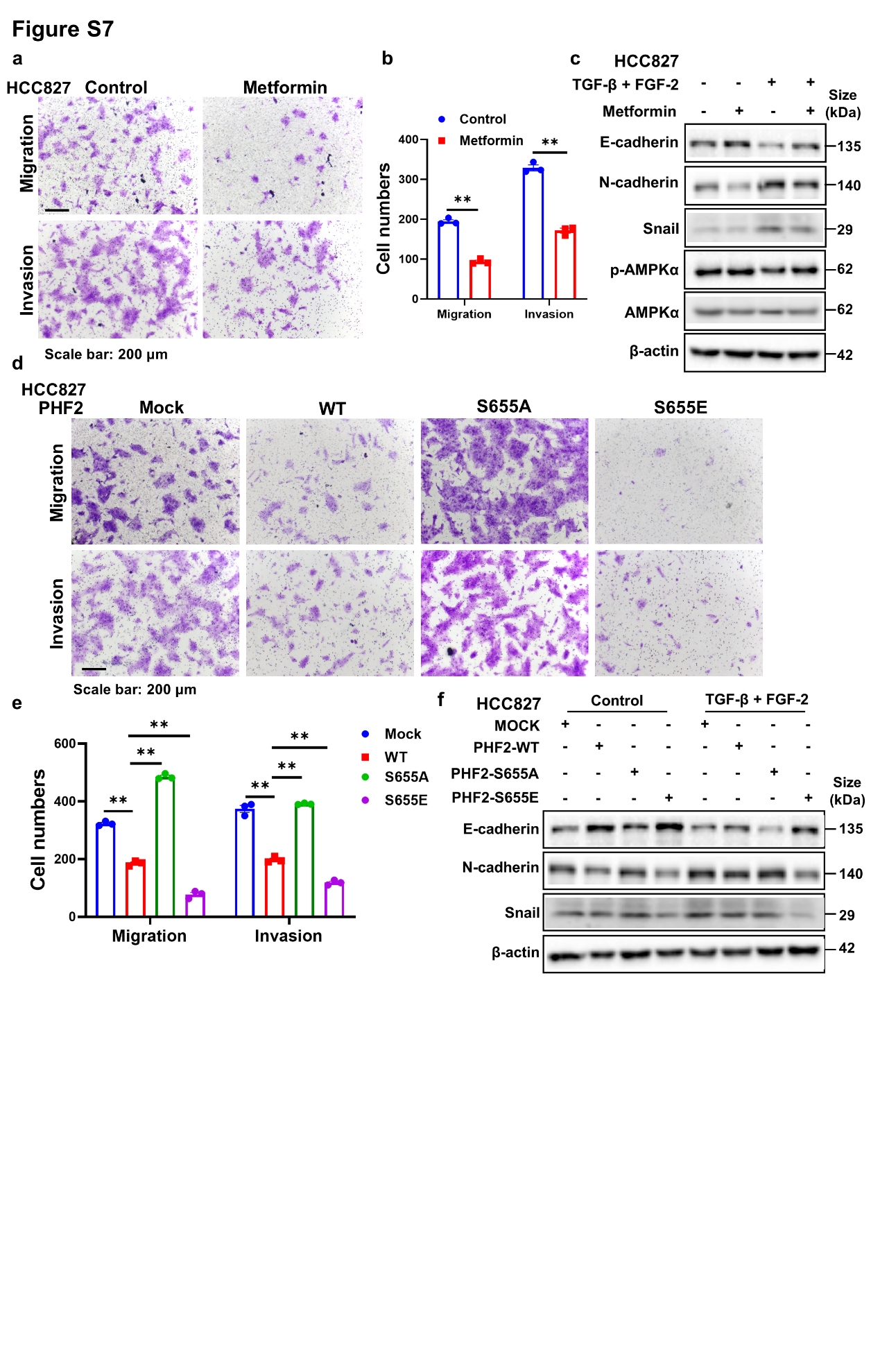


[**Supplement**](javascript:;)**ary Fig. 7** The Validation of AMPK-PHF2-EMT axis in EGFR-mutant cell line HCC827.

**a**-**b** Representative images and quantitative results of the migration and invasion of HCC827 cells treated with metformin for 24 h.

**c** Immunoblot analysis of EMT indicators from HCC827 cells treated with metformin or stimulated with TGF-β (5 ng/mL) and FGF-2 (5 ng/mL) for 24 h. β-actin served as loading control.

**d**-**e** Representative images and quantitative results of the migration and invasion of HCC827 cells overexpressed with mock, PHF2-WT, S655A, S655E.

**f**. Immunoblot analysis of EMT indicators of HCC827 cells overexpressed with mock, PHF2-WT, S655A, S655E stimulated with TGF-β (5 ng/mL) and FGF-2 (5 ng/mL) for 24 h. β-actin served as loading control. All error bars represent mean ± SEM. Statistical analyses were made using two-way ANOVA (**b** and **e**) followed by fisher’s LSD tests with two tailed distribution. Statistical significance was determined at p < 0.05(*); p < 0.01(**); p < 0.001(***).

Figure. S8.


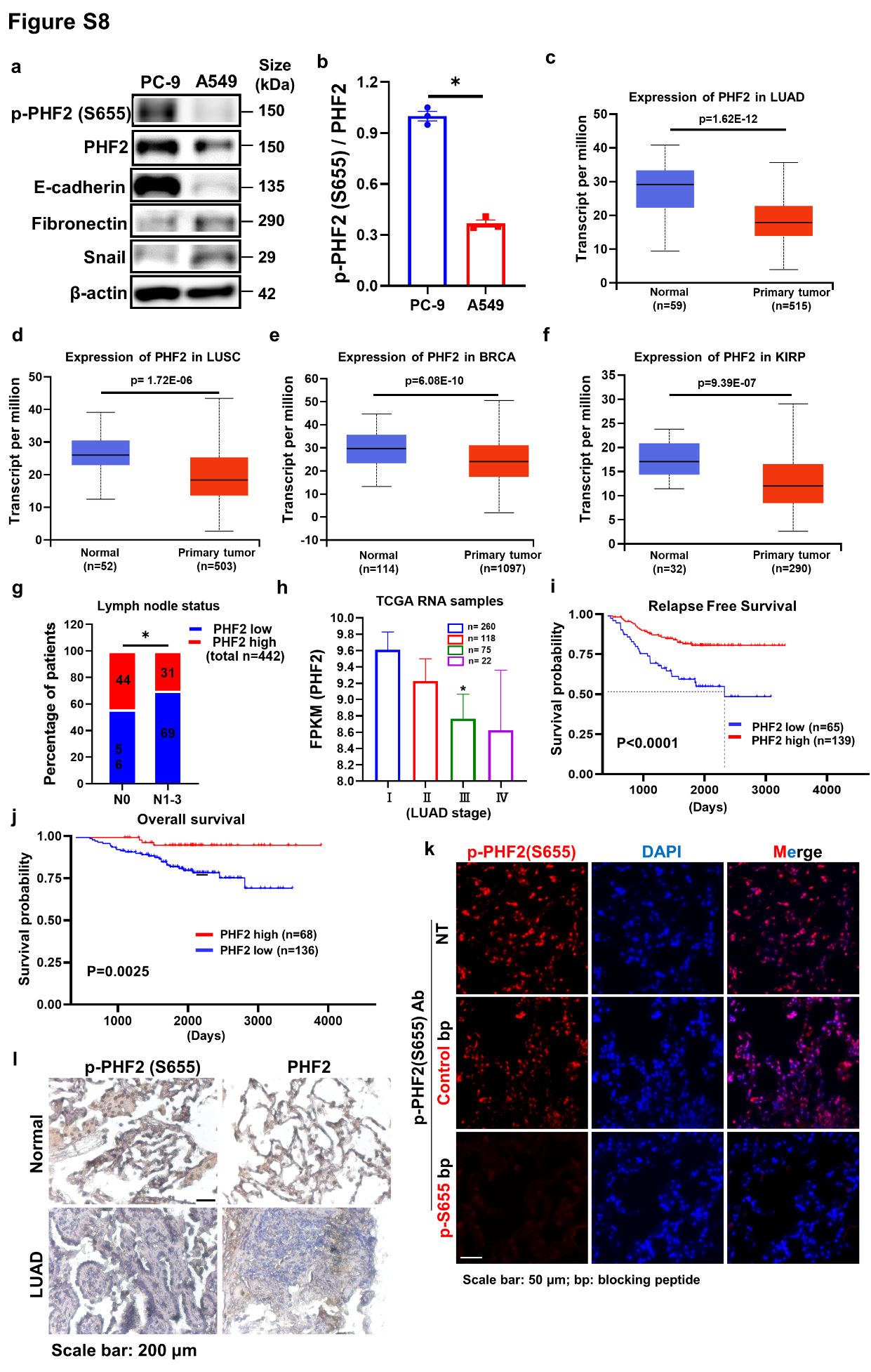


[**Supplement**](javascript:;)**ary** **Fig. 8** PHF2 expression is downregulated and positively associated with the prognosis in lung cancer patients.

**a**-**b** Western blot analysis of WCLs derived from PC-9 and A549. And the Quantitative results of p-PHF2(S655) relative with PHF2 expression with Image J. β-actin served as loading control.

**c**-**f** PHF2 expression analyses between normal and primary tumor tissues in lung squamous cell carcinoma (LUSC), lung adenocarcinoma (LUAD), breast cancer (BRCA) and Kidney renal papillary cell carcinoma (KIRP) patients of TCGA database from http://ualcan.path.uab.edu/analysis.html.

**g** Analyses of the correlation between PHF2 expression and lymph node metastasis from TCGA database.

**h** Analyses of PHF2 expression in various stages of LUAD patients from TCGA database.

**i** Kaplan-Meier plots of TCGA data showing relapse-free survival of LUAD patients with high and low PHF2 expression.

**j** Kaplan-Meier plots of TCGA data showing overall survival of LUAD patients with high and low PHF2 expression.

**k** Immunostaining results of p-PHF2(S655) in clinical lung tissues to confirm the antibody specificity. Anti-p-PHF2(S655) antibody was separately incubated with p-PHF2 (S655) blocking peptide and the relative control in advance to confirm that the antibody indeed works on FFPE tissue slides.

**l** Immunohistochemical staining results of p-PHF2(S655) in clinical LUAD samples. All error bars represent mean ± SEM. Statistical analyses were made using unpaired t-test (**b**-**g**) or log-rank test (**i** and **j**) or one-way ANOVA (**h**) followed by multiple comparisons of fisher’s LSD tests with two tailed distribution. Statistical significance was determined at p < 0.05(*); p < 0.01(**); p < 0.001(***).

Table S1. Clinicopathological characteristics of non-small cell lung cancer (NSCLC) patients from hospital

| **Case No.** | **Gender** | **Age** | **Cancer Subtype** | **Cancer Stage** |
| --- | --- | --- | --- | --- |
| #1 | Female | 67 | LUAD | T4N0M0 |
| #2 | Female | 49 | LUAD | T4N0M0 |
| #3 | Female | 69 | LUAD | T2N2M0 |
| #4 | Male | 63 | LUAD | T1N0M0 |
| #5 | Female | 64 | LUAD | T2N0M0 |
| #6 | Female | 61 | LUAD | T3N0M0 |
| #7 | Male | 69 | LUAD | T4N0M0 |
| #8 | Male | 71 | LUAD | T3N0M0 |
| #9 | Male | 71 | LUAD | T4N0M0 |
| #10 | Male | 75 | LUAD | T2N0M0 |

Table S2. Pathological characteristics of NSCLC patients of microarray

| **Histologic type** | **Gender** | **Age** | **Cancer Subtype** | **Grading** | **lymph node metastases** | **T** | **N** | **M** | **Staging** |
| --- | --- | --- | --- | --- | --- | --- | --- | --- | --- |
| carcinoma and adjacent tissue | female | 57 | LUAD | Ⅰ-Ⅱ |  |  |  | M0 |  |
| carcinoma and adjacent tissue | female | 63 | LUAD | Ⅱ |  |  |  | M0 |  |
| carcinoma and adjacent tissue | male | 51 | LUAD | Ⅱ |  |  |  | M0 |  |
| carcinoma and adjacent tissue | female | 55 | LUAD | Ⅱ |  |  |  | M0 |  |
| carcinoma and adjacent tissue | male | 53 | LUAD | Ⅱ |  |  |  | M0 |  |
| carcinoma and adjacent tissue | female | 73 | LUAD | Ⅱ | 18 |  | N1-3? | M0 |  |
| carcinoma and adjacent tissue | female | 56 | LUAD | Ⅱ | 18 | T2a | N0 | M0 | 1 |
| carcinoma and adjacent tissue | female | 73 | LUAD | Ⅱ | 14 |  | N0 | M0 |  |
| carcinoma and adjacent tissue | female | 60 | LUAD | Ⅲ | 7 | T2a | N1 | M0 | 2 |
| carcinoma and adjacent tissue | female | 68 | LUAD | Ⅱ-Ⅲ | 15 | T3 | N2 | M0 | 3 |
| carcinoma and adjacent tissue | male | 76 | LUAD | Ⅰ-Ⅱ | 10 | T2a | N0 | M0 | 1 |
| carcinoma and adjacent tissue | male | 65 | LUAD | Ⅱ | 10 | T1 | N0 | M0 | 1 |
| carcinoma and adjacent tissue | male | 72 | LUAD | Ⅱ | 20 | T2a | N2 | M0 | 3 |
| carcinoma and adjacent tissue | female | 53 | LUAD | Ⅰ-Ⅲ | 19 | T1 | N1 | M0 | 2 |
| carcinoma and adjacent tissue | male | 66 | LUAD | Ⅱ | 23 |  | N1-3? | M0 |  |
| carcinoma and adjacent tissue | female | 76 | LUAD | Ⅱ |  |  |  | M0 |  |
| carcinoma and adjacent tissue | male | 57 | LUAD | Ⅱ | 19 | T2a | N0 | M1 | 4 |
| carcinoma and adjacent tissue | male | 77 | LUAD | Ⅱ-Ⅲ | 10 | T1 | N2 | M0 | 3 |
| carcinoma and adjacent tissue | male | 52 | LUAD | Ⅱ | 4 | T2a | N0 | M0 | 1 |
| carcinoma and adjacent tissue | male | 69 | LUAD | Ⅲ |  | T4 | N0 | M0 | 3 |
| carcinoma and adjacent tissue | male | 59 | LUAD | Ⅱ-Ⅲ |  |  |  | M0 |  |
| carcinoma and adjacent tissue | male | 76 | LUAD | Ⅱ-Ⅲ |  | T2a | N0 | M0 | 1 |
| carcinoma and adjacent tissue | female | 71 | LUAD | Ⅱ | 23 | T2a | N0 | M0 | 1 |
| carcinoma and adjacent tissue | male | 53 | LUAD | Ⅲ | 13 | T4 |  | M0 | 3 |
| carcinoma and adjacent tissue | female | 68 | LUAD | Ⅱ-Ⅲ | 10 | T2a | N1 | M0 | 2 |
| carcinoma and adjacent tissue | male | 54 | LUAD | Ⅰ-Ⅱ | 2 | T2b | N0 | M0 | 2 |
| carcinoma and adjacent tissue | female | 61 | LUAD | Ⅱ | 21 | T1 | N0 | M0 | 1 |
| carcinoma and adjacent tissue | male | 57 | LUAD | Ⅱ-Ⅲ | 15 | T2a | N1-3? | M0 | 2 |
| carcinoma and adjacent tissue | male | 86 | LUAD | Ⅱ | 17 | T1 | N1 | M0 | 2 |
| carcinoma and adjacent tissue | male | 70 | LUAD | Ⅱ-Ⅲ | 18 | T3 | N0 | M0 | 2 |
| carcinoma and adjacent tissue | male | 73 | LUAD | Ⅰ-Ⅱ | 15 | T1 | N0 | M0 | 1 |
| carcinoma and adjacent tissue | female | 69 | LUAD | Ⅱ | 12 | T1 | N0 | M0 | 1 |
| carcinoma and adjacent tissue | female | 64 | LUAD | Ⅱ-Ⅲ |  |  |  | M0 |  |
| carcinoma and adjacent tissue | female | 56 | LUAD | Ⅱ | 35 | T2a | N2 | M0 | 3 |
| carcinoma and adjacent tissue | male | 54 | LUAD | Ⅱ-Ⅲ | 16 | T2a | N1-3? | M0 | 2 |
| carcinoma and adjacent tissue | female | 62 | LUAD | Ⅱ | 16 | T2a | N1 | M0 | 2 |
| carcinoma and adjacent tissue | female | 73 | LUAD | Ⅱ-Ⅲ |  | T2a |  | M0 |  |
| carcinoma and adjacent tissue | male | 73 | LUAD | Ⅱ | 14 | T2a | N0 | M0 | 1 |
| carcinoma and adjacent tissue | male | 52 | LUAD | Ⅱ-Ⅲ | 7 | T1 |  | M0 | 3 |
| carcinoma and adjacent tissue | female | 55 | LUAD | Ⅱ | 9 | T1 | N0 | M0 | 1 |
| carcinoma and adjacent tissue | male | 63 | LUAD | Ⅰ-Ⅱ | 12 | T2a | N0 | M0 | 1 |
| carcinoma and adjacent tissue | male | 53 | LUAD | Ⅱ | 14 | T1 | N2 | M0 | 3 |
| carcinoma and adjacent tissue | male | 49 | LUAD | Ⅱ | 29 | T3-4 | N1-3? | M0 | 3 |
| carcinoma and adjacent tissue | male | 60 | LUAD | Ⅲ | 1 | T2b | N1 | M0 | 2 |
| carcinoma and adjacent tissue | male | 54 | LUAD | Ⅰ-Ⅱ | 9 | T1 | N0 | M0 | 1 |
| carcinoma and adjacent tissue | male | 70 | LUAD | Ⅱ | 13 | T2a | N1-3? | M0 | 23 |
| carcinoma and adjacent tissue | male | 59 | LUAD | Ⅲ | 6 | T2a | N0 | M0 | 1 |
| carcinoma and adjacent tissue | male | 61 | LUAD | Ⅱ-Ⅲ | 15 | T2b | N1 | M0 | 2 |
| carcinoma and adjacent tissue | female | 66 | LUAD | Ⅱ-Ⅲ | 12 | T2a | N1 | M0 | 2 |
| carcinoma and adjacent tissue | female | 50 | LUAD | Ⅱ |  | T4 |  | M0 | 3 |
| carcinoma and adjacent tissue | male | 44 | LUAD | Ⅱ | 12 | T1 | N0 | M0 | 1 |
| carcinoma and adjacent tissue | female | 52 | LUAD | Ⅱ | 23 | T1 | N0 | M0 | 1 |
| carcinoma and adjacent tissue | male | 74 | LUAD | Ⅲ | 14 | T3 | N1-3? | M0 | 3 |
| carcinoma and adjacent tissue | male | 62 | LUAD | Ⅱ | 11 | T2a | N0 | M0 | 1 |
| carcinoma and adjacent tissue | male | 60 | LUAD | Ⅱ |  | T1 |  | M0 |  |
| carcinoma and adjacent tissue | female | 51 | LUAD | Ⅱ-Ⅲ | 14 | T2b | N1 | M0 | 2 |
| carcinoma and adjacent tissue | male | 79 | LUAD | Ⅱ | 2 | T2a | N0 | M0 | 1 |
| carcinoma and adjacent tissue | female | 62 | LUAD | Ⅱ | 14 | T4 | N1-3? | M0 | 3 |
| carcinoma and adjacent tissue | male | 63 | LUAD | Ⅱ | 22 | T1 | N1-3? | M0 | 23 |
| carcinoma and adjacent tissue | male | 75 | LUAD | Ⅰ-Ⅲ | 7 | T2a | N0 | M0 | 1 |
| carcinoma and adjacent tissue | male | 62 | LUAD | Ⅱ | 13 | T3 | N0 | M0 | 2 |
| carcinoma and adjacent tissue | female | 66 | LUAD | Ⅰ | 24 | T1 | N0 | M0 | 1 |
| carcinoma and adjacent tissue | female | 83 | LUAD | Ⅱ-Ⅲ | 8 | T2a | N0 | M0 | 1 |
| carcinoma and adjacent tissue | female | 75 | LUAD | Ⅱ | 4 | T3 | N0 | M0 | 2 |
| carcinoma and adjacent tissue | female | 60 | LUAD | Ⅰ-Ⅲ | 20 | T2a | N1 | M0 | 2 |
| carcinoma and adjacent tissue | female | 54 | LUAD | Ⅱ | 24 | T4 | N3 | M0 | 3 |
| carcinoma and adjacent tissue | male | 76 | LUAD | Ⅱ-Ⅲ |  | T3 |  | M0 |  |
| carcinoma and adjacent tissue | male | 65 | LUAD | Ⅱ |  | T4 |  | M0 | 3 |
| carcinoma and adjacent tissue | female | 69 | LUAD | Ⅱ-Ⅲ | 18 | T3 | N1 | M0 | 3 |
| carcinoma and adjacent tissue | male | 35 | LUAD | Ⅱ-Ⅲ | 2 | T2a | N1 | M0 | 2 |
| carcinoma and adjacent tissue | female | 68 | LUAD | Ⅱ | 20 | T2a | N0 | M0 | 1 |
| carcinoma and adjacent tissue | male | 60 | LUAD | Ⅱ-Ⅲ | 9 | T3 | N1-3? | M1 | 4 |
| carcinoma and adjacent tissue | female | 64 | LUAD | Ⅱ | 3 | T2a | N0 | M0 | 1 |
| carcinoma and adjacent tissue | female | 63 | LUAD | Ⅱ | 8 | T4 | N1-3? | M0 | 3 |
| carcinoma and adjacent tissue | male | 53 | LUAD | Ⅲ |  | T1 |  | M0 |  |
| carcinoma and adjacent tissue | male | 59 | LUAD | Ⅱ |  | T1 |  | M0 |  |
| carcinoma and adjacent tissue | female | 65 | LUAD | Ⅱ |  | T1 |  | M0 |  |
| carcinoma and adjacent tissue | female | 52 | LUAD | Ⅰ-Ⅲ |  | T1 |  | M0 |  |
| carcinoma and adjacent tissue | female | 78 | LUAD | Ⅱ |  | T2a |  | M0 |  |
| carcinoma and adjacent tissue | female | 80 | LUAD | Ⅱ | 17 | T2a | N0 | M0 | 1 |
| carcinoma and adjacent tissue | female | 57 | LUAD | Ⅱ | 18 | T2a | N2 | M0 | 3 |
| carcinoma and adjacent tissue | female | 44 | LUAD | Ⅱ-Ⅲ |  | T2a |  | M0 |  |
| carcinoma and adjacent tissue | male | 69 | LUAD | Ⅱ |  | T2a |  | M0 |  |
| carcinoma and adjacent tissue | female | 55 | LUAD | Ⅱ |  | T1 |  | M0 |  |
| carcinoma and adjacent tissue | female | 40 | LUAD | Ⅱ | 20 | T3 | N1-3? | M0 | 3 |
| carcinoma and adjacent tissue | male | 67 | LUAD | Ⅱ-Ⅲ |  | T1 |  | M0 |  |
| carcinoma and adjacent tissue | male | 65 | LUAD | Ⅱ-Ⅲ | 24 | T3 |  | M0 | 3 |
| carcinoma and adjacent tissue | male | 63 | LUAD | Ⅱ-Ⅲ | 15 | T3 | N1-3? | M0 | 3 |
| carcinoma | male | 64 | LUAD | Ⅱ | 20 |  | N0 | M0 |  |
| carcinoma | male | 78 | LUAD | Ⅲ | 29 |  | N0 | M0 |  |
| carcinoma | male | 65 | LUAD | Ⅱ | 20 | T2a | N0 | M0 | 2 |
| carcinoma | male | 75 | LUAD | Ⅰ | 15 | T1 | N0 | M0 | 1 |

Table S3. SiRNA, shRNA and sgRNA sequence information

| **Gene names** | **Sense (5’-3’)** | **Anti-sense (5’-3’)** |
| --- | --- | --- |
| H-siAMPKα1 | ATGCAAAGATAGCTGATTT | AAATCAGCTATCTTTGCAT |
| H-siAMPKα2 | TAGATGTTGTTGGAAAAATAAAA | TTTTATTTTTCCAACAACATCTA |
| M-shAMPKα2 | CCGGATCTAAACTGCGAATCTTCTGCTCGAGCAGAAGATTCGCAGTTTAGATTTTTTG | AATTCAAAAAATCTAAACTGCGAATCTTCTGCTCGAGCAGAAGATTCGCAGTTTAGAT |
| M-shPHF2 | CCGGCGTGGCTATTAAAGTGTTCTACTCGAGTAGAACACTTTAATAGCCACGCCTTTTTG | AATTCAAAAACGTGGCTATTAAAGTGTTCTACTCGAGTAGAACACTTTAATAGCCACGCC |
| H-sgRNA1-PHF2 | CACCGAGCACGTGGTACCAGGCAG | AAACCTGCCTGGTACCACGTGCTC |
| H-sgRNA2-PHF2 | CACCGTACAGGGAGATGTTGGCCG | AAACCGGCCAACATCTCCCTGTAC |

Table S4. Primers information of qPCR experiments

| **Gene names** | **Sense (5’-3’)** | **Anti-sense (5’-3’)** |
| --- | --- | --- |
| H-GAPDH | AAGAAGGTGGTGAAGCAGG | AGGTGGAGGAGTGGGTGTCG |
| H-β-actin | CACCATTGGCAATGAGCGGTTC | AGGTCTTTGCGGATGTCCACGT |
| H-CDH1 | GCCTCCTGAAAAGAGAGTGGAAG | TGGCAGTGTCTCTCCAAATCCG |
| H-CDH3 | CAGGTGCTGAACATCACGGACA | CTTCAGGGACAAGACCACTGTG |
| H-CDH2 | CCTCCAGAGTTTACTGCCATGAC | GTAGGATCTCCGCCACTGATTC |
| H-TP63 | CAGGAAGACAGAGTGTGCTGGT | AATTGGACGGCGGTTCATCCCT |
| H-CLDN4 | AGTGCAAGGTGTACGACTCGCT | CGCTTTCATCCTCCAGGCAGTT |
| H – FN1 | ACAACACCGAGGTGACTGAGAC | GGACACAACGATGCTTCCTGAG |
| H-VIM | AGGCAAAGCAGGAGTCCACTGA | ATCTGGCGTTCCAGGGACTCAT |
| H-snail1 | TGCCCTCAAGATGCACATCCGA | GGGACAGGAGAAGGGCTTCTC |
| H-IDH2 | AGATGGCAGTGGTGTCAAGGAG | CTGGATGGCATACTGGAAGCAG |

Table S5. Primers information of CHIP-qPCR experiments

| **Gene names** | **Sense (5’-3’)** | **Anti-sense (5’-3’)** |
| --- | --- | --- |
| H-GAPDH | ATCGGGCCAATCTCAGTCCCTTCCC | GCCTGGTTCAACTGGGCACGCACC |
| H-CDH1-P1 | TGTGGTGGCACACGCCTGTAG | GCCATGAGCCACTGAGCTAGCAG |
| H-CDH1-P2 | AACAAAAGAACTCAGCCAAGTG | TCTAGGTGGGTTATGGGACCT |
| H-CDH1-P3 | GCTTGCGGAAGTCAGTTCAGACT | TCCGCTCCTCAGGACCCGAAC |
| H-CDH1-P4 | CCCAGTGATGGGAGTGGGGG | CTCGACTTGCACCAGGGCACC |
| H-CDH1-P5 | GAAAGGGAACGGTGGGCTAGG | CCCTCCTGCTCACCGAAACCA |
| H-CDH3 | TGGCGCTGGACCAATCAGCA | CACGGCGAGGCTGTGGAGT |
| H-TP63 | ATCAAGAAACGCTCCGCCTC | GGTGGCACACCGTGAAGTTTC |
| H-CLDN4 | GTTGGCCTGGGCTCAGGAA | CAGAGTGCACCTTTGCACCG |

Original data of western blots in the main figures


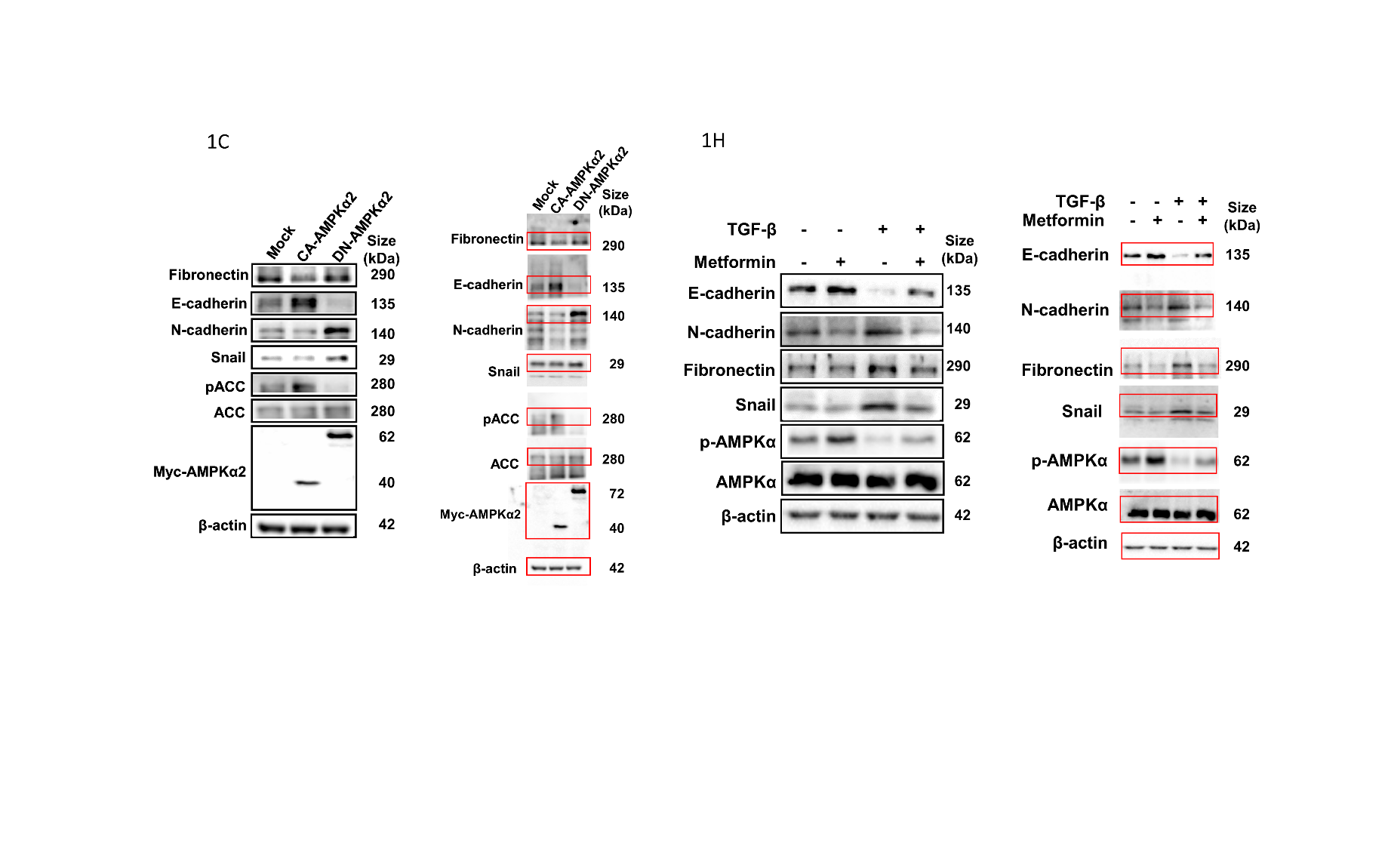

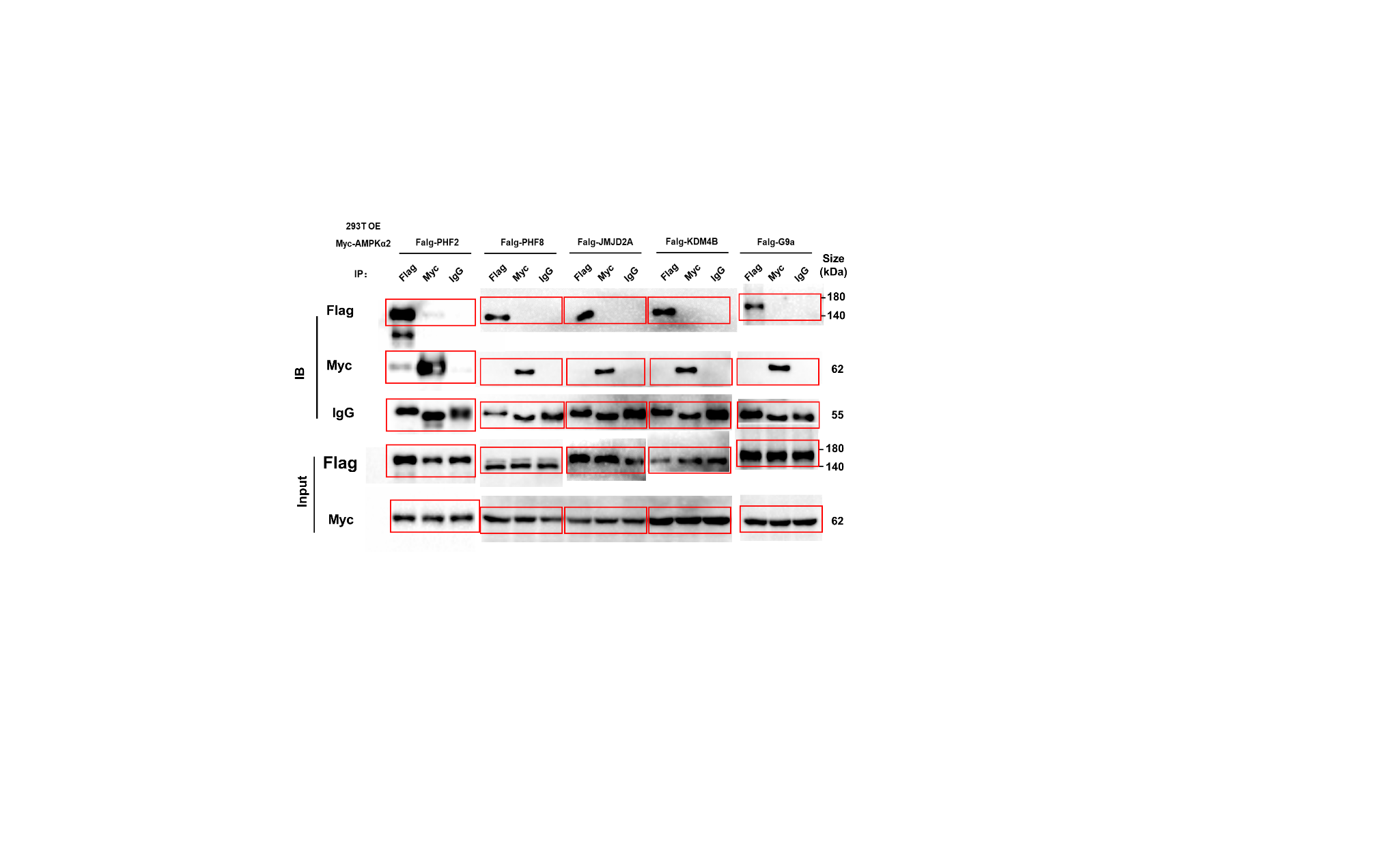


**Fig. 2c**

**Fig. 2a**

**Fig. 2b**

**Fig. 1h**

**Fig. 1j**

**Fig. 1c**


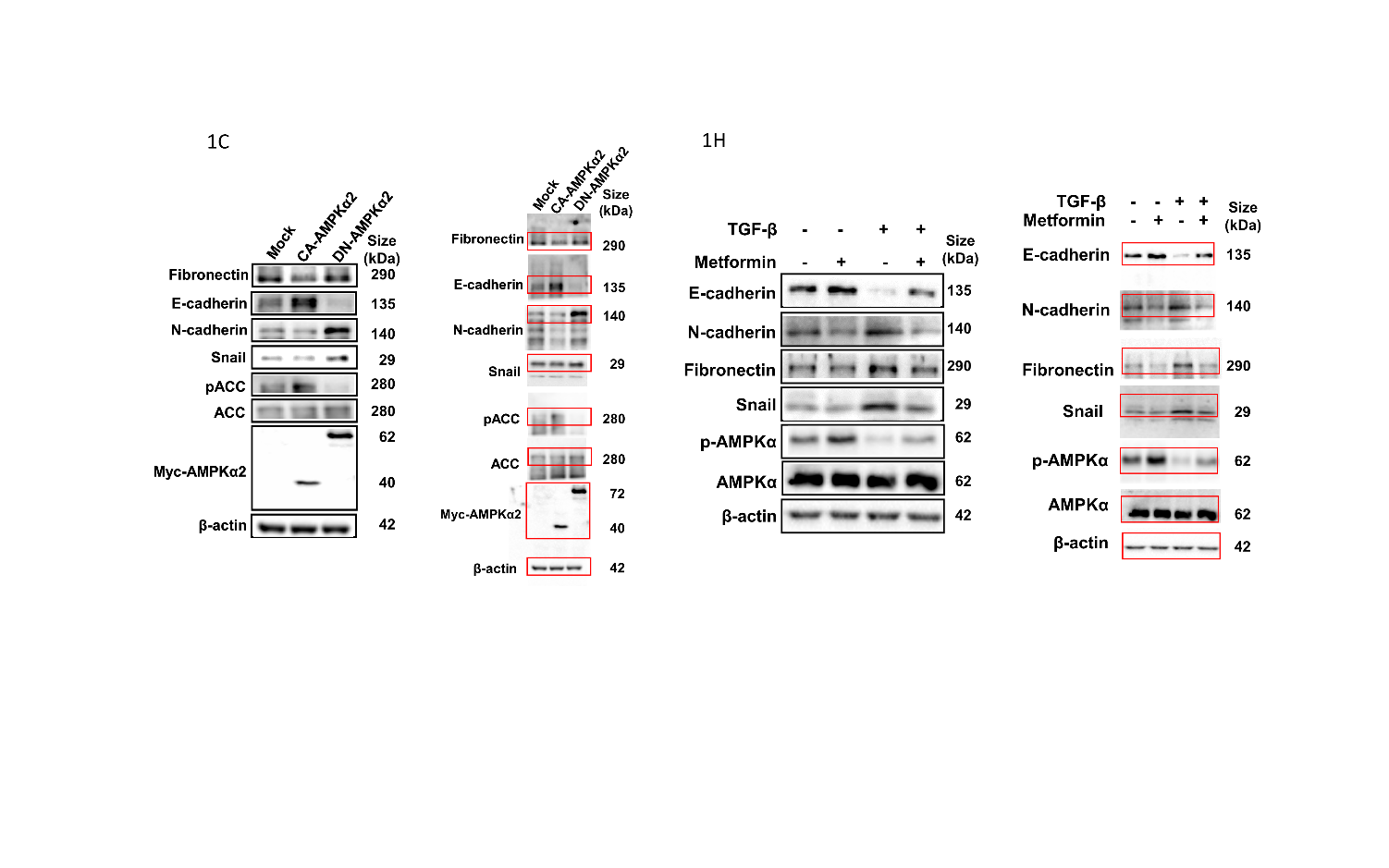

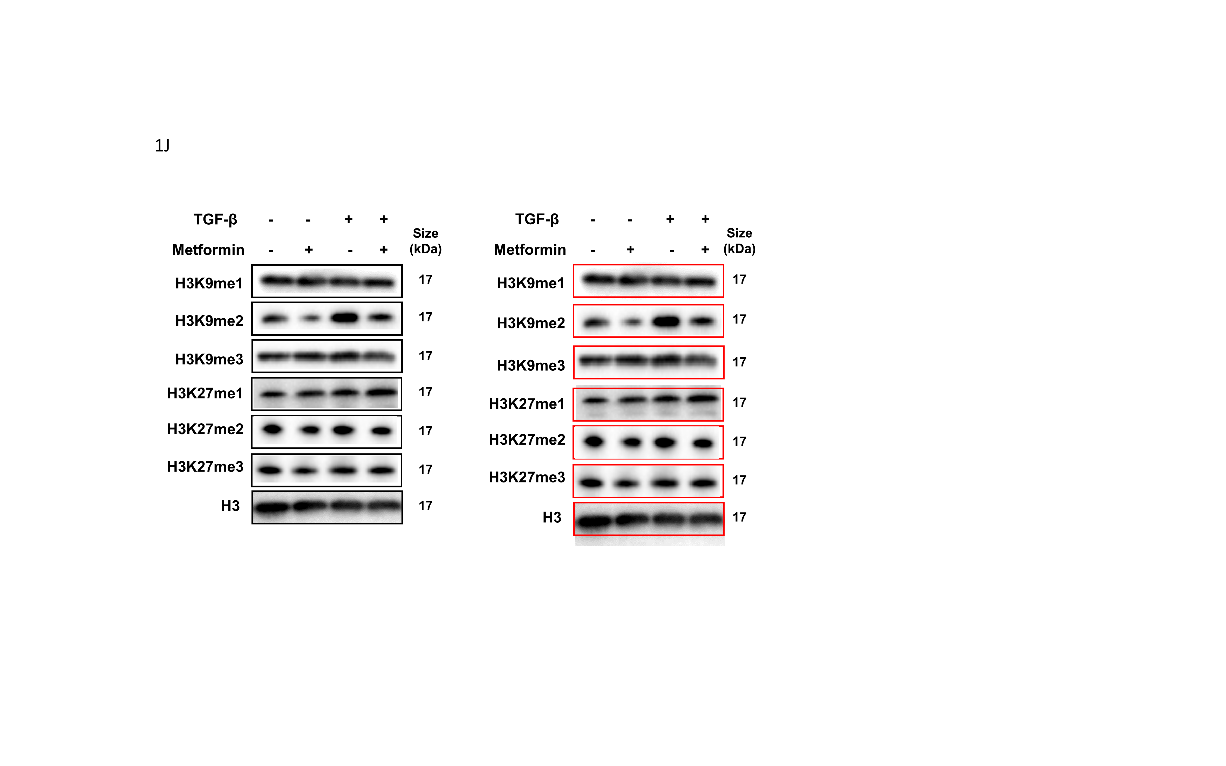


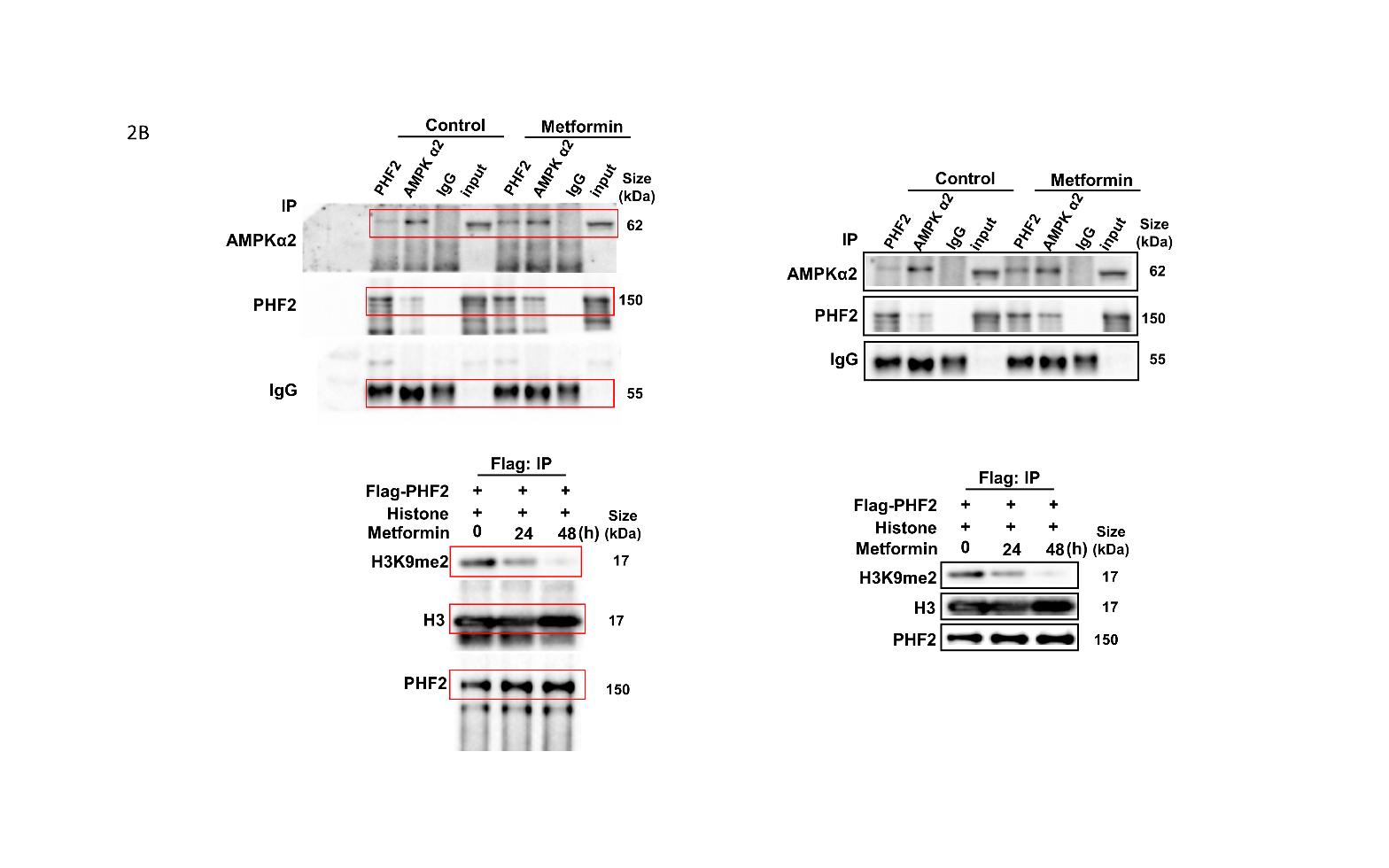


**Fig. 3a**

**Fig. 2d**


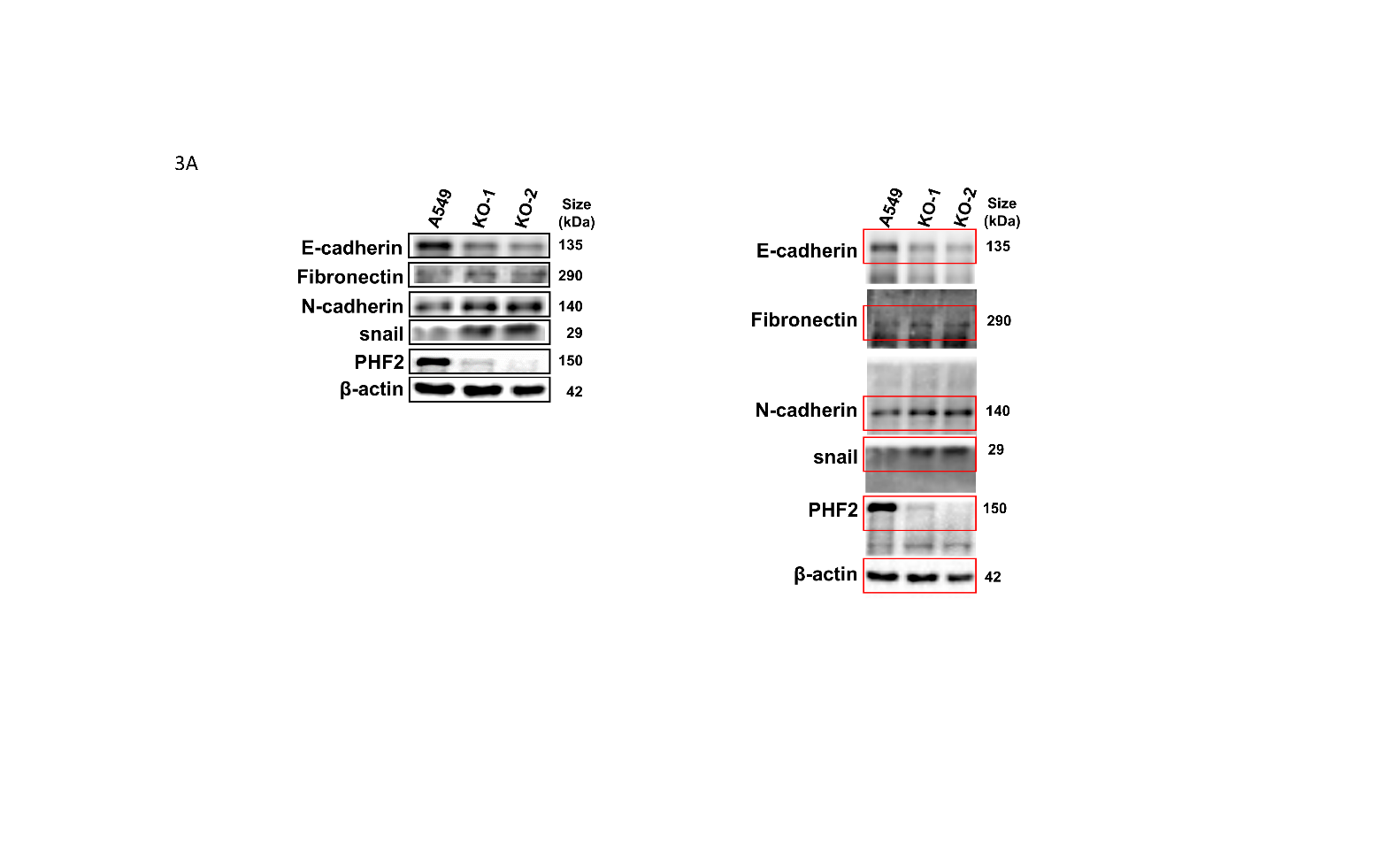


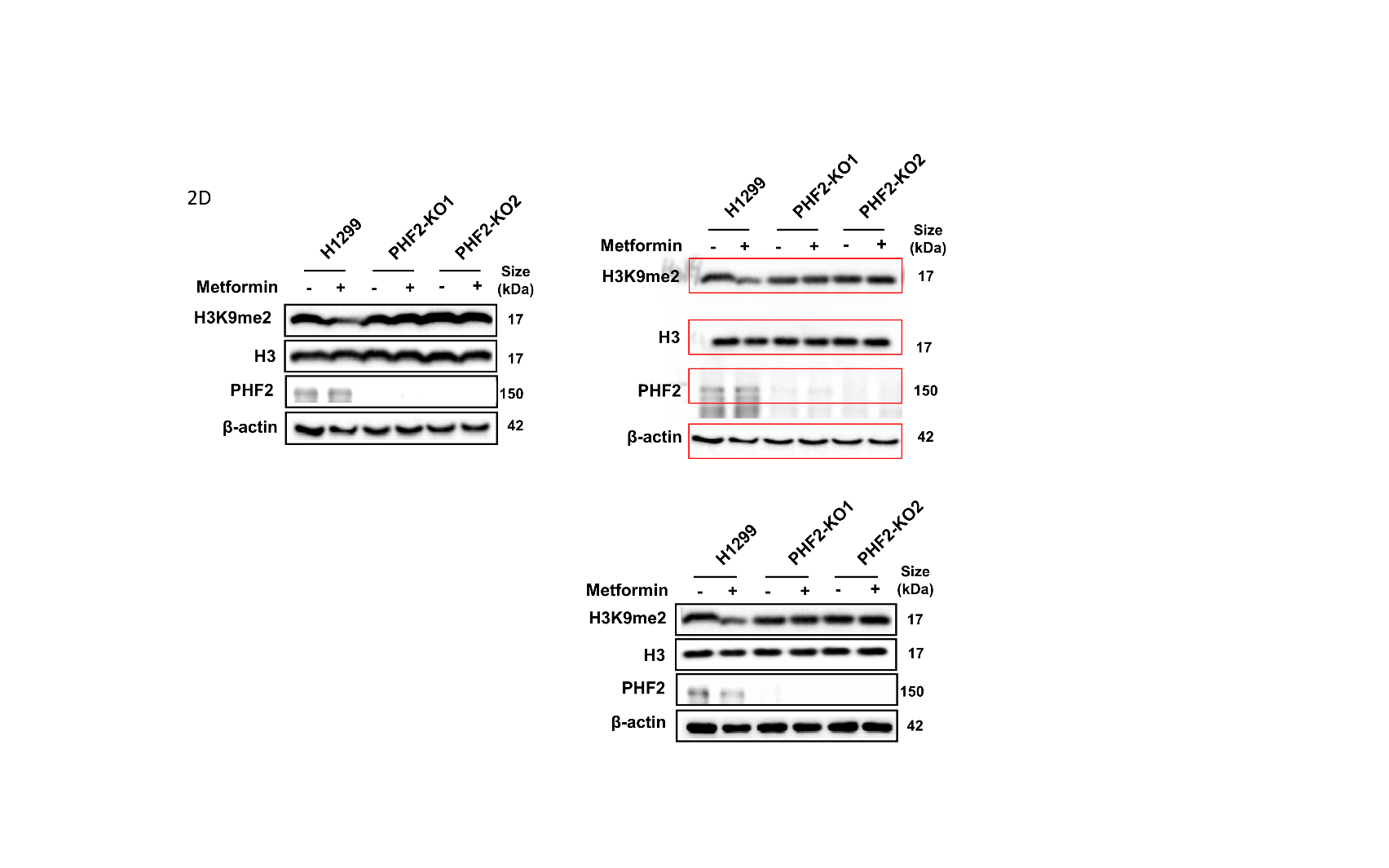

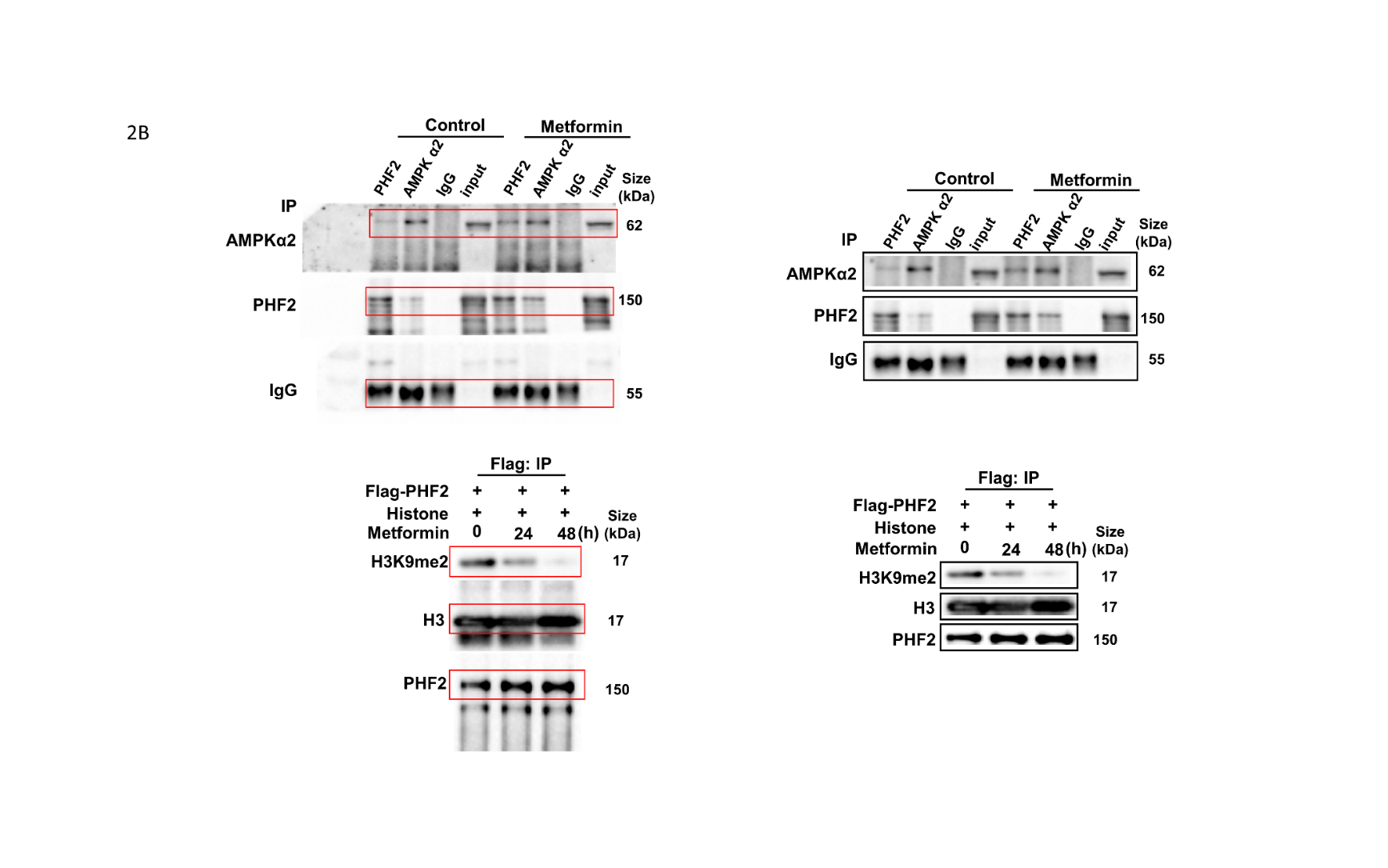


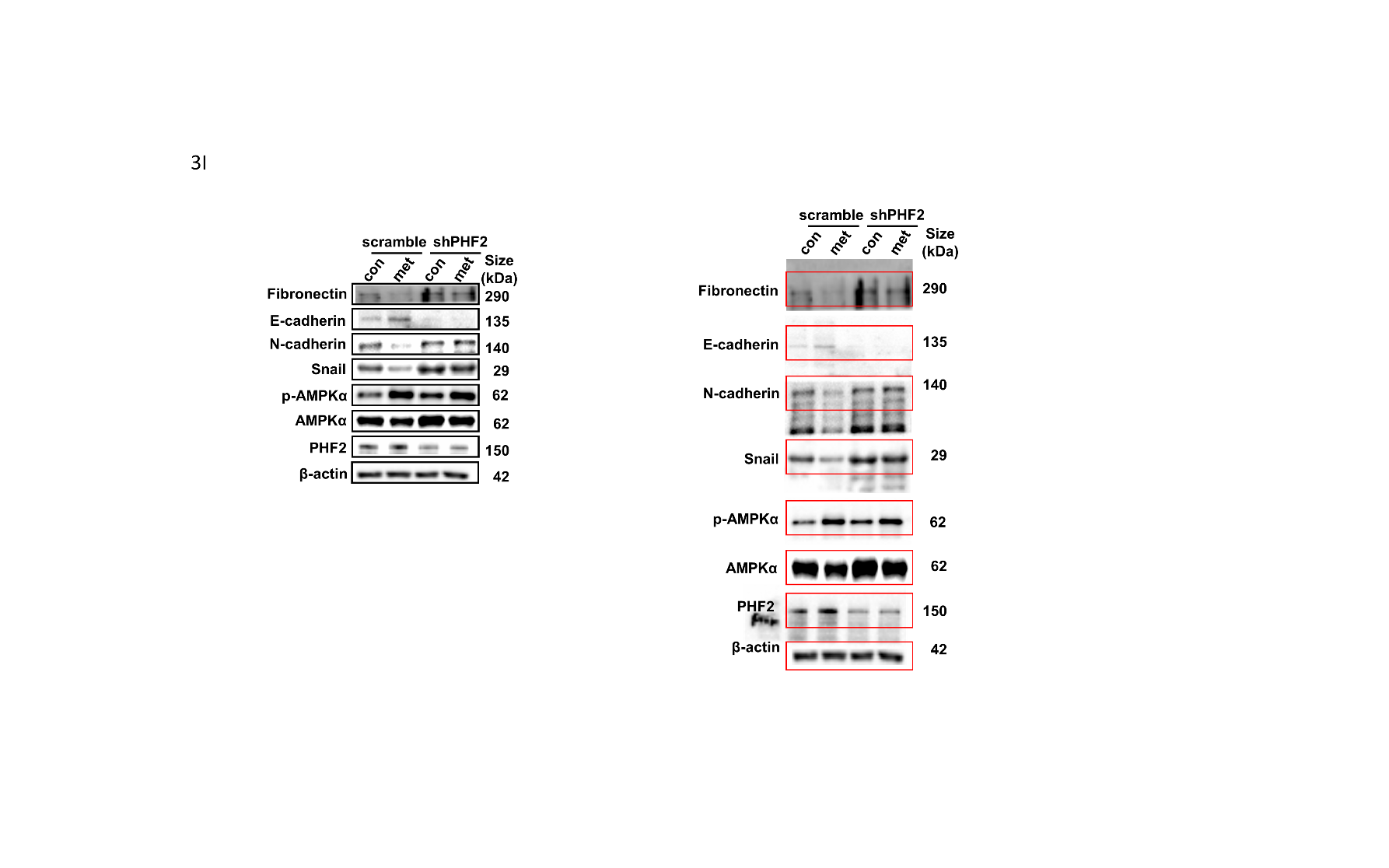


**Fig. 4c**

**Fig. 3l**


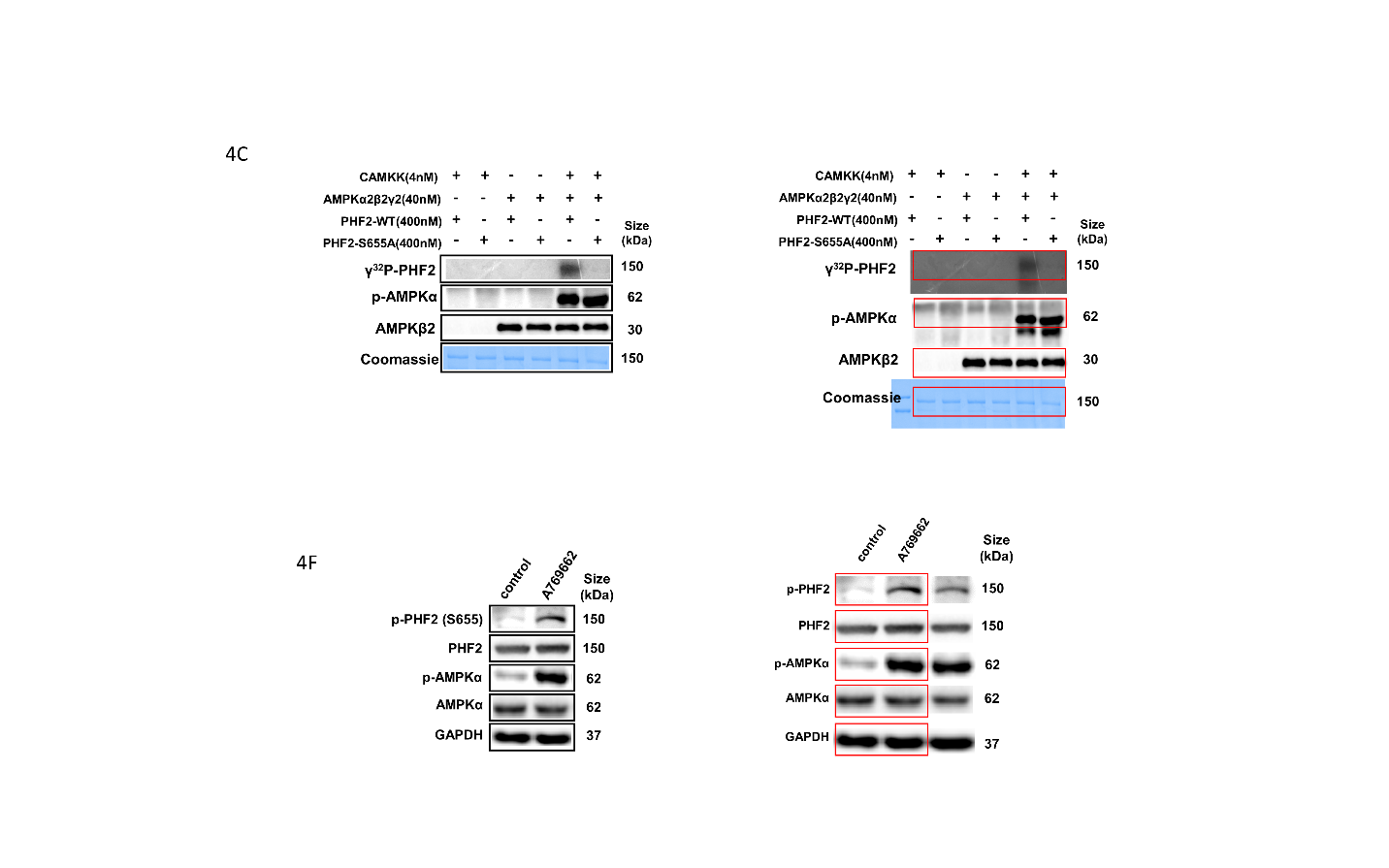


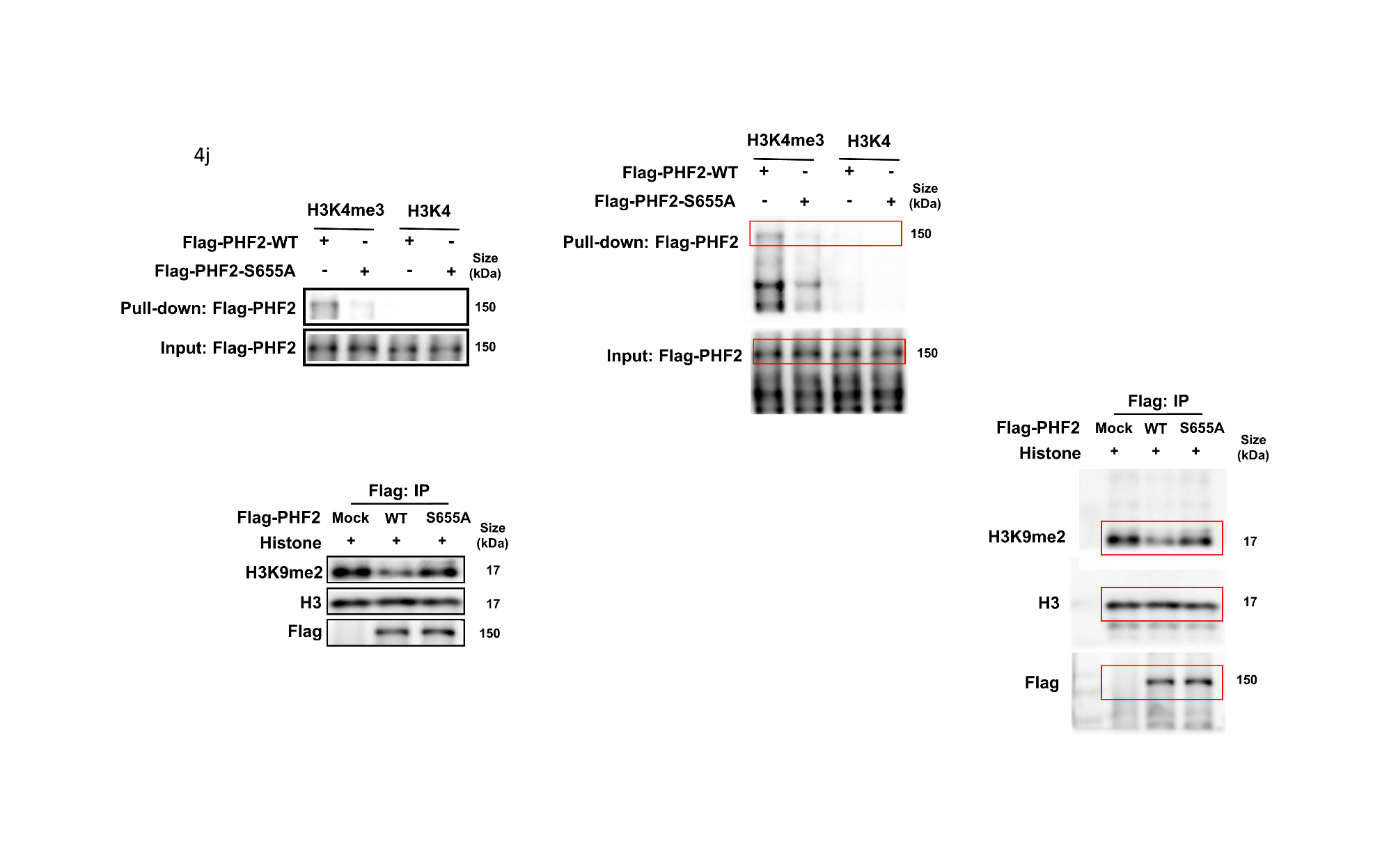

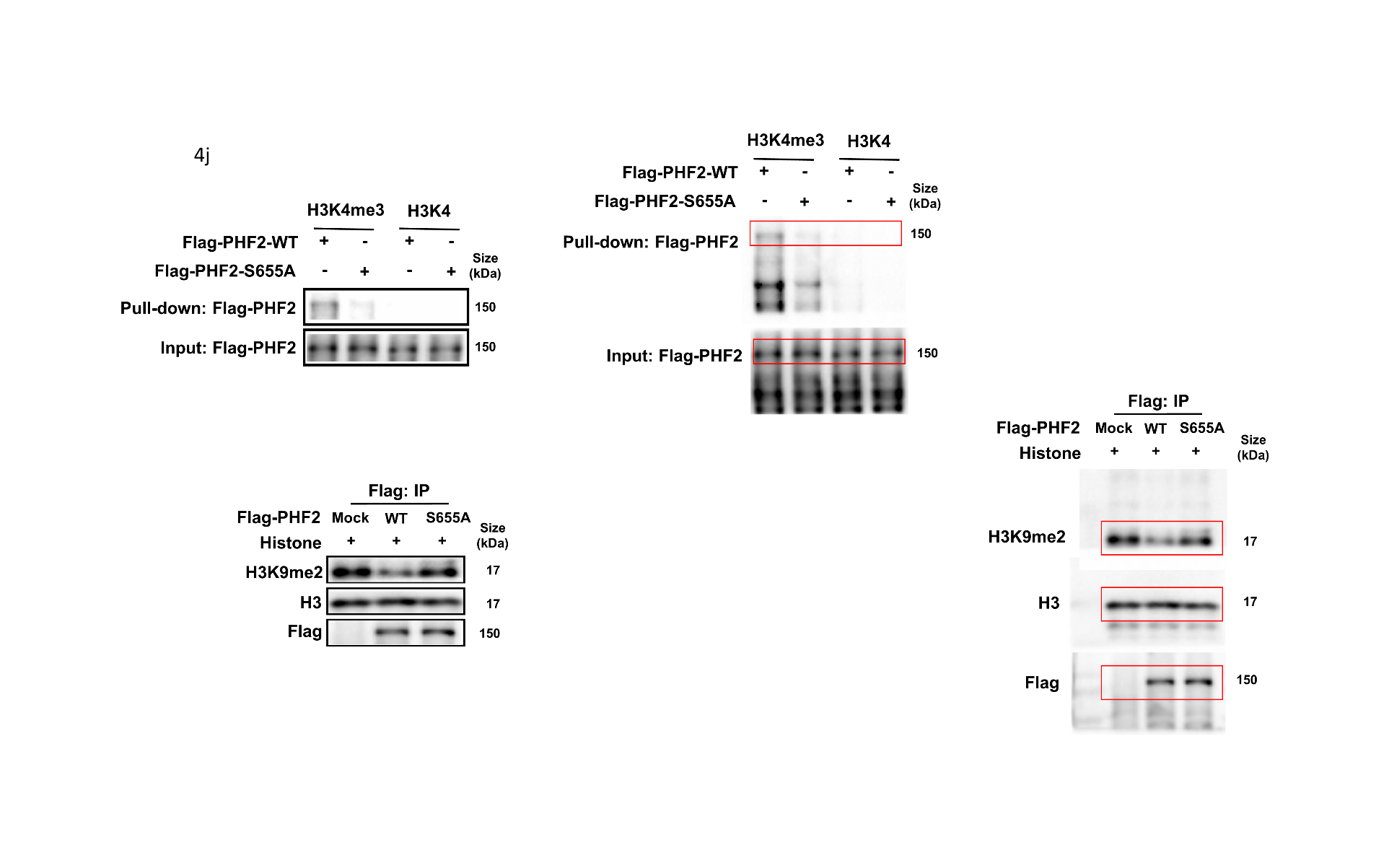

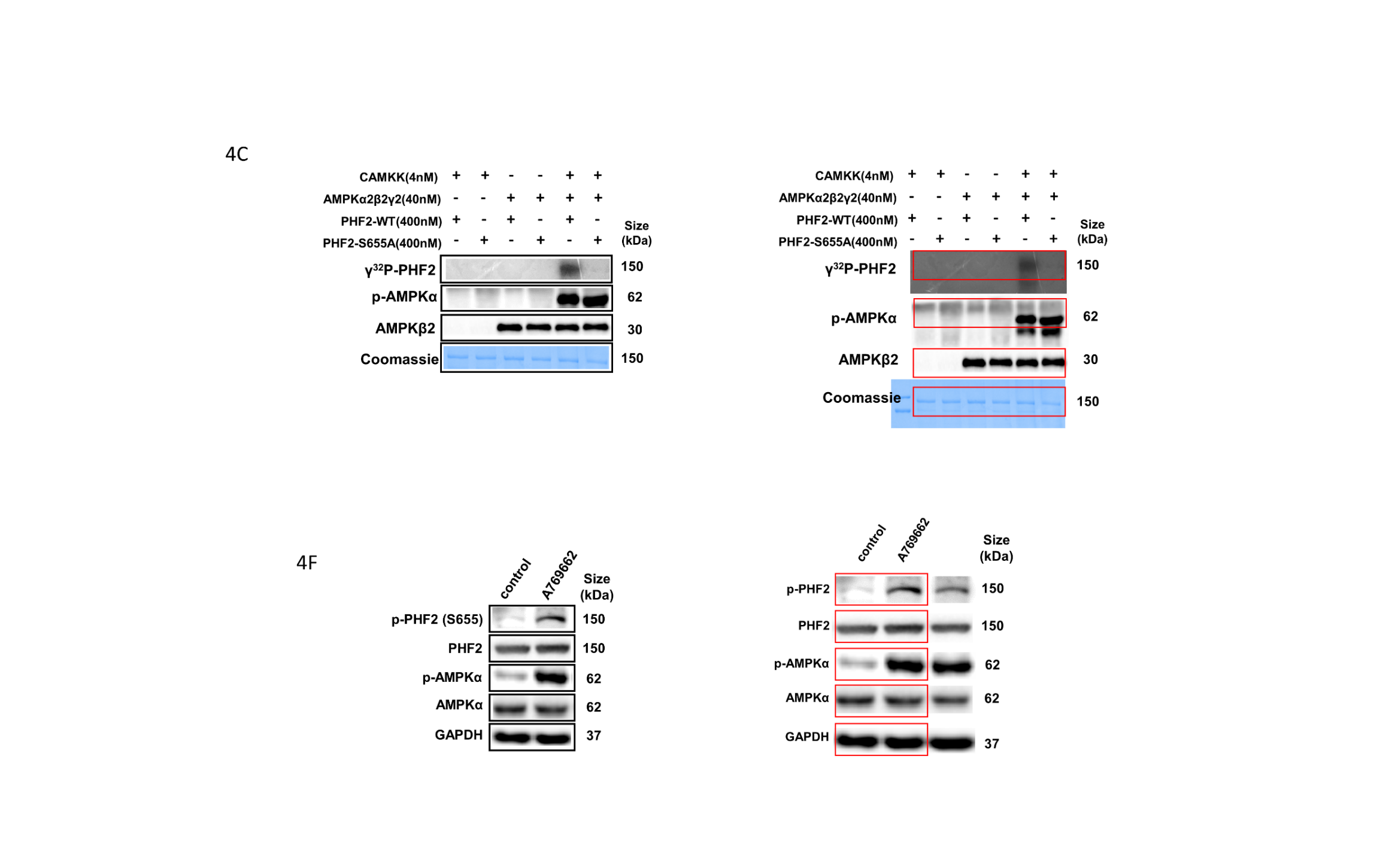

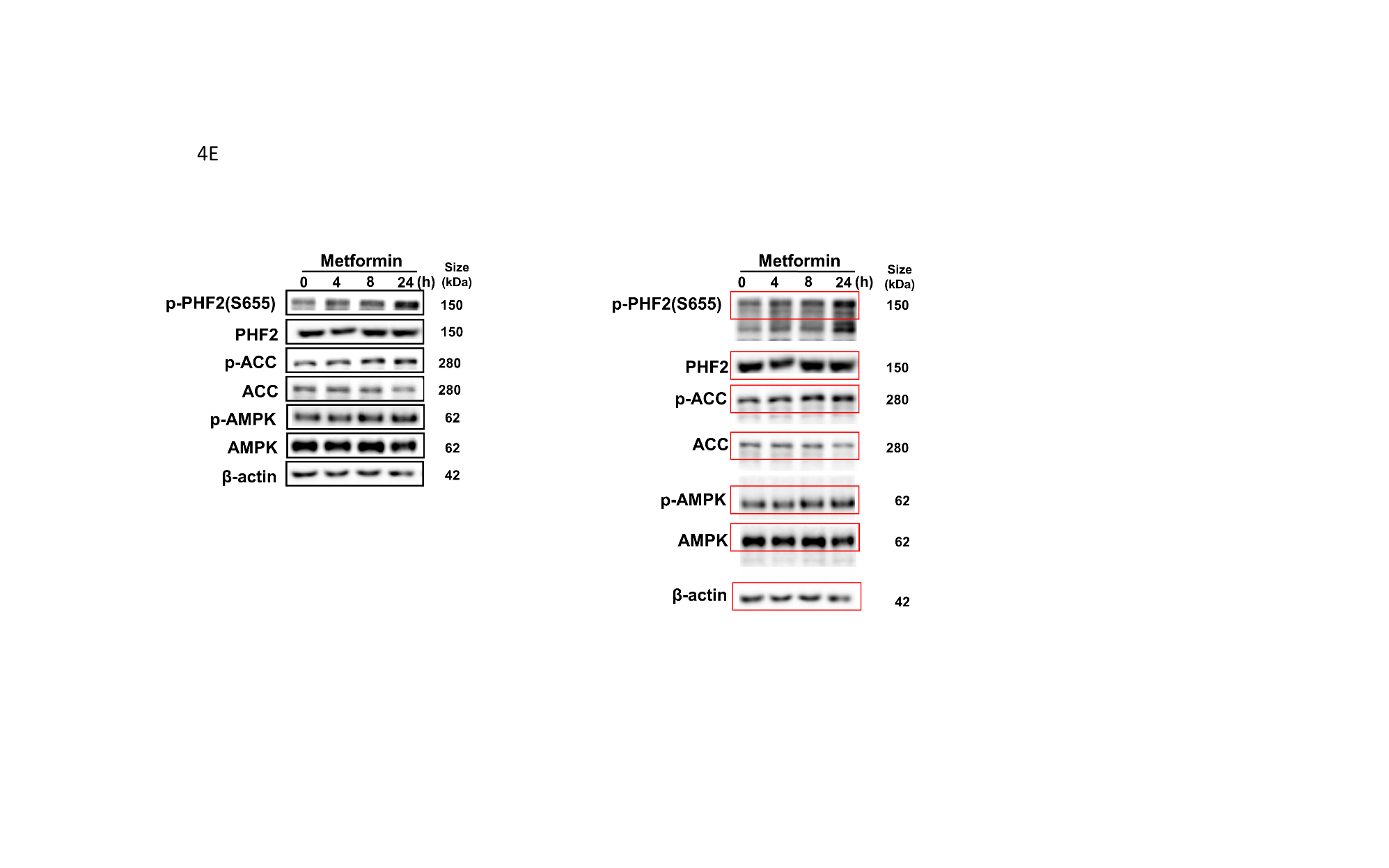

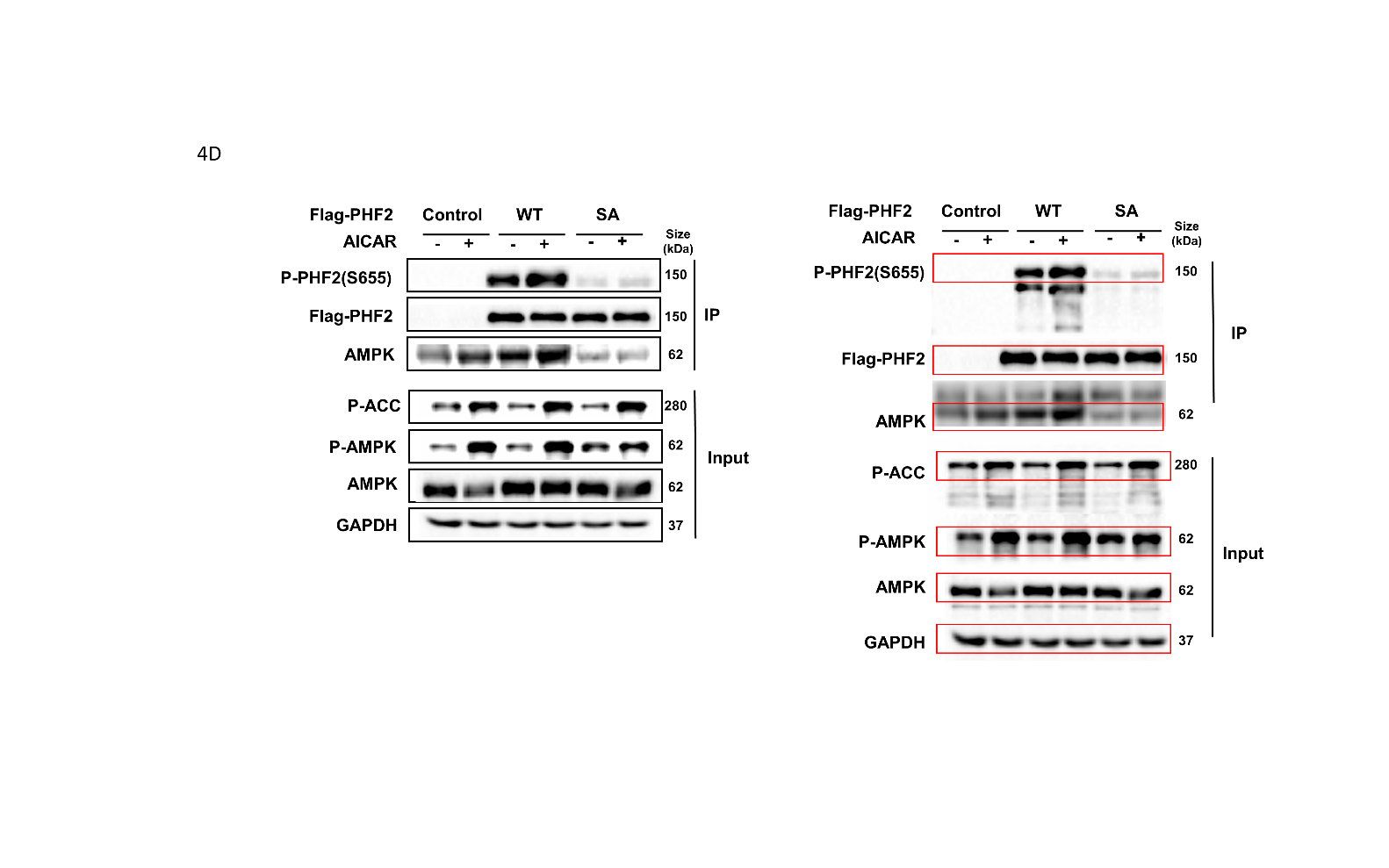
v

**Fig. 4j**

**Fig. 4f**

**Fig. 4e**

**Fig. 4d**

**Fig. 4k**

**Fig. 4l**


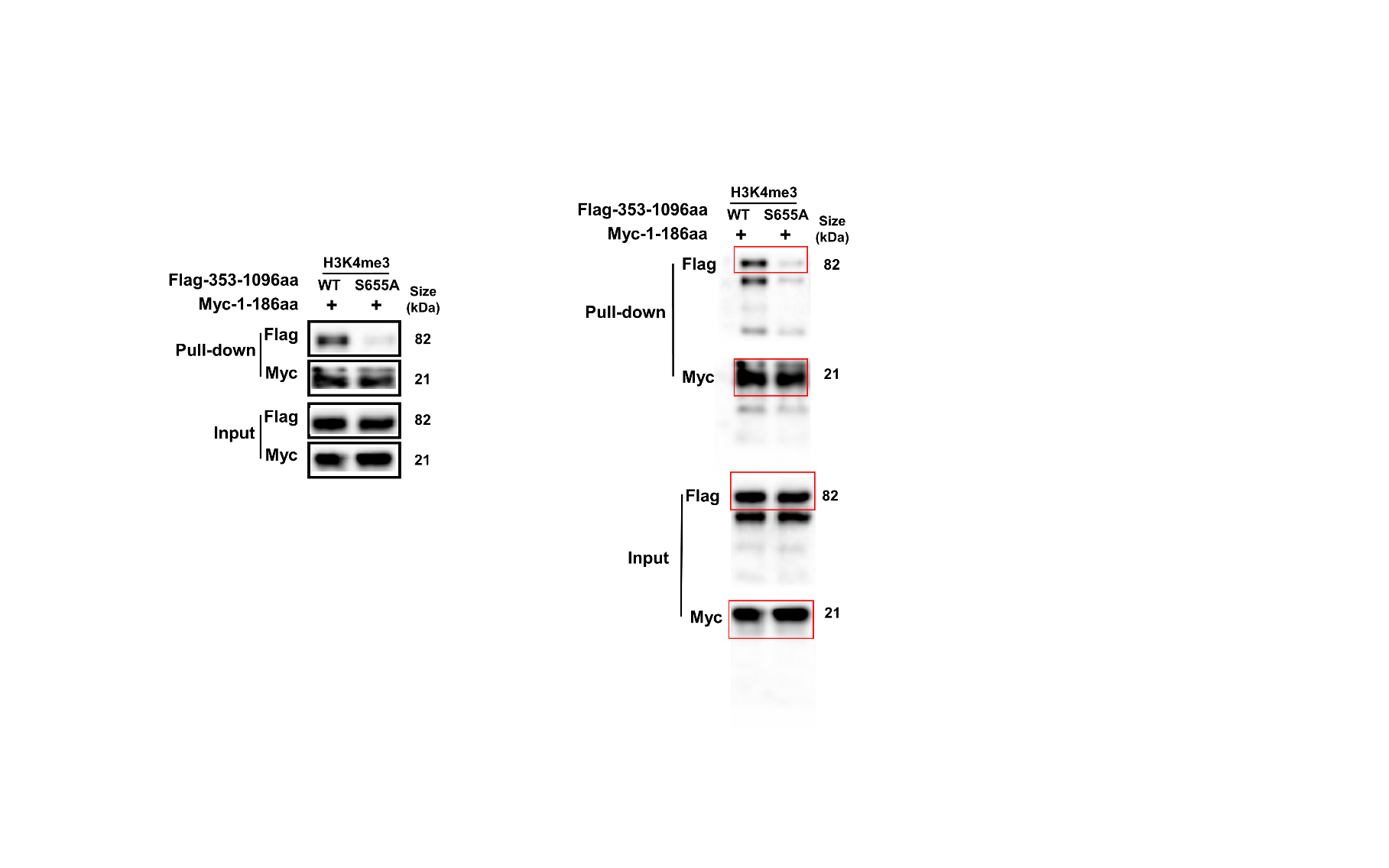


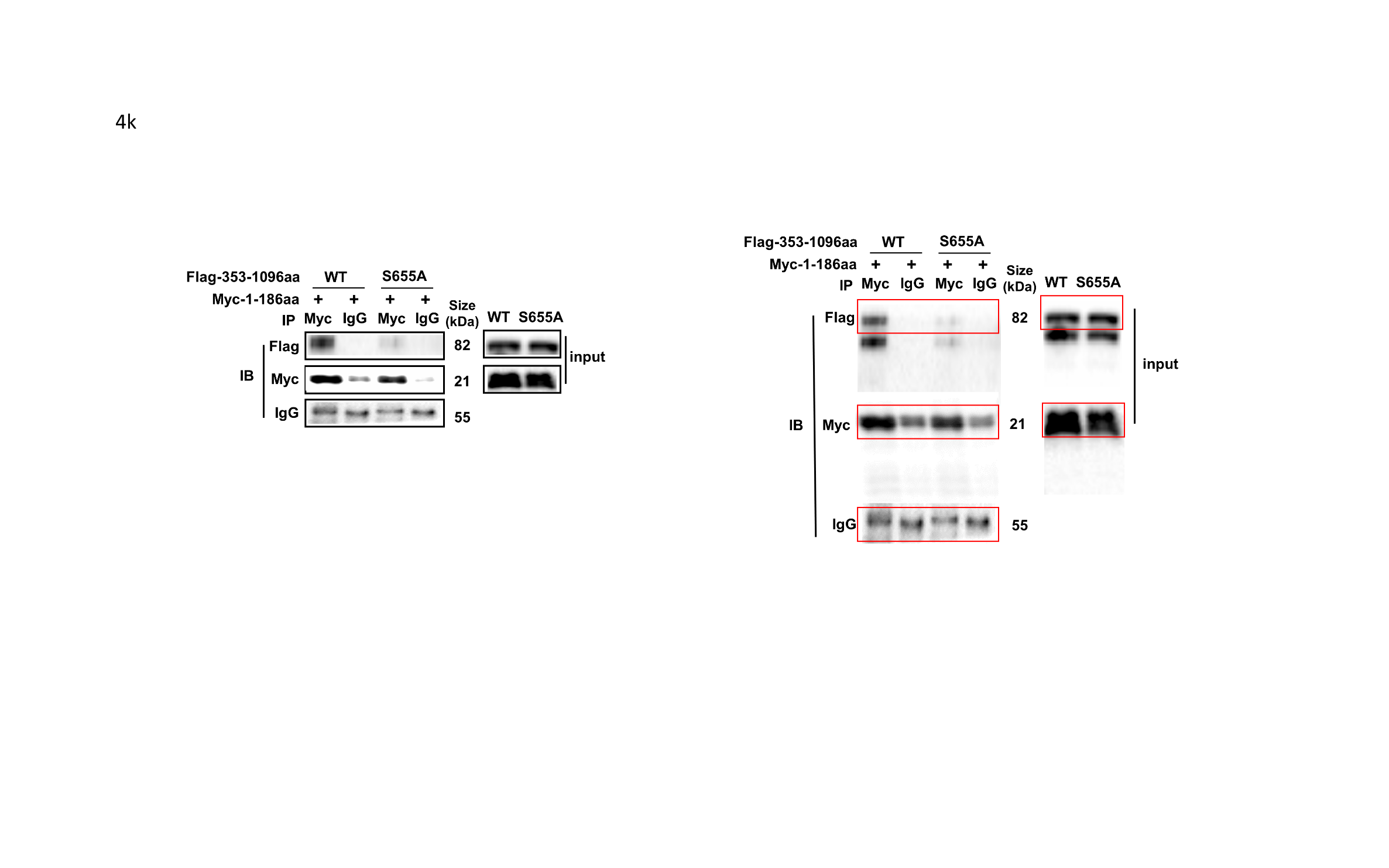


**Fig. 5a**


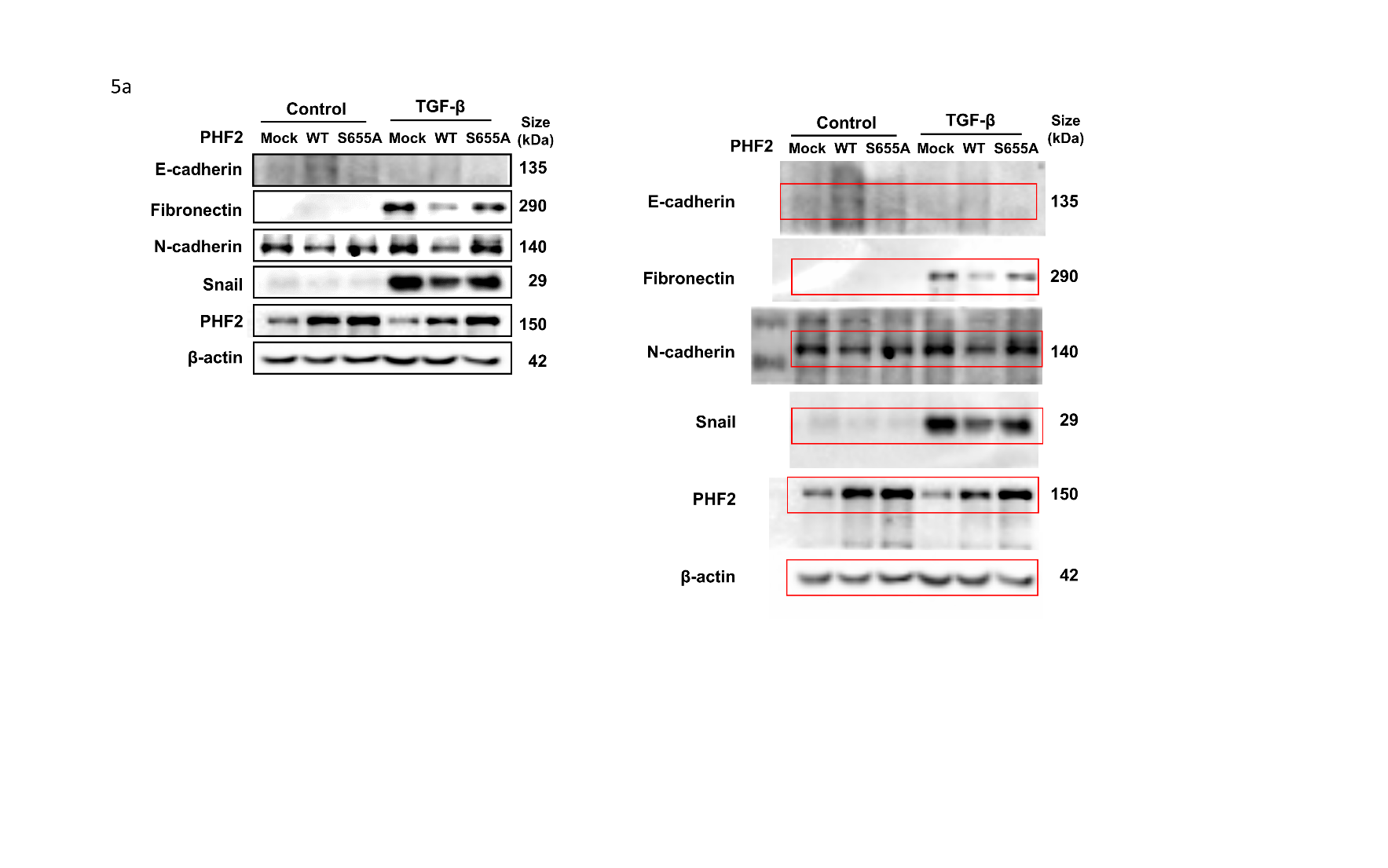

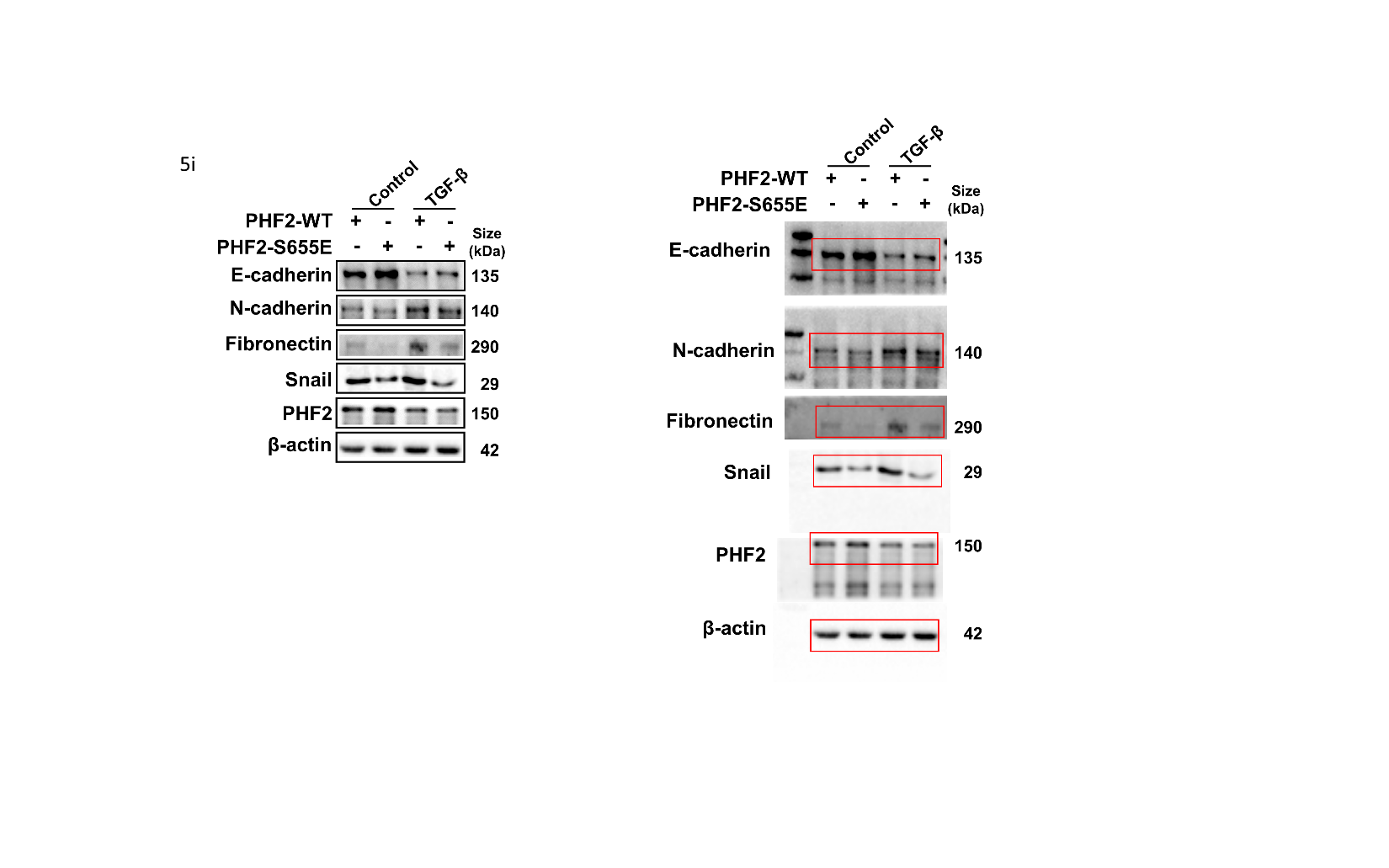


**Fig. 5i**

**Fig. 6e**


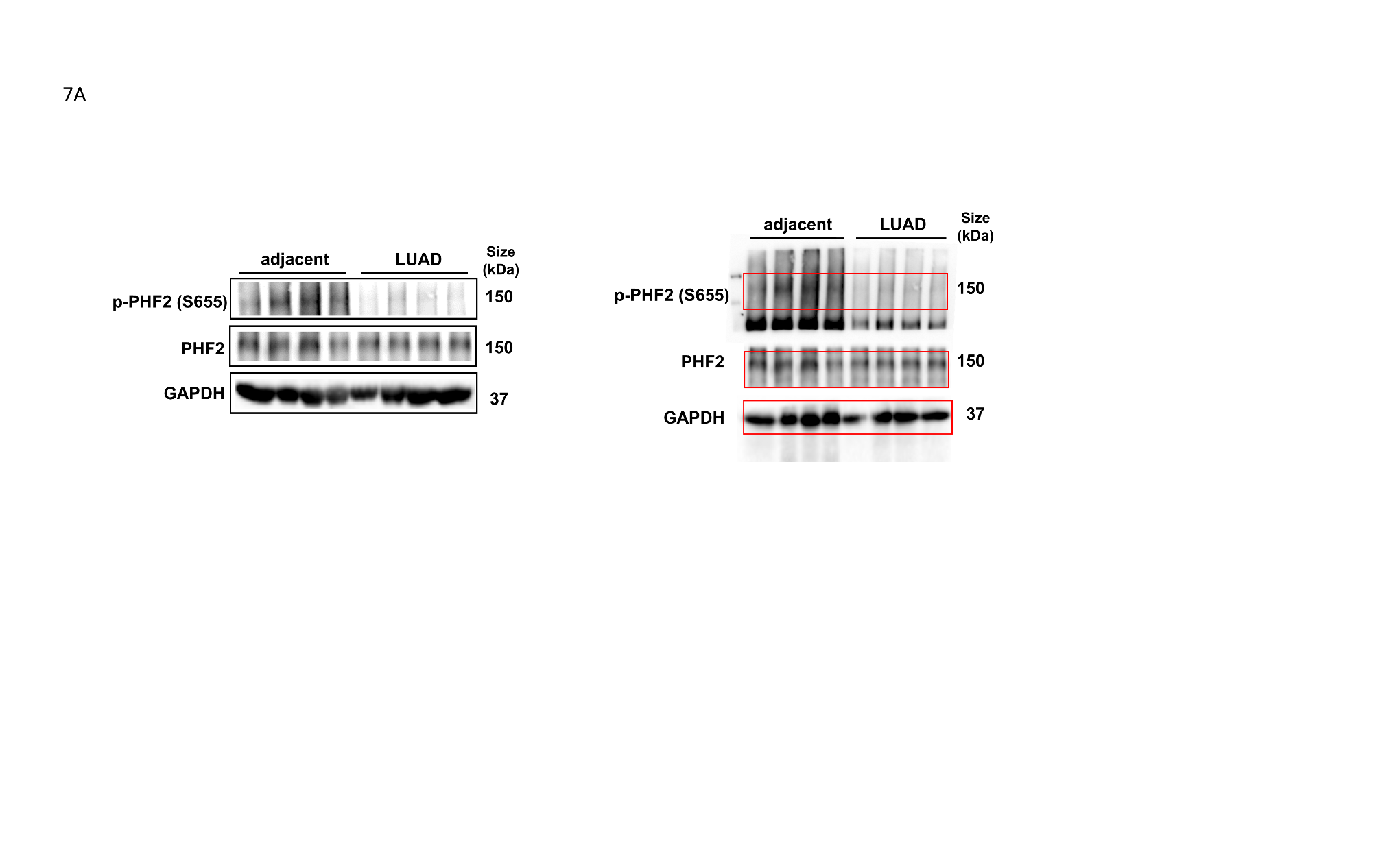

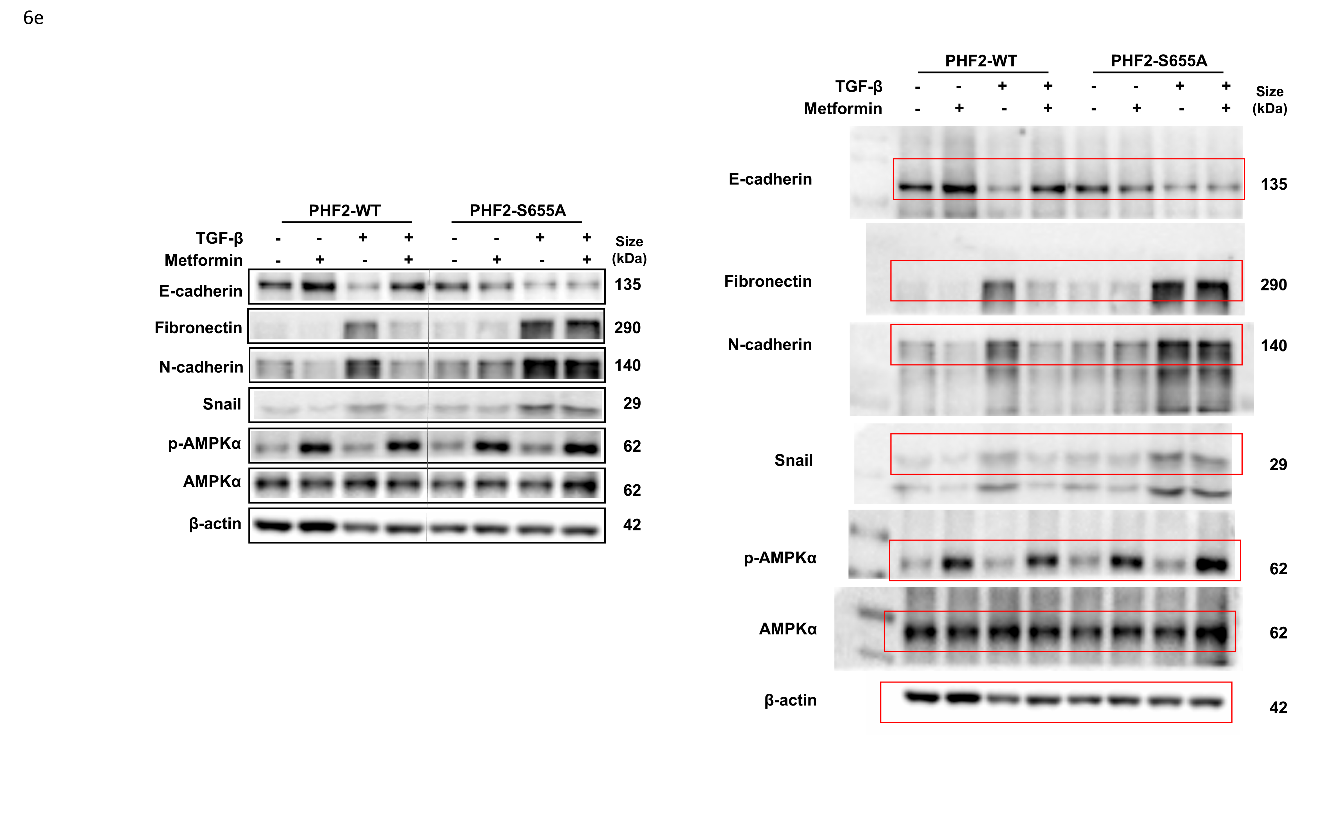

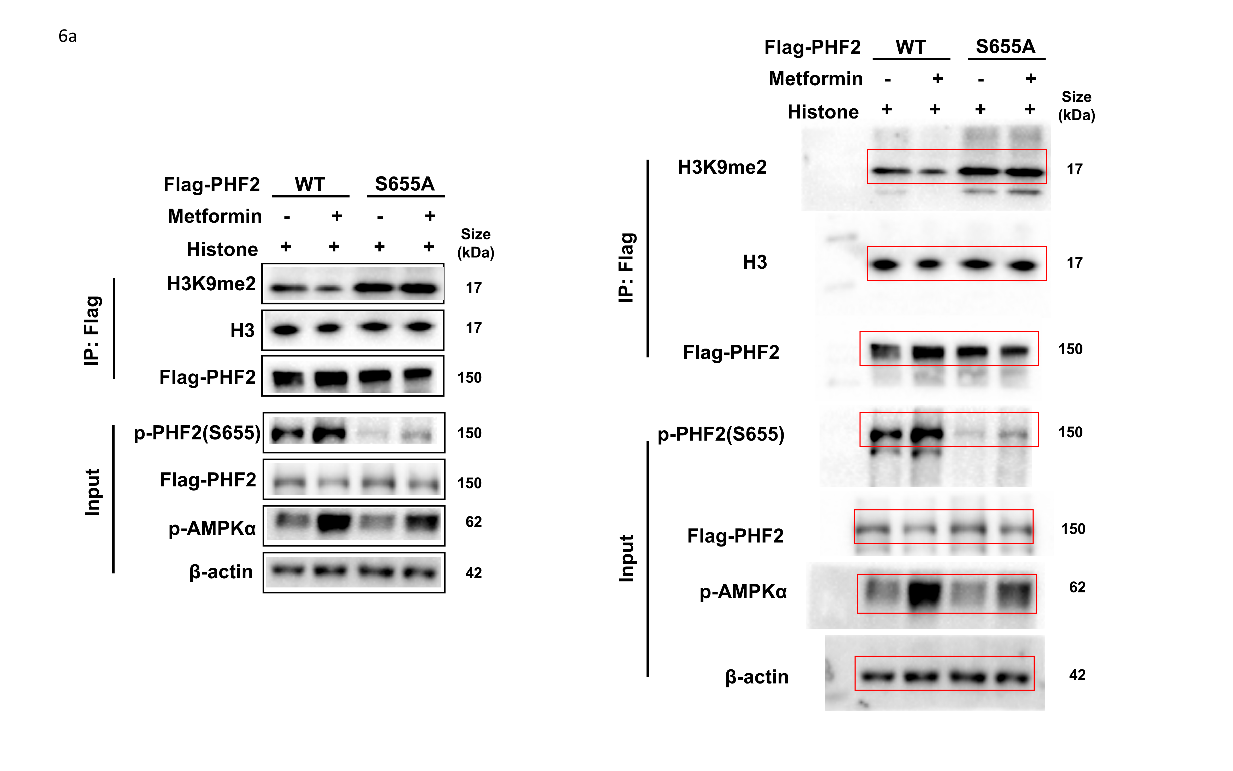


**Fig. 7a**

**Fig. 6a**
